# Designing Cu^0^−Cu^+^ dual sites for improved C−H bond fracture towards methanol steam reforming

**DOI:** 10.1038/s41467-023-43679-0

**Published:** 2023-12-02

**Authors:** Hao Meng, Yusen Yang, Tianyao Shen, Zhiming Yin, Lei Wang, Wei Liu, Pan Yin, Zhen Ren, Lirong Zheng, Jian Zhang, Feng-Shou Xiao, Min Wei

**Affiliations:** 1 State Key Laboratory of Chemical Resource Engineering, Beijing Advanced Innovation Center for Soft Matter Science and Engineering, Beijing University of Chemical Technology, Beijing, 100029 PR China; 2 Quzhou Institute for Innovation in Resource Chemical Engineering, Quzhou, 324000 PR China; 3 Institute of High Energy Physics, Chinese Academy of Sciences, Beijing, 100049 PR China; 4 Key Lab of Biomass Chemical Engineering of Ministry of Education, College of Chemical and Biological Engineering, Zhejiang University, Hangzhou, 310027 PR China

**Keywords:** Heterogeneous catalysis, Energy, Catalytic mechanisms

## Abstract

Copper-based catalysts serve as the predominant methanol steam reforming material although several fundamental issues remain ambiguous such as the identity of active center and the aspects of reaction mechanism. Herein, we prepare Cu/Cu(Al)O_*x*_ catalysts with amorphous alumina-stabilized Cu_2_O adjoining Cu nanoparticle to provide Cu^0^−Cu^+^ sites. The optimized catalyst exhibits 99.5% CH_3_OH conversion with a corresponding H_2_ production rate of 110.8 μmol s^−1^ g_cat_^−1^ with stability over 300 h at 240 °C. A binary function correlation between the CH_3_OH reaction rate and surface concentrations of Cu^0^ and Cu^+^ is established based on kinetic studies. Intrinsic active sites in the catalyst are investigated with in situ spectroscopy characterization and theoretical calculations. Namely, we find that important oxygen-containing intermediates (CH_3_O* and HCOO*) adsorb at Cu^0^−Cu^+^ sites with a moderate adsorption strength, which promotes electron transfer from the catalyst to surface species and significantly reduces the reaction barrier of the C−H bond cleavage in CH_3_O* and HCOO* intermediates.

## Abstract (web summary)

Cu-based catalysts are widely considered to be effective materials for H_2_ production via methanol steam reforming. Herein, the authors explore a series of catalysts containing Cu^0^−Cu^+^ dual sites for facilitating C-H bond breaking and H_2_ evolution.

## Introduction

With increasing resource and environmental challenges, hydrogen is regarded as a potential substitution for fossil energy to a cleaner energy landscape, which has been extensively applied in polymer electrolyte membrane (PEM) fuel cells^1^. However, its practical popularization has been filled with difficulties, especially in respect to hydrogen fuel transport and storage owing to its high risk and the low volume energy density^2,3^. Compared with gaseous H_2_ storage, in situ production of hydrogen from liquid fuel, such as methanol (CH_3_OH), not only eliminates safety risks of high-pressure hydrogen storage, but also reduces transportation costs, which provides an alternative solution for fuel cell system application^4,5^. Notably, methanol steam reforming (MSR) serves as one cost-effective way due to the reasonable energy utilization and facile process control, which offers a high yield of hydrogen at relatively mild reaction conditions^6–8^. MSR as a tandem reaction, which involves complex routes including methanol dehydrogenation, steam reforming and intermediate decomposition, has attracted considerable interest in energy chemistry and heterogeneous catalysis^9,10^.

Among various catalysts used in MSR, Cu is an appropriate active ingredient with advantages of cost-effectiveness, satisfactory low-temperature activity, and high H_2_ selectivity^7,11–13^. Previous studies have focused on tuning Cu crystal structure, doping extra metal constituents (e.g., Ni, Fe, and Co)^14–17^, or immobilization on metal oxides supports (e.g., SiO_2_, Al_2_O_3_, TiO_2_, CeO_2_, ZnO and ZrO_2_)^7,12,18–23^, so as to boost catalytic performance. Compared with the rapid development of application research, the fundamental scientific insights (such as the intrinsic active sites, adsorption behavior and reaction mechanism) of this reaction have not been well solved because of the complex catalyst structure and intricate reaction processes, which could lead to uncertain and even contradictory conclusions. Due to the rich redox properties of copper, various Cu species (Cu^0^, Cu^*δ*+^/Cu^+^) normally coexists under actual reaction conditions^23–26^; moreover, the effects of electron rearrangement in alloy, strong metal-support interaction (SMSI) and oxygen vacancy induction make this issue rather complicated^18–22,27,28^, which is prone to cause ambiguous relationship between catalytic performance and microscopic structure. Therefore, a detailed study based on spatially and temporally-resolved *operando* characterization techniques, kinetic investigations as well as theoretical calculations is imperative to shed light on the intrinsic active sites, structure-activity correlation, and reaction mechanism. An in-depth understanding of these fundamental issues would not only provide rational criteria for the structure design of heterogeneous catalysts, but also promote further progress of applied technology.

Inspired by the above though, herein, a series of *y*Cu/Cu(Al)O_*x*_ samples (*y* denotes the mass ratio of Cu/Al) with tunable synergistic Cu^0^−Cu^+^ sites were prepared through a co-precipitation method followed by subsequent reduction treatment, which were characterized by amorphous alumina-stabilized Cu_2_O adjoining Cu nanoparticle. Typically, the 4.25Cu/Cu(Al)O_*x*_ sample exhibits the optimal catalytic performance with *a* > 99% methanol conversion and a high H_2_ production rate (110.8 μmol s^−1^ g_cat_^−1^), which is preponderant to the previously reported Cu-based catalysts for MSR. The kinetic studies, in situ FT-IR spectroscopy and mass spectrometry analysis substantiate that the MSR reaction follows three main processes: dehydrogenation of CH_3_OH, hydrolysis of HCOOCH_3_ and decomposition of HCOO*, where the cleavage of C−H bonds in CH_3_O* and HCOO* intermediates is the rate-determining step. As revealed by STEM-EELS, isotope dynamics measurements, in situ FT-IR spectra, in situ XAFS spectra and DFT calculation, the synergistic effect between Cu^0^ and Cu^+^ species derived from Cu-Cu(Al)O_*x*_ boundary plays a crucial role: the oxygen-containing intermediates (CH_3_O* and HCOO*) undergo activation adsorption at Cu^0^−Cu^+^ interfacial sites via oxygen-end bridge with a moderate strength. This unique adsorption configuration promotes electron transfer from catalyst surface to reaction intermediates and significantly reduces the energy barrier of C−H bonds cleavage (rate-determining step) in CH_3_O* and HCOO* intermediates, accounting for the exceptional activity of 4.25Cu/Cu(Al)O_*x*_ catalyst.

## Results

### Structural characterizations

**Fig. 1:**
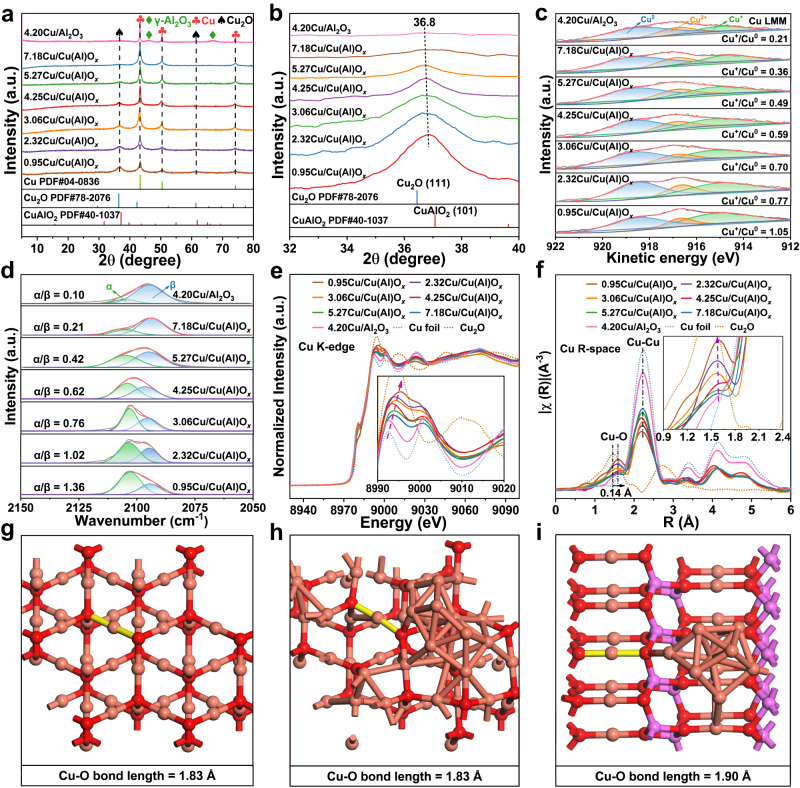
Fine structure characterizations of various samples. **a**, **b** XRD patterns, **c** *quasi-*in situ Cu LMM AES spectra, **d** CO-DRIFT spectra, **e** Cu K-edge XANES and **f** Cu K-edge EXAFS spectra of *y*Cu/Cu(Al)O_*x*_ and control samples. Cu−O bond length in **g** Cu_2_O(111), **h** Cu(111)/Cu_2_O(111) and **i** Cu(111)/CuAlO_2_(101) based on DFT calculations (red: O; orange: Cu; purple: Al).

The *y*Cu/Cu(Al)O_*x*_ samples were prepared via a co-precipitation method, followed by the subsequent roasting and reduction processes (Supplementary Figs. 1 and 2). As shown in Supplementary Fig. 3, the XRD patterns of catalyst precursors (*y*CuAlO_*x*_ samples) show characteristic diffraction peaks indexed to a CuO phase (PDF#48-1548). Then, the reduction of *y*CuAlO_*x*_ in a H_2_ atmosphere results in the formation of *y*Cu/Cu(Al)O_*x*_ samples, in which both Cu (PDF#04-0836) and Cu_2_O (PDF#78-2076) phases are observed (Fig. 1a). The absence of Al_2_O_3_ reflections indicates an amorphous phase. For the control sample 4.20Cu/Al_2_O_3_, the reflections ascribed to Cu and *γ*-Al_2_O_3_ phases are present, without Cu_2_O species. When the Cu/Al ratio increases from 0.95 to 7.18, the relative peak intensity of metallic Cu enhances gradually whilst that of Cu_2_O declines accordingly, which indicates that the decrease of Al content favors the transformation from Cu^+^ to Cu^0^ during H_2_ reduction. This is in good agreement with XRD Rietveld refinement analysis (Supplementary Figs. 4−10). Notably, compared with the standard Cu_2_O(111) lattice plane (36.4°), the Cu_2_O(111) reflection in these samples shifts to a higher diffraction angle (36.8°, Fig. 1b) and is located between Cu_2_O(111) and CuAlO_2_(101) (PDF#40-1037), which indicates that partial Cu^+^ atoms can be stabilized by amorphous Al_2_O_3_ at the interfacial sites to form a CuAlO_2_-like structure.

Furthermore, H_2_-TPR measurements on these *y*CuAlO_*x*_ samples were carried out to investigate the structural evolution from catalyst precursor to *y*Cu/Cu(Al)O_*x*_. As shown in Supplementary Fig. 11, the H_2_ consumption peaks at lower temperature (*α*, 150−200 °C) and higher temperature (*β*, 200−300 °C) are attributed to the reduction of copper oxide species that weakly and strongly interacts with alumina, respectively. As the Cu/Al ratio increases from 0.95 to 7.18, the intensity of α reduction peak increases whilst β reduction peak decreases gradually, which is consistent with the variation tendency of Cu and Cu_2_O species in XRD results (Fig. 1a).

The surface chemical states of *y*Cu/Cu(Al)O_*x*_ samples were measured by *quasi-*in situ XPS and AES spectra (Supplementary Figs. 12−14). The Cu 2*p*_3/2_ XPS spectra confirm the co-existence of Cu^+^/Cu^0^ species (932.2–932.5 eV) with less than 20% Cu^2+^ (934.7–934.9 eV) for all these samples (Supplementary Fig. 12)^20,23^. Auger Cu LMM spectra further verify that the surface Cu^+^/Cu^0^ ratio decreases successively as the Cu/Al ratio increases from 0.95 to 7.18 (Fig. 1c and Supplementary Table 1)^29,30^. In contrast, the control sample 4.20Cu/Al_2_O_3_ shows the minimal value of Cu^+^/Cu^0^ ratio. Furthermore, in situ CO-DRIFTS was performed with CO as the probe molecule to study the surface chemical valence of Cu species. As shown in Fig. 1d, the absorption bands at (*α*) 2105−2107 cm^−1^ and (*β*) 2094−2096 cm^−1^ are ascribed to CO bound to Cu^+^ and Cu^0^ species, respectively^22,31^, in which the variation of surface Cu^+^/Cu^0^ ratio (*α*/*β*) follows a similar tendency to that of *quasi-*in situ Cu LMM spectra.

The electronic structure and coordination state of copper species were studied in detail through X-ray absorption spectroscopy (XAS). As shown in the normalized Cu K-edge XANES spectra (Fig. 1e), the white line peaks of *y*Cu/Cu(Al)O_*x*_ and 4.20Cu/Al_2_O_3_ are located between Cu foil and Cu_2_O standard, and the latter sample gives the lowest oxidation state (rather close to Cu foil). For the *y*Cu/Cu(Al)O_*x*_ samples, the intensity of white line peaks decreases gradually from 0.95Cu/Cu(Al)O_*x*_ to 7.18Cu/Cu(Al)O_*x*_, indicative of a decline in average valence state of Cu species (Cu_AVS_). This is in accordance with the results from linear combination fitting (LCF) analysis (Supplementary Fig. 15), where the average valence state of Cu (Cu_AVS_) decreases gradually from +0.86 to +0.25 from 0.95Cu/Cu(Al)O_*x*_ to 7.18Cu/Cu(Al)O_*x*_ sample. In contrast, the control sample Cu/Al_2_O_3_ displays the lowest Cu_AVS_ (+0.12). The corresponding Cu K-edge extended X-ray absorption fine structure (EXAFS) spectra from Fourier transform are shown in Fig. 1f and Supplementary Fig. 16, where the peaks at 1.47 and 2.25 Å (no calibration) are assigned to Cu−O and Cu−Cu bonds from the first shell of Cu_2_O and Cu foil, respectively^26,28^. For the *y*Cu/Cu(Al)O_*x*_ samples, the Cu−O bond length is longer than that in Cu_2_O sample. Based on the fitting results and wavelet transform (Supplementary Fig. 17 and Supplementary Table 2), the longer bond length of Cu−O in *y*Cu/Cu(Al)O_*x*_ samples (1.84 ± 0.01 Å) relative to Cu_2_O standard (1.81 ± 0.01 Å) indicates a distorted tetrahedral structure due to the partial substitution of Cu by Al, which is possibly related to the formation of unique Cu−O−Al geometric coordination (CuAlO_2_-like structure). This is consistent with the DFT calculation results (Fig. 1g−i), in which the Cu^+^−O bond length in Cu(111)/CuAlO_2_(101) (1.90 Å) is significantly longer than that in Cu_2_O(111) (1.83 Å) and Cu(111)/Cu_2_O(111) (1.83 Å). In addition, the coordination number of Cu−Cu bond in *y*Cu/Cu(Al)O_*x*_ increases from 5.1 to 8.2 whilst that of Cu−O bond declines from 1.7 to 0.5 along with the increment of Cu/Al ratio (Fig. 1f, Supplementary Fig. 17 and Supplementary Table 2). We further correlated the coordination number of Cu−O and Cu−Cu bonds with the fraction of Cu_2_O/Cu in these samples. Based on the XRD Rietveld refinement, *quasi-*in situ Cu LMM, in situ CO-DRIFTS, XAFS-LCF, and EXAFS-Fit analysis results, a negative correlation between Cu^+^/Cu^0^ ratio and Cu/Al ratio is established, demonstrating the significant role of amorphous alumina in stabilizing Cu^+^ species (Supplementary Fig. 18).

In addition, we changed the calcination temperature of 4.25CuAlO_*x*_ precursor within 500−800 °C to regulate the doping degree of amorphous alumina on Cu_2_O. As shown in the H_2_-TPR curves (Supplementary Fig. 19a), the reduction peak moves towards higher temperature with the increase of precursor roasting temperature, signifying an enhanced Cu-Al_2_O_3_ interaction. After the subsequent hydrogen activation treatment, XRD patterns show that from 4.25Cu/Cu(Al)O_*x*_−500 to 4.25Cu/Cu(Al)O_*x*_−800 sample (Supplementary Fig. 19b, c), the Cu_2_O(111) reflection shifts to higher 2*θ* direction accompanied with an increased peak intensity, which indicates a decreased Cu_2_O cell volume due to the doping of Al atoms with smaller radius. Moreover, XPS (Supplementary Fig. 19d) and XANES spectra (Supplementary Fig. 19e) demonstrate a gradual increase of Cu^+^/Cu^0^ ratio from 4.25Cu/Cu(Al)O_*x*_−500 to 4.25Cu/Cu(Al)O_*x*_−800. Meanwhile, an enhanced proportion of Cu−O−Al geometric coordination is further verified through fitting the Cu−O and Cu−O−Al scattering paths based on Cu K-edge EXAFS spectra with Cu_2_O and CuAlO_2_ as standard samples (Supplementary Figs. 19f, 20 and Supplementary Table 3). The average bond length of Cu−O increases from 1.84 ± 0.01 (4.25Cu/Cu(Al)O_x_−500) to 1.89 ± 0.02 Å (4.25Cu/Cu(Al)O_*x*_−800) in sequence, accompanied with a gradual increase in the coordination number of Cu−Al bond from the second shell (bond length: ∼3.17 Å), which indicates the formation of Cu−O−Al geometric coordination. In addition, we built 7 modified Cu_2_O models in which Cu^+^ is substituted by Al^3+^ with 7 different replacement percentages (Supplementary Fig. 21a−n). The calculated average bond length of Cu−O enhances from 1.83 to 1.87 Å as the Al/Cu ratio increases from 0 to 7.7% (Supplementary Fig. 21o), in agreement with the experiment results.

**Fig. 2:**
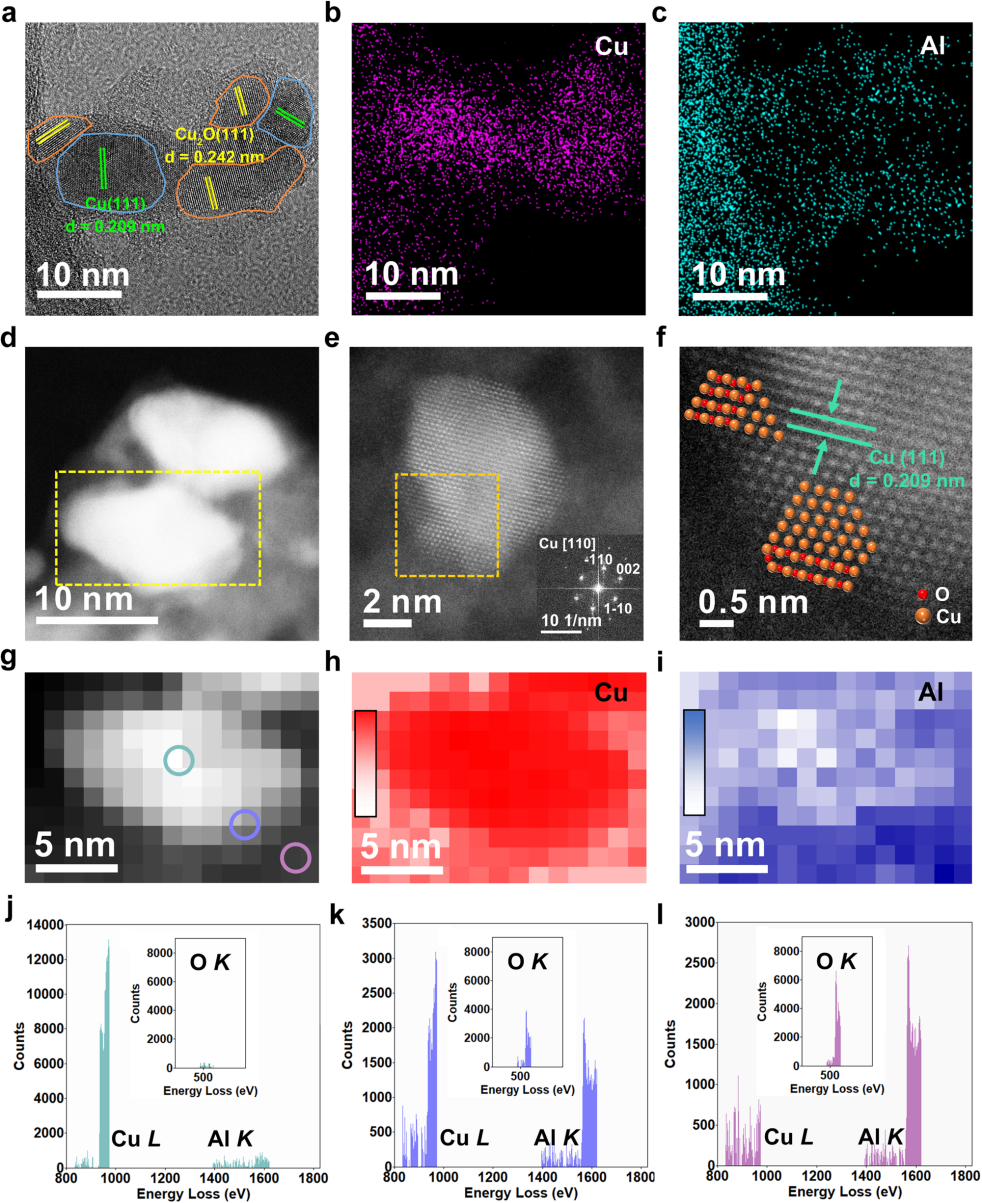
Microscopy characterizations of the 4.25Cu/Cu(Al)Ox sample. **a** High-magnification TEM, **b**, **c** EDS mapping images, **d**−**f** High-magnification STEM-HAADF images, **g** EELS mapping image of 4.25Cu/Cu(Al)O_*x*_ sample in yellow box in (**d**). Elemental areal density of **h** Cu and **i** Al in **g** (color bars from bottom to top indicates increased intensity). **j**−**l** EELS signals of Cu *L*, Al *K,* and O *K* at different tagged sites in (**g**).

TEM images of these *y*Cu/Cu(Al)O_*x*_ samples show a uniform dispersion of Cu nanoparticles on substrate (Supplementary Fig. 22); and the average particle size increases from 7.0 to 9.3 nm along with the increment of Cu/Al ratio from 0.95 to 7.18. As shown in the HR-TEM images (Fig. 2a and Supplementary Fig. 23), two clear crystalline phases are identified for these *y*Cu/Cu(Al)O_*x*_ samples: the lattice fringe of 0.209 nm corresponds to the Cu(111) plane and that of 0.242 nm around Cu species is due to the Cu_2_O(111) plane from face-centered cubic packing. Combining with the EDS mapping results (Fig. 2b, c and Supplementary Fig. 24), stabilized Cu or Cu_2_O phase on the Al_2_O_3_ support is verified. In contrast, the control sample 4.20Cu/Al_2_O_3_ shows an average Cu particle size of 7.1 nm (Supplementary Fig. 25a), with the presence of individual Cu(111) plane (Supplementary Fig. 25b). To clearly reveal the microstructure, STEM and corresponding EELS mapping for the typical sample 4.25Cu/Cu(Al)O_*x*_ were measured. As shown in STEM-HAADF (Fig. 2d−f and Supplementary Fig. 26) and STEM-BF (Supplementary Fig. 27) images, this sample is constituted by Cu nanoparticle and its surrounding O-terminal Cu species, in agreement with the XRD and XAFS results. Furthermore, three sites are selected (Fig. 2g) to carry out EELS element analysis for identifying the fine structure. At the surface site on copper particle (green tagged), Cu^0^ species displays overwhelming superiority with a very small amount of Al and O (Fig. 2j). At the boundary site between Cu particle and support (blue tagged), a simultaneous presence of Cu *L*, Al *K,* and O *K* is observed (Fig. 2k), corresponding to the Cu^+^ species stabilized by amorphous alumina. At the site away from Cu particle (pink tagged), the EELS signal of Cu shrinks accompanied with enhanced signals of Al and O (Fig. 2l). These results are in accordance with the areal density of Cu (Fig. 2h) and Al (Fig. 2i) in the 4.25Cu/Cu(Al)O_*x*_ sample, where the relative content of copper decreases while that of aluminum increases gradually from the Cu particle surface to the Cu−Al_2_O_3_ interface. As shown in STEM and EELS, the existence of Cu_2_O phase (Fig. 2d−l) is related to the stabilizing effect of amorphous alumina support on Cu^+^, which suppresses its further reduction. Thus, the 4.25Cu/Cu(Al)O_*x*_ sample is featured by aluminum-stabilized Cu^+^ adjacent to Cu^0^ nanoparticle immobilized on Al_2_O_3_ support, whose schematic structure diagram is shown in Supplementary Fig. 28 (Supplementary Note 1).

### Catalytic performance and kinetic studies

**Fig. 3:**
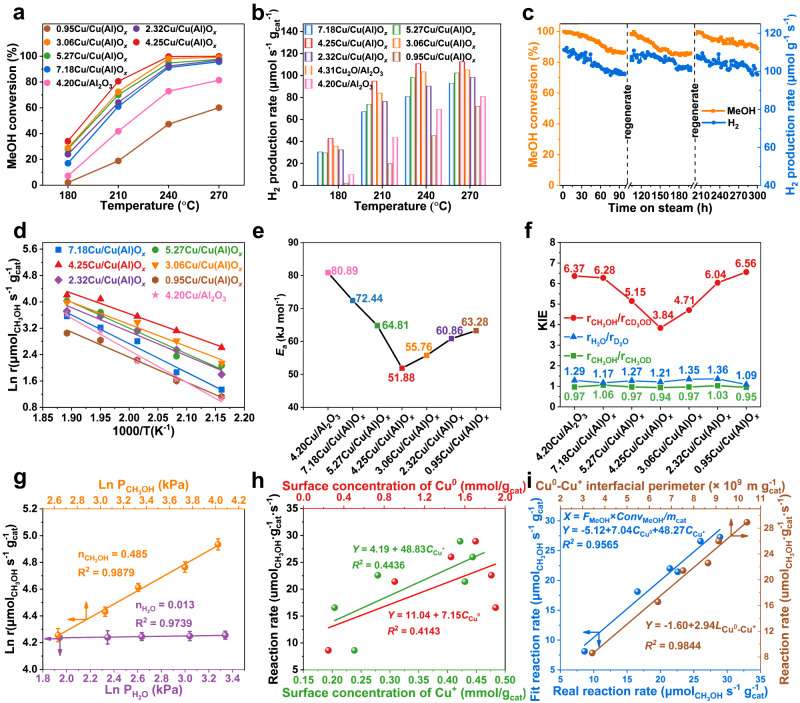
Catalytic performance towards MSR and kinetic studies on various samples. **a** Methanol conversion and **b** H_2_ production rate over various samples within 180−270 °C (Reaction conditions: catalyst (0.25 g) + SiO_2_ (2.50 g); liquid feed of S/C = 2 at 0.040 mL min^−1^; He carrier at 50.0 mL min^−1^; time on stream: 1.0 h). **c** Methanol conversion and H_2_ production rate vs. reaction time on stream of 4.25Cu/Cu(Al)O_*x*_ catalyst at 240 °C. **d** Kinetic studies on Arrhenius plots, **e** activation energy (*E*_a_) and **f** KIE values for MSR reaction over various samples (Reaction conditions: catalyst (0.01−0.10 g) + SiO_2_ (0.1−1.0 g); liquid feed of S/C = 2 at 0.040−0.080 mL min^−1^; He carrier at 50.0 mL min^−1^; time on stream: 0.5 h; methanol conversion less than 20%). **g** Reaction orders of CH_3_OH and H_2_O over 4.25Cu/Cu(Al)O_*x*_ catalyst at 240 °C (error bar comes from the uncertainty obtained from three parallel experiments). **h** Correlation between methanol reaction rate and surface concentration of individual Cu^0^ (*C*_Cu_0) or Cu^+^ (*C*_Cu_ + ). **i** Linear fitting results of methanol reaction rate as a function of both Cu^0^ (*C*_Cu_0) and Cu^+^ (*C*_Cu_ + ) as well as Cu^0^−Cu^+^ interfacial perimeter.

Catalytic performances of *y*Cu/Cu(Al)O_*x*_ samples were evaluated towards ESR reaction in a fix-bed reactor at 180−270 °C with a H_2_O/CH_3_OH molar ratio of 2 in feed. As an endothermic reaction, both CH_3_OH conversion and H_2_ production rate increase upon elevating reaction temperature (Fig. 3a, b); while CO_2_ selectivity maintains above 98% for all these samples (Supplementary Fig. 29). Interestingly, a volcanic tendency of catalytic activity is found from 0.95Cu/Cu(Al)O_*x*_ to 7.18Cu/Cu(Al)O_*x*_; and the 4.25Cu/Cu(Al)O_*x*_ catalyst exhibits the highest CH_3_OH conversion (99.5%) as well as H_2_ production rate (110.8 μmol s^−1^ g_cat_^−1^) at 240 °C, which is preponderant to the reported copper-based catalysts for MSR (Supplementary Table 4) and even exceeds most precious metal catalysts. In addition, the studies on reduction temperature from 170 to 300 °C (Supplementary Fig. 30) show that the 4.25Cu/Cu(Al)O_*x*_ sample reduced at 220 °C with an appropriate proportion of Cu^+^ species (Supplementary Fig. 31) displays the highest catalytic activity. Furthermore, the stability test of 4.25Cu/Cu(Al)O_*x*_ was also examined (Fig. 3c). Although the CH_3_OH conversion and H_2_ production rate show somewhat decrease after a 100 h time-on-stream test (from 99.5% and 110.8 μmol s^−1^ g_cat_^−1^ to 86.3% and 99.4 μmol s^−1^ g_cat_^−1^), the catalytic performance can recover to its original level after a regeneration process (air oxidation at 300 °C for 1 h followed by a reduction in 25% H_2_/N_2_ at 220 °C for 1 h). No significant change in catalyst structure, Cu particle size, and chemical valence is observed for the used catalyst after three-cycle tests (300 h), in comparison with the fresh one (Supplementary Figs. 32−34), which indicates that the decrease of catalytic activity is not associated with the variation in physicochemical properties. As proved by IR spectroscopy (Supplementary Fig. 35), the deactivation of 4.25Cu/Cu(Al)O_*x*_ catalyst primarily arises from carbon deposition (the band between 2800 and 3000 cm^−1^ is assigned to C−H species)^24^, and the carbonaceous species can be facilely removed via a regeneration process.

In addition, the activation energy (*E*_a_) and kinetic isotope effect (KIE) were then studied over these *y*Cu/Cu(Al)O_*x*_ samples under dynamics test conditions. According to the Arrhenius equation (Fig. 3d), the control sample 4.20Cu/Al_2_O_3_ gives the largest activation energy of 80.89 kJ mol^−1^. In the cases of *y*Cu/Cu(Al)O_*x*_ samples, the activation energy declines first and then increases from 0.95Cu/Cu(Al)O_*x*_ to 7.18Cu/Cu(Al)O_*x*_, and the lowest value of 51.88 kJ mol^−1^ is present in the 4.25Cu/Cu(Al)O_*x*_ catalyst (Fig. 3e), in accordance with the highest catalytic activity. Similar volcanic curves were obtained by correlating the methanol reaction rate with the surface Cu^+^/Cu^0^ ratio (Supplementary Fig. 36). To acquire clear-cut kinetics information, the KIE values of D_2_O, CH_3_OD, and CD_3_OD were further tested (Fig. 3f). The k_H_/k_D_ values of D_2_O and CH_3_OD are estimated to be 1.09−1.36 and 0.95−1.06, respectively; in contrast, the k_H_/k_D_ value of CD_3_OD is located between 3.84 and 6.56, several times larger than the former two cases, indicating that the breakage of C−H bonds is the rate-determining step during the MSR reaction. Importantly, a similar volcanic-type profile for the catalytic activity as a function of k_H_/k_D_ value of CD_3_OD is found from 0.95Cu/Cu(Al)O_*x*_ to 7.18Cu/Cu(Al)O_*x*_ (red line); whilst the KIE values of CH_3_OD (green line) and D_2_O (blue line) give no significant difference on these samples. In addition, we measured the reaction order of CH_3_OH and H_2_O over the 4.25Cu/Cu(Al)O_*x*_ catalyst (Fig. 3g). The reaction rate of methanol displays a positive relationship with the CH_3_OH partial pressure, but does not show obvious correlation with H_2_O partial pressure. Through data fitting, the reaction orders of CH_3_OH and H_2_O are determined to be 0.485 and 0.013, respectively, which indicates that the cleavage of C−H bond in methanol is crucial whilst the H_2_O activation is not involved in the rate-determining step of MSR reaction. The results above demonstrate that the 4.25Cu/Cu(Al)O_*x*_ catalyst promotes the breakage of C−H bond, which is responsible for its excellent catalytic activity.

Based on the structural characterizations and catalytic evaluations, the Cu^0^−Cu^+^ synergistic catalysis in *y*Cu/Cu(Al)O_*x*_ samples would play a key role. Thus, we quantified the surface concentration of Cu^0^ (*C*_Cu_0) and Cu^+^ (${{{{\rm{C}}}}}_{{{{{{\rm{Cu}}}}}}^{+}}$) species as well as interfacial perimeters of Cu^0^−Cu^+^ (${L}_{{{{{{\rm{Cu}}}}}}^{0}-{{{{{\rm{Cu}}}}}}^{+}}$) of all these samples via N_2_O titration (Supplementary Fig. 37) and CO-TPD (Supplementary Fig. 38). No obvious correlation between normalized CH_3_OH reaction rate and individual *C*_Cu_0 or ${{{{\rm{C}}}}}_{{{{{{\rm{Cu}}}}}}^{+}}$ is found (Fig. 3h). Interestingly, if we correlate the CH_3_OH reaction rate with *C*_Cu_0 and ${{{{\rm{C}}}}}_{{{{{{\rm{Cu}}}}}}^{+}}$ simultaneously, a binary function relation of rate equation is obtained (Fig. 3i): *Y* = −5.12 + 7.04 *C*_Cu_0 + 48.27 ${{{{\rm{C}}}}}_{{{{{{\rm{Cu}}}}}}^{+}}$. Furthermore, a linear relationship between methanol reaction rate and ${L}_{{{{\mbox{Cu}}}}^{0}-{{{\mbox{Cu}}}}^{+}}$ is obtained (Fig. 3i). It is thus concluded that catalytic activity depends on the synergistic catalysis of Cu^0^ and Cu^+^ rather than a single active site, and the Cu^0^−Cu^+^ interfacial sites are imperative for boosting the rate-determining step in MSR reaction (C−H bond cleavage).

### Catalytic reaction mechanism of MSR

For the MSR reaction, three possible pathways have been discussed in the previous studies: (1) CH_3_OH decomposition to CO and H_2_, followed by CO steam reforming to produce CO_2_ and H_2_ (denoted as CO* route)^4–6,32^; (2) one step oxidization of CH_3_OH to HCOO* by hydroxyl group or reactive oxygen species from H_2_O dissociation, followed by HCOO* decomposition to produce CO_2_ and H_2_ (denoted as HCOO* route)^7,33–35^; (3) CH_3_OH dehydrogenation to HCOOCH_3_, which is then hydrolyzed to HCOO*, followed by further decomposition to produce CO_2_ and H_2_ (denoted as HCOOCH_3_* route)^9,10,36,37^. Nonetheless, owing to the complexity of MSR reaction and the diversity of catalyst structure, studies on catalytic reaction pathways over copper catalysts are controversial.

**Fig. 4:**
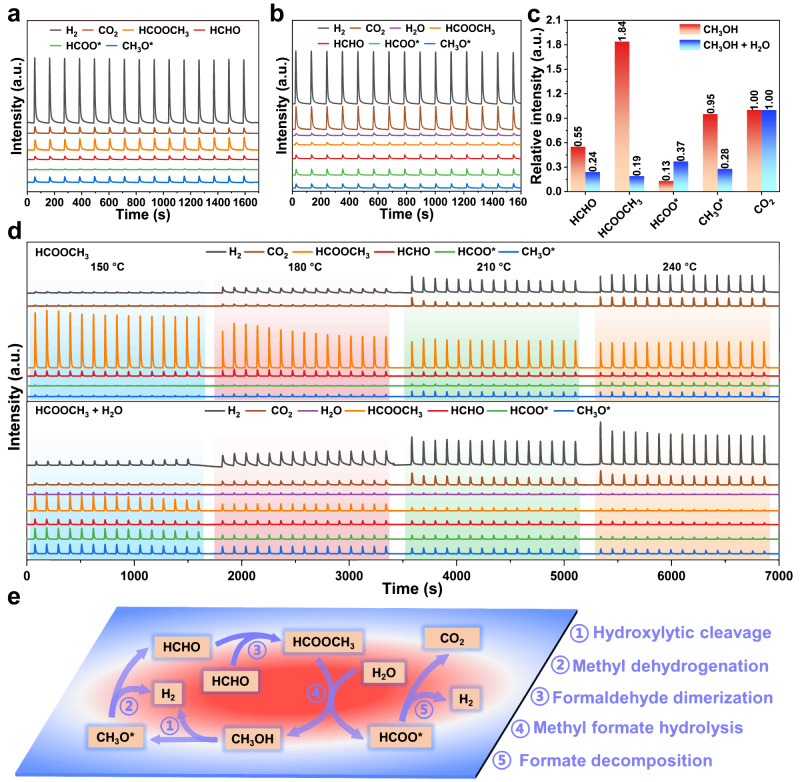
Operando pulse experiments and reaction mechanism of MSR. MS signals for the pulse experiments of **a** methanol and **b** methanol-water (1:2) over 4.25Cu/Cu(Al)O_*x*_ at 240 °C, respectively. **c** Relative intensity of the reaction intermediates normalized by CO_2_ signal based on the results of (**a**) and (**b**). **d** MS signals for the pulse experiments of methyl formate and methyl formate-water (1:2) over 4.25Cu/Cu(Al)O_*x*_ from 150 to 240 °C, respectively. **e** Schematic illustration for MSR reaction mechanism (HCOOCH_3_* route).

To elucidate the MSR reaction route over *y*Cu/Cu(Al)O_*x*_ catalysts in this work, the *operando* pulse experiments equipped with mass spectrometer detector were carried out (Fig. 4 and Supplementary Fig. 39). As shown in Fig. 4a, the signals of reaction intermediates (CH_3_O*, HCHO, HCOOCH_3_ and HCOO*) and reaction products (H_2_ and CO_2_) are captured after the introduction of CH_3_OH/He on 4.25Cu/Cu(Al)O_*x*_ at 240 °C (Fig. 4c, red). In contrast, when CH_3_OH + H_2_O is co-introduced (Fig. 4b), the relative intensity of HCOOCH_3_ decreases accompanied with the increase of HCOO* signal (Fig. 4c, blue). This indicates that the co-introduction of H_2_O greatly promotes the hydrolysis of HCOOCH_3_. The same results are also found on 0.95Cu/Cu(Al)O_*x*_ and 7.18Cu/Cu(Al)O_*x*_ samples, but the higher relative intensities of HCOO* and CH_3_O* suggest a lower reaction rate (Supplementary Fig. 39).

Furthermore, we performed the pulse experiments of HCOOCH_3_ and HCOOCH_3_ + H_2_O over 4.25Cu/Cu(Al)O_*x*_ catalyst at different reaction temperatures (Fig. 4d). In the former case, as the temperature increases from 150 to 240 °C, the signals of HCOOCH_3_ decline accompanied with the rise of H_2_, CO_2_, CH_3_O* and HCOO* signals due to the dissociation of HCOOCH_3_. In the latter case, the presence of H_2_O promotes the conversion of HCOOCH_3_, with significantly weakened HCOOCH_3_ signal but enhanced CH_3_O* and HCOO* signals. Therefore, the whole MSR reaction mechanism over 4.25Cu/Cu(Al)O_*x*_ catalyst is proposed in Fig. 4e: CH_3_OH firstly undergoes dehydrogenation to form CH_3_O* and HCHO species; then HCHO experiences dimerization or reacts with CH_3_O* to generate HCOOCH_3_; subsequently, HCOOCH_3_ hydrolyzes to form HCOOH and CH_3_O*, and CH_3_O* re-participates in the catalytic cycle; finally, the decomposition of HCOOH occurs to produce CO_2_ and H_2_. In addition, according to the MS spectra results (Fig. 4c, Supplementary Fig. 39, and Supplementary Note 2), the signals of CH_3_O* and HCOO* are much stronger than those of HCHO and HCOOCH_3_ during the MSR reaction, indicating that the conversion of CH_3_O* and HCOO* is a kinetically slower process. According to the kinetic studies (Fig. 3f, g), the fracture of C−H bonds in CH_3_O* and HCOO* intermediates is proved as the rate-determining step; moreover, water molecule promotes the decomposition of HCOOCH_3_ but does not participate directly in the cleavage of C−H bonds.

**Fig. 5:**
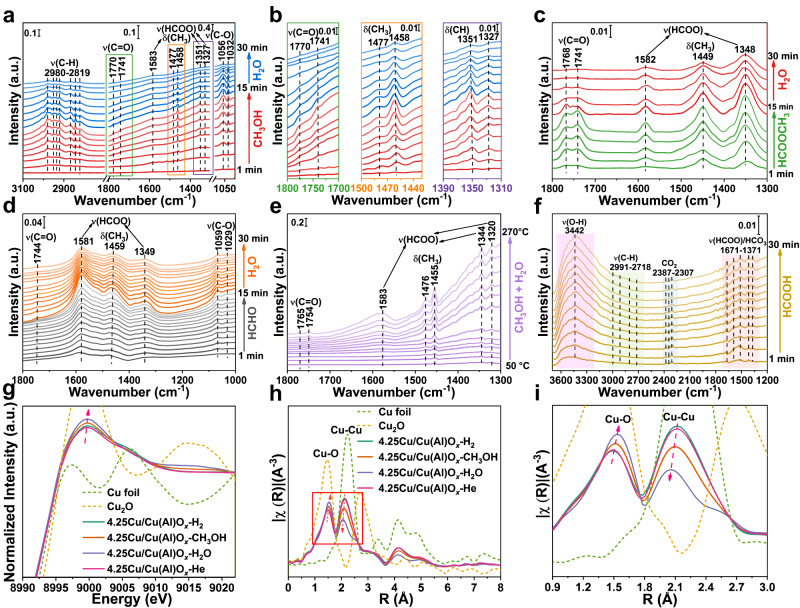
In situ studies on active sites identification. In situ FT-IR spectra of 4.25Cu/Cu(Al)O_*x*_ along with the sequential introduction of **a**, **b** CH_3_OH/He (1−15 min) and H_2_O/He (15−30 min), **c** HCOOCH_3_/He (1−15 min) and H_2_O/He (15−30 min), **d** HCHO/He (1−15 min) and H_2_O/He (15−30 min) at 240 °C, **e** CH_3_OH/H_2_O/He from 50 to 270 °C, **f** HCOOH/He (1−30 min) at 180 °C. *Operando* XAFS spectra of Cu k-edge at **g** E-space and **h**, **i** R-space for pristine 4.25Cu/Cu(Al)O_*x*_ catalyst (reduction in H_2_, CH_3_OH pumping, H_2_O pumping and He purging in turn).

In situ FT-IR measurements were carried out to study the microscopic reaction mechanism of MSR. As shown in Fig. 5a, b, when introducing CH_3_OH to the 4.25Cu/Cu(Al)O_*x*_ catalyst at 240 °C (red lines), signals including dissociative adsorption of CH_3_OH (1032 and 1056 cm^−1^), bending vibration of HCOO* (1327, 1351 and 1583 cm^−1^) and CH_3_O* species (1458 and 1477 cm^−1^), stretching vibration of C−H bond (2800−3000 cm^−1^) and very weak C=O bond attributed to HCHO or HCOOCH_3_ species (1741 and 1770 cm^−1^) are observed^7,8,24,26,36,38^. With the increase of ventilation time, the peak strength of these species enhances gradually, indicating that CH_3_OH molecule undergoes activation and dehydrogenation on the catalyst surface. Subsequently, switching CH_3_OH/He to H_2_O/He (blue lines in Fig. 5a, b) results in the decline of these bands, demonstrating the transformation of these reaction intermediates after the introduction of H_2_O. Furthermore, when exposing 4.25Cu/Cu(Al)O_*x*_ catalyst to CH_3_OH/H_2_O/He from 50 to 270 °C (Fig. 5e), the signals of *δ*_C−H_, *ν*_HCOO_ and *ν*_C=O_ increase gradually, accompanied with the appearance of gas CO_2_ (2380−2307 cm^−1^)^12,26,32^ and OH^−^ group (3390 and 3723 cm^−1^) (Supplementary Fig. 40), in accordance with the pulse experiments results (Fig. 4b).

Subsequently, we carried out in situ FT-IR to study the adsorption and reaction behavior of HCOOCH_3_ intermediate at 240 °C on the 4.25Cu/Cu(Al)O_*x*_ catalyst (Fig. 5c, green lines). After the introduction of HCOOCH_3_, the bands attributed to C=O bond (1741 and 1768 cm^−1^) in HCOOCH_3_ and the ones assigned to C−H and COO^−^ group from adsorbed HCOOCH_3_ (1348, 1449 and 1582 cm^−1^) are observed. After switching to a saturated water vapor (Fig. 5c, red lines), the C=O bond disappears accompanied with the gradual decline of HCOO* and CH_3_O* species, corresponding to the HCOOCH_3_ hydrolysis and C−H bond cleavage in reaction intermediates. In the case of in situ FT-IR measurement on the adsorption and reaction process of HCHO intermediate on 4.25Cu/Cu(Al)O_*x*_ catalyst (Fig. 5d, gray lines), the intense absorption bands of HCOO* and CH_3_O* (1459, 1349 and 1581 cm^−1^) are detected, accompanied with the appearance of a weak C=O bond (1742 cm^−1^), which is similar as the FT-IR spectra of adsorbed CH_3_OH and HCOOCH_3_ (Fig. 5a−c). Afterwards, a switching to saturated water vapor induces the decline of HCOO* and CH_3_O* signals (Fig. 5d, orange lines). The results above verify that the HCOOCH_3_ intermediate is derived from the further transformation of formaldehyde, and water promotes the hydrolysis and transformation of methyl formate, which is consistent with the pulse experiment results (Fig. 4d).

To elucidate the Cu^0^−Cu^+^ synergistic catalytic mechanism, the CH_3_OH conversion over 0.95Cu/Cu(Al)O_*x*_ and 7.18Cu/Cu(Al)O_*x*_ samples with the highest proportion of Cu^+^ and Cu^0^ respectively, was detected through in situ FT-IR (Supplementary Figs. 41, 42) combined with pulse experimental analysis (Supplementary Fig. 39). In comparison with the 4.25Cu/Cu(Al)O_*x*_ catalyst, much stronger signals of CH_3_O* and HCOO* are observed in the presence of 0.95Cu/Cu(Al)O_*x*_ or 7.18Cu/Cu(Al)O_*x*_, indicating a slower conversion rate. Moreover, relative to 4.25Cu/Cu(Al)O_*x*_ (Fig. 5b), the IR absorption frequencies of CH_3_O* and HCOO* intermediates show a blue-shift (move towards high wavenumber) and red-shift (move towards low wavenumber) over 0.95Cu/Cu(Al)O_*x*_ (Supplementary Fig. 41b) and 7.18Cu/Cu(Al)O_*x*_ (Supplementary Fig. 42b), respectively. In addition, in situ FT-IR measurements for CD_3_OD conversion were also performed to study the isotope effects of C−D bonds cleavage in CD_3_O* and DCOO* intermediates over the three catalysts (Supplementary Figs. 43−45). After switching to a saturated H_2_O vapor, the consumption rates of CH_3_O* (${{{{{{\rm{R}}}}}}}_{{{{{{{\rm{H}}}}}}}_{{{{{{\rm{m}}}}}}}}$) and HCOO* species (${{{{{{\rm{R}}}}}}}_{{{{{{{\rm{H}}}}}}}_{{{{{{\rm{f}}}}}}}}$) relative to CD_3_O* (${{{{{{\rm{R}}}}}}}_{{{{{{{\rm{D}}}}}}}_{{{{{{\rm{m}}}}}}}}$) and DCOO* species (${{{{{{\rm{R}}}}}}}_{{{{{{{\rm{D}}}}}}}_{{{{{{\rm{f}}}}}}}}$) were calculated, where the ${{{{{{\rm{R}}}}}}}_{{{{{{{\rm{H}}}}}}}_{{{{{{\rm{m}}}}}}}}$, ${{{{{{\rm{R}}}}}}}_{{{{{{{\rm{H}}}}}}}_{{{{{{\rm{f}}}}}}}}$, ${{{{{{\rm{R}}}}}}}_{{{{{{{\rm{D}}}}}}}_{{{{{{\rm{m}}}}}}}}$ and ${{{{{{\rm{R}}}}}}}_{{{{{{{\rm{D}}}}}}}_{{{{{{\rm{f}}}}}}}}$ were the absolute value of slope from linear fitting (Supplementary Fig. 46 and Supplementary Note 3). The slight isotopic effect indicates that the C−H bonds breakage is significantly promoted on the surface of 4.25Cu/Cu(Al)O_*x*_ catalyst. The favorable transformation of HCOO* over 4.25Cu/Cu(Al)O_*x*_ (Fig. 5f) is also demonstrated based on in situ FT-IR spectra of HCOOH adsorption: the C−H stretching vibration bands within 2700−3000 cm^−1^ are inconspicuous in comparison with the 0.95Cu/Cu(Al)O_*x*_ (Supplementary Fig. 47) and 7.18Cu/Cu(Al)O_*x*_ samples (Supplementary Fig. 48). In addition, H_2_/D_2_ exchange (Supplementary Fig. 49) and H_2_-TPD measurements (Supplementary Fig. 50 and Supplementary Note 4) demonstrate that the detachment of H from the catalyst surface is facile, where the Cu^0^ site with a strong dehydrogenation capacity is responsible for the extraction of atomic H and desorption of H_2_.

*Operando* XAFS measurements were carried out to reveal the dynamic evolution in electronic structure and coordination state of Cu^0^−Cu^+^ synergistic sites during the MSR reaction. As shown in Fig. 5g, compared with the pristine 4.25Cu/Cu(Al)O_*x*_ sample (green line), the intensity of the white line peak increases along with the sequential introduction of CH_3_OH (orange line) and H_2_O (blue line), indicating that the Cu species undergoes an electronic reconfiguration due to the electron transfer from catalyst to reactive species. Correspondingly, both the coordination number and bond length of Cu−Cu decrease after the introduction of CH_3_OH and H_2_O, as demonstrated by the EXAFS spectra (Fig. 5h, i) and fitting results in R-space of Cu k-edge (Supplementary Figs. 51, 52 and Supplementary Table 5). In contrast, the coordination number and bond length of Cu−O increase simultaneously, owing to the formation of additional Cu^0^−H and Cu^+^−O bonds. After He purging (pink line) for 15 min to remove the surface adsorbates, both the XANES and EXAFS spectra of 4.25Cu/Cu(Al)O_*x*_ restore to their initial states. The results substantiate that the Cu^0^−Cu^+^ interfacial sites participate in the substrate activation and C−H bonds cleavage. When *operando* XAFS measurements is performed by introducing HCHO (Supplementary Fig. 53), HCOOCH_3_ (Supplementary Fig. 54) or HCOOH (Supplementary Fig. 55), a similar change is also observed, verifying a dynamic reconstruction process of the Cu^0^−Cu^+^ interfacial sites during the whole MSR reaction. As a comparison, the 7.18Cu/Cu(Al)O_*x*_ sample does not show obvious variation in electronic and geometric structure (Supplementary Figs. 56, 57 and Supplementary Table 6), since a rather low ratio of Cu^+^/Cu^0^ is not conducive to C**−**H bonds cleavage.

**Fig. 6:**
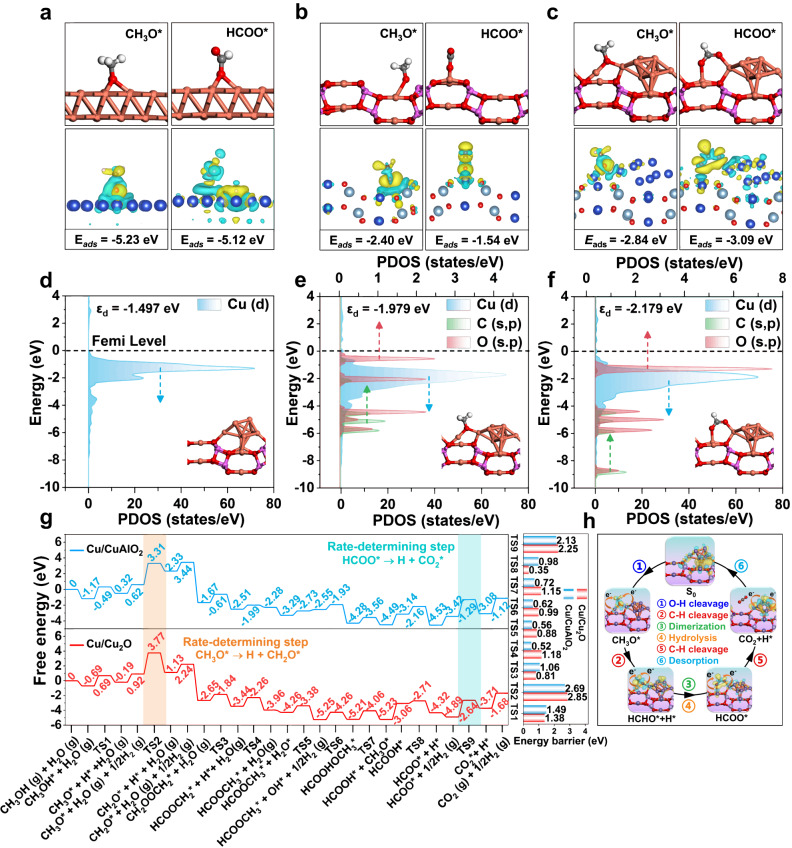
DFT calculations on catalytic active sites towards MSR. **a**−**c** Optimized adsorption configurations of CH_3_O* and HCOO* accompanied with the charge density difference (CDD) on the **a** Cu(111), **b** CuAlO_2_(101) and **c** Cu(111)/CuAlO_2_(101) models, respectively (white, gray, red, orange and purple balls indicate H, C, O, Cu and Al atoms, respectively). **d**−**f** Projected density of states (PDOS) for the pristine **d** Cu/CuAlO_2_, and adsorbed **e** CH_3_O* and **f** HCOO* intermediates at the Cu/CuAlO_2_ interface. **g** Full potential reaction pathway of MSR reaction following the HCOOCH_3_* mechanism over Cu/CuAlO_2_ and Cu/Cu_2_O, respectively. ‘TS’ denotes the transition state. Numbers located at the horizontal line represent free energy of corresponding intermediate (the inset gives the energy barrier of each transient state). **h** Schematic illustration for the dynamic reconstruction process of Cu^0^−Cu^+^ interfacial sites towards C−H bonds cleavage of CH_3_O* and HCOO*.

Furthermore, the full-path potential energy barriers for MSR were studied by DFT calculations (Fig. 6 and supplementary Figs. 58−100). Given the results in XRD patterns and the stabilizing effect of amorphous alumina on Cu^+^, the optimized Cu(111)/Cu_2_O(111) and Cu(111)/CuAlO_2_(101) structures after thermodynamic analysis are used to model the Cu^0^−Cu^+^ interfacial sites (Supplementary Figs. 59 and 60). The calculated projected density of states (PDOS) (Supplementary Fig. 61 and Supplementary Note 5) confirm that the Cu(111)/CuAlO_2_(101) structure induces a remarkable electron coupling, whose *d*-band center (*ε*_d_ = −1.497 eV) is located between Cu(111) (*ε*_d_ = −0.212 eV) and CuAlO_2_(101) (*ε*_d_ = −1.618 eV). A similar result is obtained in the Cu(111)/Cu_2_O(111) model, corresponding to the formation of Cu^0^−Cu^+^ interfacial sites, where the *d*-band center (*ε*_d_ = −1.622 eV) of Cu(111)/Cu_2_O(111) is located between Cu(111) (*ε*_d_ = −0.212 eV) and Cu_2_O(111) (*ε*_d_ = −1.722 eV).

The Gibbs free energy diagrams and detailed schematic representation of the stepwise reaction for HCOOCH_3_* route on Cu/Cu_2_O and Cu/CuAlO_2_ are displayed in Fig. 6g. Distinctly, the activation barriers for CH_3_O* formation (1.49 eV on Cu/CuAlO_2_ and 1.38 eV on Cu/Cu_2_O: CH_3_OH* → CH_3_O* + H*) (Supplementary Figs. 62 and 77), H_2_O dissociation (0.56 eV on Cu/CuAlO_2_ and 0.88 eV on Cu/Cu_2_O: H_2_O* → OH* + H*) (Supplementary Figs. 64, 68, 79, and 83), HCOOCH_3_* formation (1.06 eV on Cu/CuAlO_2_ and 1.18 eV on Cu/Cu_2_O: 2CH_2_O* → HCOOCH_3_*) (Supplementary Figs. 66, 67, 81, 82 and Supplementary Note 6) and HCOOCH_3_* hydrolysis (0.72 eV on Cu/CuAlO_2_ and 1.15 eV on Cu/Cu_2_O: HCOOCH_3_* + OH* → HCOOH* + CH_3_O*) (Supplementary Figs. 69, 70, 84, and 85) are lower than the dehydrogenation reactions of CH_3_O* (2.69 eV on Cu/CuAlO_2_ and 2.85 eV on Cu/Cu_2_O: CH_3_O* → CH_2_O*) (Supplementary Figs. 63 and 78) and HCOO* (2.13 eV on Cu/CuAlO_2_ and 2.25 eV on Cu/Cu_2_O: HCOO* → CO_2_* + H*) (Supplementary Figs. 72 and 87), indicating that the cleavage of C−H bonds in CH_3_O* and HCOO* acts as the rate-determining step. This is consistent with the experimental results. In addition, we also studied the formaldehyde oxidation route (Supplementary Figs. 73−76) and HCOOCH_3_* route (Fig. 6g and Supplementary Figs. 62−72) on the Cu/CuAlO_2_ model to reveal the role of H_2_O in the MSR reaction. Notably, for the HCOOCH_3_* pathway, H_2_O participates in the hydrolysis of methyl formate to form CH_3_O* and HCOO* (Supplementary Figs. 68−70) with a reactively low energy barrier (0.72 eV). In contrast, for the formaldehyde oxidation pathway (Supplementary Figs. 73−75), the hydroxyl species from H_2_O dissociation oxidizes CH_2_O to CH_2_OOH, followed by CH_2_OOH dehydrogenation to generate HCOO with a high energy barrier of 4.20 eV. Thus, the methyl formate pathway is favorable on surface of Cu/CuAlO_2_, and the role of water molecule is to hydrolyze methyl formate to produce formate, which is in accordance with the experimental results.

To in-depth study the Cu^0^−Cu^+^ synergistic catalysis mechanism, we further compared the reaction characteristics of rate-determining step (CH_3_O* → CH_2_O* and HCOO* → CO_2_) in Cu^0^ (Cu(111)), Cu^+^ (Cu_2_O and CuAlO_2_) and Cu^0^−Cu^+^ model (Cu/Cu_2_O and Cu/CuAlO_2_), respectively. The optimal adsorption configuration and charge density difference (CDD) (Fig. 6a−c, Supplementary Figs. 88, 89 and Supplementary Note 7) of CH_3_O* and HCOO* are calculated, whose adsorption energies give the following order: Cu(111) (−5.23 and −5.12 eV) > Cu/Cu_2_O (−3.37 and −4.47 eV) > Cu/CuAlO_2_ (−2.84 and −3.09 eV) > Cu_2_O (−2.41 and −2.39 eV) > CuAlO_2_ (−2.40 and −1.54 eV). In the case of Cu(111)/CuAlO_2_(101) system (Fig. 6c), the oxygen atom in CH_3_O* is co-adsorbed at the Cu^0^−Cu^+^ interfacial sites; whilst for HCOO*, the two oxygen atoms are adsorbed at interfacial Cu^0^ and Cu^+^ sites, respectively. These unique oxygen-end bridge adsorption configurations at the Cu^0^−Cu^+^ interfacial sites of Cu/CuAlO_2_ confer a moderate adsorption strength of both intermediates, in agreement with the in situ FT-IR results.

Compared with the Cu(111) (Cu^0^ site), CuAlO_2_(101) (Cu^+^ site) and Cu_2_O(111) (Cu^+^ site), the dehydrogenation processes (CH_3_O* → CH_2_O* and HCOO* → CO_2_) involved in the rate-determining step are greatly boosted at the Cu^0^−Cu^+^ interfacial sites in the cases of Cu(111)/Cu_2_O(111) and Cu(111)/CuAlO_2_(101), especially for the latter system (Supplementary Fig. 96). As shown in Supplementary Figs. 63, 78, 90, 92, and 94, the energy barrier of CH_3_O* dehydrogenation follows the sequence: CuAlO_2_ (4.07 eV) > Cu (3.59 eV) > Cu_2_O (3.06 eV) > Cu/Cu_2_O (2.85 eV) > Cu/CuAlO_2_ (2.69 eV). As shown in Supplementary Figs. 72, 87, 91, 93, and 95, the energy barrier of HCOO* dehydrogenation gives the following order: CuAlO_2_ (3.61 eV) > Cu (2.92 eV) > Cu_2_O (2.74 eV) > Cu/Cu_2_O (2.25 eV) > Cu/CuAlO_2_ (2.13 eV). The DFT calculation results support the experimental observations, in which the Cu^0^−Cu^+^ interfacial sites as intrinsic active centers facilitate the activation of reaction intermediates and promote the extraction of hydrogen, accounting for the extraordinarily high catalytic activity of 4.25Cu/Cu(Al)O_*x*_.

Furthermore, in terms of electronic structure, the adsorption of reaction intermediates (CH_3_O* and HCOO*) on the surface of Cu/CuAlO_2_ induces the decrease of *d*-band center of Cu, accompanied with the occupied orbital energy moving away from the Fermi level (Fig. 6d−f), which indicates electron transfer from *d*-states of Cu species to CH_3_O* and HCOO*. According to the Bader charge analysis (Supplementary Figs. 97 and 98), the Cu^0^−Cu^+^ interfacial sites in Cu(111)/CuAlO_2_(101) with oxyphilic ability result in the charge transfer from the catalyst interface to the adsorbed reaction intermediates. In terms of geometric structure, the bond length of Cu−O and Cu−Cu at the Cu(111)/CuAlO_2_(101) interface tends to elongate and shorten respectively during the process of C−H bonds activation (Supplementary Figs. 99 and 100), in good accordance with the reconfiguration phenomena obtained from in situ XAFS spectra. Thus, the dynamic evolution of electronic and geometric structure of Cu^0^−Cu^+^ interfacial sites towards C−H bonds cleavage is clearly revealed (Fig. 6h).

## Discussion

In summary, we report a *y*Cu/Cu(Al)O_*x*_ catalyst with well-defined and tunable Cu^0^−Cu^+^ interfacial sites applied to MSR reaction. The optimal catalytic performance is obtained on the 4.25Cu/Cu(Al)O_*x*_ catalyst with an appropriate Cu^0^−Cu^+^ interfacial sites, with a methanol conversion above 99%, a H_2_ production rate of 110.8 μmol s^−1^ g_cat_^−1^ and a satisfactory stability at 240 °C within 300 h. The MSR reaction over 4.25Cu/Cu(Al)O_*x*_ catalyst follows the HCOOCH_3_* route, and the Cu^0^−Cu^+^ interfacial synergistic catalysis plays a decisive role. The oxygen-containing intermediates (CH_3_O* and HCOO*) undergo activation adsorption at the Cu^0^−Cu^+^ interfacial sites with a moderate strength, giving rise to a reconstruction of catalyst interface as well as electron transfer from catalyst interface to reaction intermediates. The variations in both geometric and electronic structure result in a decreased energy barrier of C−H bonds fracture (the rate-determining step). This work provides atomic-level insights into Cu^0^−Cu^+^ interfacial synergistic catalysis in MSR, which can be extended to other heterogeneous catalytic systems towards rational design of high-performance catalysts.

## Methods

### Chemicals and materials

Cu(NO_3_)_2_·3H_2_O, Al(NO_3_)_3_·9H_2_O, *γ*-Al_2_O_3_, NaOH, Na_2_CO_3_ and CH_3_OH were obtained from the Aladdin chemical reagent company. Methanol-D4 (CD_3_OD), Deuteromethanol-D (CH_3_OD) and Deuterium oxide (D_2_O) were purchased from Adamas-Beta chemical reagent company. Quartz sand (SiO_2_, 40−60 mesh) was purchased from Tianjin Guangfu Fine Chemical Research Institute. Deionized (DI) water (resistivity: 18.2 MΩ·cm) was used in all experimental processes. All reagents were analytical grade and used without further purification.

### Synthesis of catalysts

The *y*Cu/Cu(Al)O_*x*_ catalysts were prepared via a co-precipitation method followed by the subsequent roasting and reduction processes. Typically, Cu(NO_3_)_2_·3H_2_O (4.832 g) and Al(NO_3_)_3_·9H_2_O (1.876 g) were dissolved in 50 mL of DI water (Solution A); NaOH (3.200 g) with 1.6 times equivalent concentration of metal ion (total of Cu^2+^ and Al^3+^) and Na_2_CO_3_ (2.120 g) with 2.0 times equivalent concentration of trivalent metal ion (Al^3+^) were dissolved in 100 mL of DI water (Solution B). With vigorous stirring, Solution A and B were dropwise added into a beaker with water (30 mL) maintaining a stable pH (9.3−9.4). The obtained slurry was transferred to an oil bath and aged at 95 °C for 8 h. The resulting precipitate was filtered, washed thoroughly and dried at 80 °C for 12 h, followed by a calcination at 500 °C in air for 4 h, to obtain the 4.25CuAlO_*x*_ catalyst. Other *y*CuAlO_*x*_ precursor samples (*y* = 0.95, 2.32, 3.06, 4.25, 5.27, 7.18, respectively, representing total molar ratio of Cu/Al from ICP-AES; *x* denotes the amount of oxygen coordinated with Cu and Al) were synthesized via the similar method described above, except changing the feeding amount of Cu(NO_3_)_2_·3H_2_O, Al(NO_3_)_3_·9H_2_O, NaOH and Na_2_CO_3_. Prior to use, the *y*CuAlO_*x*_ samples were activated in a mixture gas (25% H_2_/N_2_, 50 mL min^−1^) at 220 °C for 2 h to obtain the final catalysts, which were denoted as *y*Cu/Cu(Al)O_*x*_ (y = 0.95, 2.32, 3.06, 4.25, 5.27, 7.18). The 4.25Cu/Cu(Al)O_*x*_−250 and 4.25Cu/Cu(Al)O_*x*_−300 were obtained via reducing 4.25CuAlO_*x*_ precursor at 250 and 300 °C, respectively. The 4.25Cu/Cu(Al)O_*x*_−600, 4.25Cu/Cu(Al)O_*x*_−700 and 4.25Cu/Cu(Al)O_*x*_−800 catalysts were obtained through firstly roasting the 4.25CuAlO_*x*_ precursor at 600, 700 and 800 °C for 4 h, followed by activation in a mixture gas (25% H_2_/N_2_, 50 mL min^−1^) at 220 °C for 2 h.

The control sample 4.20CuO/Al_2_O_3_ was prepared through a wet impregnation method. Cu(NO_3_)_2_·3H_2_O (7.55 g) was dispersed in an aqueous mixture containing *γ*-Al_2_O_3_ powder (4.00 g), followed by sonication for 1 h to obtain a homogeneous suspension. After aging at room temperature for 6 h followed by drying the solvent at 80 °C, the resulting precipitate was calcinated at 500 °C in air for 4 h to obtain the catalyst precursor. Finally, the precursor was activated in a mixture gas (H_2_/N_2_ = 1:3, 50 mL min^−1^) at 220 °C for 2 h to obtain the 4.20Cu/Al_2_O_3_ sample.

### Catalytic evaluations

Catalytic performance towards MSR was evaluated in a fix-bed reactor equipped with a stainless-steel tube (interior diameter: 10 mm). In a catalytic process, 250 mg of catalyst precursor (*y*CuAlO_*x*_) mixed with 2.5 g of quartz sand was pretreated in 25% H_2_/N_2_ flow (50.0 mL min^−1^) at 220 °C for 2 h. After the temperature was cooled to reaction temperature in N_2_ atmosphere, a water and methanol mixture with molar ratio of 2 was fed into the reactor by an injection pump at a rate of 0.040 mL min^−1^. The reactants mixing with He gas (50.0 mL min^−1^) were evaporated at 140 °C before entering the reactor to avoid product condensation. The react temperature was monitored by K-type thermocouple. The products were analyzed online by Shimadzu GC-17A (TDX-01 and HP-PLOT/Q columns) equipped with both FID and TCD detectors. The methanol conversion (*X*_MeOH_), CO_2_ selectivity (${S}_{{{{\mbox{CO}}}}_{2}}$) and H_2_ production rate $({R}_{{{{\mbox{H}}}}_{2}})$ were calculated as follows:

1 $${X}_{{{\mbox{MeOH}}}}=\frac{{F}_{{{\mbox{MeOH}}},{{\mbox{in}}}}-{F}_{{{\mbox{MeOH}}},{{\mbox{out}}}}}{{F}_{{{\mbox{MeOH}}},{{\mbox{in}}}}}\times 100\%$$

2 $${{{\mbox{S}}}_{C{O}_{2}}}=\frac{{F}_{{{\mbox{{C{O}}}_{2}}}}}{{F}_{{{\mbox{MeOH}}},{{{\mbox{in}}}}}}\times 100\%$$

3 $${R}_{{{{{{{\rm{H}}}}}}}_{2}}=\frac{{F}_{{{{{{{\rm{H}}}}}}}_{2}}}{m}$$

Where *F*_MeOH,in/out_ is the molar flow rate of methanol at the inlet/outlet of the reactor, respectively; ${F}_{{{{\mbox{H}}}}_{2}}$ and ${F}_{{{{\mbox{CO}}}}_{2}}$ denote the molar flow rate of H_2_ and CO_2_ at the reactor outlet; *m* is the catalyst mass.

### Reaction dynamics studies

For the measurement of activation energy (*E*_a_), reaction order, reaction rate of CH_3_OH $({r}_{{{{\mbox{CH}}}}_{3}{{\mbox{OH}}}})$, CD_3_OD ($({r}_{{{{\mbox{CD}}}}_{3}{{\mbox{OD}}}})$ and CH_3_OD $({r}_{{{{\mbox{CH}}}}_{3}{{\mbox{OD}}}})$ as well as the kinetic isotope effect (KIE) over various catalysts were studied under kinetic control conditions (methanol conversion less than 20%). Typically, the catalyst (40−60 mesh, 0.01−0.10 g) and quartz sand (SiO_2_, 40−60 mesh, equivalent volume of the catalyst) were mixed together and transferred into the reactor tube. Reaction conditions for activation energy (*E*_*a*_) measurement were as follows: liquid feed of CH_3_OH/H_2_O (S/C = 2) at 0.040−0.080 mL min^−1^ with He carrier gas (50 mL min^−1^) at 180−260 °C. The kinetic isotope effect (KIE) test conditions were the similar as those described above except the reaction temperature at 210 °C. For the determination of reaction order of CH_3_OH and H_2_O, the initial partial pressure of CH_3_OH and H_2_O was tuned within 13−56 kPa and 7−28 kPa, respectively.

More detailed experimental characterizations and computational methods are described in the Supplementary Information.

### Supplementary information

Supplementary Information

Peer Review File

### Source data

Source data

## Data Availability

The data that supports the results reported in this manuscript are provided in the Supplementary Information File and in the Source Data file. Additional data related to this study are available from the authors upon request. Source data are provided with this paper.
