# Supplementary material for: Designing Cu0−Cu+ dual sites for improved C−H bond fracture towards methanol steam reforming
Source: Nat Commun. 2023 Dec 2;14:7980. doi: 10.1038/s41467-023-43679-0 (PMC10693576; doi:10.1038/s41467-023-43679-0)
Supplement: Supplementary file 1 — Supplementary Information [file 41467_2023_43679_MOESM1_ESM.pdf]

Supplementary Information

Designing Cu^0−Cu^+ dual sites for improved C−H bond fracture towards methanol steam reforming

Hao Meng[1,2], Yusen Yang[1,2]*, Tianyao Shen[1], Zhiming Yin[1], Lei Wang[1,2], Wei Liu[1], Pan Yin[1], Zhen Ren[1], Lirong Zheng[3], Jian Zhang[1]*, Feng-Shou Xiao[1,4]*, and Min Wei[1,2]*

[1] State Key Laboratory of Chemical Resource Engineering, Beijing Advanced Innovation Center for Soft Matter Science and Engineering, Beijing University of Chemical Technology, Beijing 100029, P. R. China

[2] Quzhou Institute for Innovation in Resource Chemical Engineering, Quzhou 324000, P. R. China

[3] Institute of High Energy Physics, Chinese Academy of Sciences, Beijing 100049, P. R. China

[4] Key Lab of Biomass Chemical Engineering of Ministry of Education, College of Chemical and Biological Engineering, Zhejiang University, Hangzhou 310027, P. R. China

Author Information

* Corresponding authors. Tel: +86-10-64412131; Fax: +86-10-64425385.

E-mail addresses: yangyusen@buct.edu.cn (Y. Yang); jianzhangbuct@buct.edu.cn (J. Zhang); fsxiao@zju.edu.cn (F.-S. Xiao); weimin@mail.buct.edu.cn (M. Wei).

Supplementary methods

Characterizations. The metal contents of Cu and Al were analyzed by using inductively coupled plasma atomic emission spectrometry (ICP-AES, Shimadzu ICPS-7500), and the results were listed in Supplementary Table 1. X-ray diffraction (XRD) analysis was carried out on a Rigaku XRD-6000 diffractometer with a nickel-filtered Cu K_α radiation source (λ = 0.15418 nm) at 40 kV and 30 mA. The physical structure parameters were obtained from N_2 adsorption and desorption isotherms on a Quantachrome Auosorb-1C-VP analyzer. The specific surface area and pore structure were calculated based on the Brunauer-Emmett-Teller (BET) and Barret-Joyner-Halenda (BJH) models, respectively. Transmission electron microscopy (TEM) and high-resolution transmission electron microscope (HR-TEM) images were conducted on JEOL JEM-2010 electron microscope with an accelerating voltage of 200 kV. The scanning transmission electron microscopy (STEM), electron energy loss spectroscopy (EELS) were carried out on double spherical aberration corrected JEOL JEM-ARM200F electron microscope with an accelerating voltage of 200 kV. *Quasi-in situ* X-ray photoelectron spectroscopy (XPS) and Auger electron spectroscopy (AES) were performed using a VG Escalab 250 with Al K_α as a radiation source at 300 K under UHV (2×10^{-9} Torr). The sample was transferred into a sample rod in glove box after H_2 pre-treatment with C 1*s* (284.8 eV) correction. *In situ* XAFS spectra of Cu K-edge were obtained on transmission mode with a standard transmission ion chamber detector (Cu foil as reference) on the beamline 1W1B and 1W2B of the Beijing Synchrotron Radiation Facility (BSRF), Institute of High Energy Physics (IHEP), Chinese Academy of Sciences (CAS). The hydrogen temperature programmed reduction (H_2-TPR), hydrogen temperature programmed desorption (H_2-TPD), N_2O-titration experiments, carbon monoxide temperature programmed desorption (CO-TPD), the H_2/D_2 exchange and CH_3OH/H_2O pulse

experiments were carried out on a Micromeritics Chemi-Sorb 2920 instrument equipped with a Pfeiffer GSD350 mass spectrometer detector (MS). *In situ* Fourier transform infrared (FT-IR) and CO-diffuse reflectance infrared Fourier transformations spectroscopy (DRIFT) were conducted on a VERTEX 70 BRUKER spectrometer with a CaF window and mercury-cadmium-telluride (MCT) detector. For the *off situ* characterizations, after the reduction of catalyst precursor (yCuAlO$_x$) in a tube furnace, the sample was passivated by 0.5% O_2/N_2 mixture at room temperature to produce a thin oxide layer, so as to prevent the deep oxidation of sample in air.

N_2O-titration measurements. The catalyst precursor (yCuAlO$_x$) was reduced in H_2 at 220 °C for 1 h, followed by purging with Ar for 0.5 h and cooling down to 50 °C. Then, the sample was exposed to 5% N_2O/Ar flow (50 mL min^{-1}) at 50 °C for 1 h for the oxidation of surface copper species to Cu_2O. Subsequently, the sample was ramped from 50 to 300 °C at a rate of 10 °C min^{-1} in 25% H_2/Ar (50 mL min^{-1}). The consumed hydrogen amount (X) was calculated. The dispersion degree of Cu^0 (D_{Cu^0}) and the concentration of surface Cu^0 species (C_{Cu^0}) were calculated according to the following equations:

$$D_{Cu^0} = (2\times M_{Cu}\times X/(m_{cat}\times m_{Cu}))\times 100\% \quad (1)$$

$$C_{Cu^0} = D_{Cu^0}\times m_{Cu}/M_{Cu} \quad (2)$$

Where the m_{cat} is the catalyst mass, m_{Cu} is copper mass per unit mass of catalyst measured by ICP-AES, M_{Cu} is atomic mass of copper.

N_2O-CO TPD measurements. The catalyst precursor (yCuAlO$_x$) was firstly pretreated at 220 °C in a H_2 atmosphere for 1 h, followed by purging with Ar at 220 °C for 1 h. Then, the catalyst was exposed to 5% N_2O/Ar flow (50 mL min^{-1}) at 50 °C for 1 h for the oxidation of surface copper species to Cu_2O. After the temperature was decreased to 30 °C, 10 vol % CO with He as carrier gas

was introduced until a saturation adsorption. Subsequently, pure He was purged at 30 °C to remove physically-adsorbed CO, and the temperature was increased from 30 to 500 °C at a rate of 10 °C min^{-1} for the collection of signals. The amount of CO desorption value (A) corresponds to the amount of total surface copper species. The dispersion degree of total surface copper (Cu^{+} + Cu^{0}) species (D_{Cu}) and Cu^{+} species ($D_{Cu^{+}}$) as well as the concentration of surface Cu^{+} species ($C_{Cu^{+}}$) were calculated according to the following equations[1]:

$$D_{Cu} = \mathrm{MCu} \times \mathrm{A}/(m_{cat} \times m_{Cu}) \times 100\% \qquad (3)$$

$$D_{Cu^{+}} = D_{Cu} - D_{Cu^{0}} \qquad (4)$$

$$C_{Cu^{+}} = D_{Cu^{+}} \times m_{Cu}/\mathrm{M}_{Cu} \qquad (5)$$

The interfacial perimeter length ($L_{Cu^{0}\text{-}Cu^{+}}$) was estimated based on the follow equations, in which the Cu particles were assumed to have a hemispherical shape:

$$\mathrm{S} = D_{Cu} \times N_{A} \times X_{Cu}/(M \times N_{Cu}) \qquad (6)$$

$$d = 6M/(\sigma \times \rho \times D_{Cu} \times N_{A}) \qquad (7)$$

$$L_{Cu^{0}\text{-}Cu^{+}} = 2S/d \qquad (8)$$

where X_{Cu} is the copper content measured by ICP-AES; M is the Cu atom weight (63.546 g mol^{-1}); D_{Cu} is the dispersion degree of total Cu; S is total surface area of Cu species; N_{Cu} is the number of surface Cu atoms in one square meter area (1.46 × 10^{19} m^{-2}); σ is the area occupied by a surface Cu atom (6.85 $Å^{2}$ per atom); ρ denotes the density of metallic Cu (8.94 g cm^{-3}), and N_{A} is Avogadro's number (6.022 × 10^{23} mol^{-1}).

Cu_2O/Cu fraction from EXAFS spectra. A normalization treatment was performed to correlate the coordination number (CN) of Cu–O bond (or Cu–Cu) with the Cu_2O/Cu ratio for these samples. The calculation equations are as follows:

$$X_{Cu_2O} = \mathrm{CN}_{Cu\text{-}O}/\mathrm{CN}_{Cu_2O} \qquad (9)$$

$$X_{Cu} = CN_{Cu\text{-}Cu}/CN_{Cu} \qquad (10)$$

$$\gamma = X_{Cu_2O}/X_{Cu} \qquad (11)$$

where the CN_{Cu_2O} and CN_{Cu} are 2 and 12, corresponding to the CNs in the first shell of Cu–O in Cu_2O and the Cu–Cu bond in Cu standard sample, respectively; the $CN_{Cu\text{–}O}$ and $CN_{Cu\text{–}Cu}$ denote the CNs of Cu–O and Cu–Cu bond obtained from EXAFS (Supplementary Table 2), and γ is the Cu_2O/Cu ratio for these samples.

H_2-TPD and H_2-TPR measurements. In the case of H_2-TPR measurement, the yCuAlO$_x$ sample was firstly pretreated in Ar atmosphere at 250 °C for 1 h and then cooled to 40 °C. After switching to a 25% H_2/Ar mixture gas, a typical temperature programming procedure was conducted from 40 to 700 °C at a rate of 10 °C min^{-1}. For the H_2-TPD, the catalyst precursor yCuAlO$_x$ was firstly pretreated at 220 °C in a H_2 atmosphere for 1 h, followed by purging with Ar at 220 °C for 1 h. After the temperature was decreased to 40 °C, 25 vol % H_2 with Ar as carrier gas was introduced until a saturation adsorption. Then, pure Ar was purged at 40 °C to remove physically-adsorbed H_2, along with the increase of temperature from 40 to 700 °C at a rate of 10 K min^{-1} for the collection of signals.

***Operando* pulse experiments.** The yCuAlO$_x$ precursor was firstly pretreated at 220 °C in a H_2 atmosphere for 1 h, followed by purging with Ar at 220 °C for 1 h. When the reaction temperature reached the preset temperature, the methanol or methyl formate vapor (60 °C) was pulsed into the reactor with Ar (50 mL min^{-1}) as carrier gas, and the signals of vary intermediates were captured by mass spectrometer detector (ThermoStar GSD 350).

***In situ* FT-IR measurements.** *In situ* Fourier transform infrared (FT-IR) spectra were obtained on a VERTEX 70 BRUKER spectrometer with a CaF window and mercury-cadmium-telluride (MCT) detector. In a typical measurement, 30 mg of yCu/Cu(Al)O$_x$ sample was pressured into a tablet and

placed in an *in situ* cell. Before the tests, the sample was reduced in a 25% H_2/Ar mixture gas with 30 mL min^{-1} at 220 °C for 1 h, followed by switching to a pure He to remove surface H_2O. After reduction, the background spectrum was recorded at corresponding reaction temperature. Subsequently, various substrates (CH_3OH, CD_3OD, CH_2O, $HCOOCH_3$ and HCOOH) were bubbled by He flow (30 mL min^{-1}) at room temperature into the reaction cell for the collection of spectra *vs.* reaction time. Then, the H_2O was introduced into the reaction cell with a similar bubbled mode, and the spectra were continuously recorded at reaction temperature.

***In situ* CO-DRIFT measurements.** For the *in situ* CO-DRIFT measurement, 20 mg of *y*Cu/Cu(Al)O_x sample was packed into the sample cell and subjected to a reduction pretreatment (similar as the above conditions). After reduction, the reaction cell was cooled to 25 °C in a purge of argon flow, for the collection of background spectrum. Then, a gas flow of 1% CO/He (30 mL min^{-1}) was introduced into the reaction cell and the spectra were continuously recorded. After the saturation adsorption of CO, the inlet flow was switched to argon (30 mL min^{-1}) to purge physically-adsorbed CO. For the semi-quantitative analysis, we refer to the extinction coefficient for linear CO adsorption over Cu^0 (ε_{Cu^0} = 0.79) and Cu^+ (ε_{Cu^+} = 1.30) sites[2], and calculate the ratio of Cu^+/Cu^0 based on the follow equation:

$$Cu^+/Cu^0 = (A_{Cu^+}/\varepsilon_{Cu^+})/(A_{Cu^0}/\varepsilon_{Cu^0}) \qquad (12)$$

***Operando* XAFS measurements.** *Operando* X-ray absorption fine structure spectroscopy (XAFS) at the Cu K-edge (transmission mode) was carried out at the beamline 1W1B and 1W2B of the Beijing Synchrotron Radiation Facility (BSRF), Institute of High Energy Physics (IHEP), Chinese Academy of Sciences (CAS). Typically, the powdered sample (30 mg) was pressed into a self-supporting wafer and carefully installed into a reaction microdevice equipped with polyimide windows. Afterwards,

the sample was pre-reduced in a 25% H_2/Ar mixture gas (30 mL min^{-1}) at 220 °C for 1 h, followed by cooling down to 200 °C in a high-purity He stream (30 mL min^{-1}) for the collection of initial XAFS spectrum. Subsequently, saturated methanol/formaldehyde/methyl formate/formic acid steam (30 °C) carried by He (30 mL min^{-1}) was carefully evaporated into the *in situ* cell at 200 °C to collect XAFS signals after 5 min; afterwards, saturated water vapor (30 °C) carried by He (30 mL min^{-1}) was introduced into the *in situ* cell at 200 °C for the collection of XAFS spectra after 5 min. Finally, the gas flow was switched to a pure He (30 mL min^{-1}) to purge the catalyst surface at 200 °C for 10 min and then XAFS spectra were collected. All the XAFS data were processed using Athena software package[3], and the data ranges used for data fitting in k-range, R-range and S02 value are 2.5−12.0 $Å^{-1}$, 1.0–3.0 Å and 0.8, respectively.

Reaction order measurement. We changed the molar ratio of water to methanol as well as the flow rate of carrier gas to maintain a fixed partial pressure of one substrate and adjust the partial pressure of another. For the measurement of water reaction order, we performed 5 sets of water:methanol ratios (1:0.5, 1:0.75, 1:1, 1:1.5 and 1:2, respectively) with a constant flow velocity of methanol solution (0.04 mL min^{-1}), and the flow rate of carrier gas was 50.0, 63.3, 81.7, 91.5 and 104.3 mL min^{-1}, respectively. Under the above five sets of reaction conditions, the partial pressure of methanol vapor is approximately a fixed value (13.9 kPa), and the partial pressure of water vapor is 27.9, 20.8, 13.9, 10.4 and 6.9 kPa, respectively, calculated based on the gas state equation. Similarly, for the measurement of methanol reaction order, the water:methanol ratio and flow velocity of methanol solution are consistent with the above situation, and the flow rate of carrier gas is 50.0, 35.1, 25.0, 12.9 and 5.7 mL min^{-1}, respectively. The partial pressure of water vapor is approximately a fixed value (27.8 kPa), and the partial pressure of methanol vapor is 13.9, 20.9, 27.9, 41.8 and 55.8 kPa,

respectively.

Computational details. Density functional theory (DFT) calculations were performed in Vienna *ab initio* simulation package (VASP) with the generalized gradient approximation (GGA) using the Perdew-Burke-Ernzerhof (PBE) functional. The projected augmented wave (PAW) potentials were used to describe the ionic cores and valence electrons were also considered using a plane wave basis set with a kinetic energy cutoff of 400 eV. Geometry optimizations were performed with the force convergency smaller than 0.05 eV $Å^{-1}$, and the same convergency was applied for the location of transition states by the constrained optimizations. The original bulk structure was optimized before the construction of surfaces with the Monkhorst-Pack k-point of 3×3×1.

For surface calculations, the $p(1 \times 4)$ $CuAlO_2$(101) surface was simulated using a slab supercell approach with periodic boundary conditions. Meanwhile, the $p(2 \times 2)$ Cu_2O(111) surface was applied for the atomic simulation. Both slabs contain three layers of metal oxides, in which the top two layers are allowed to move freely. A vacuum region of 15 Å is used, which is large enough to avoid interactions between adsorbates and slab images. The Cu(111) surfaces were modeled using periodic 3 × 3 unit cells with four-layer slabs separated by a 15 Å vacuum region. A 3 × 3 ×1 Monkhorst-Pack k-point mesh was used to sample the surface Brillouin zone for both Cu surfaces[4,5]. The $CuAlO_2$(101) surface with 16 Cu atoms, 16 Al atoms and 32 O atoms were applied in all the calculations. The Cu_2O(111) surface contains 24 O atoms and 48 Cu atoms. A Cu_8 nanocluster with 8 Cu atoms cut from Cu(111) was used to describe the inter-surface between Cu_8 and $CuAlO_2$[6,7]. A Monkhorst-Pack k-point 3 × 3 × 1 was applied for all the calculations on surfaces. In addition, the effect from the Hubbard U corrections was considered beyond the accuracy of DFT calculations of GGA, where U value (employed as U-J) of 3 was applied for Cu[8].

Transition state (TS) searches were performed at the same theoretical level with the CI-NEB method. All the models are the most stable structure obtained through optimization and screening. The adsorption energy (E_{ads}) is calculated as

$$E_{ads} = E_{total} - (E_{slab} + E_g) \tag{13}$$

where E_{total} is the total energy after adsorption; E_{slab} is the energy of the clean slab before adsorption; and E_g is the energy of the free adsorbate in the gas phase.

The energy barrier (E_a) is obtained from the electronic energy difference between the transition state (E_{TS}) and its corresponding initial state (E_{IS}), which is calculated by

$$E_a = E_{TS} - E_{IS} \tag{14}$$

Supplementary figures

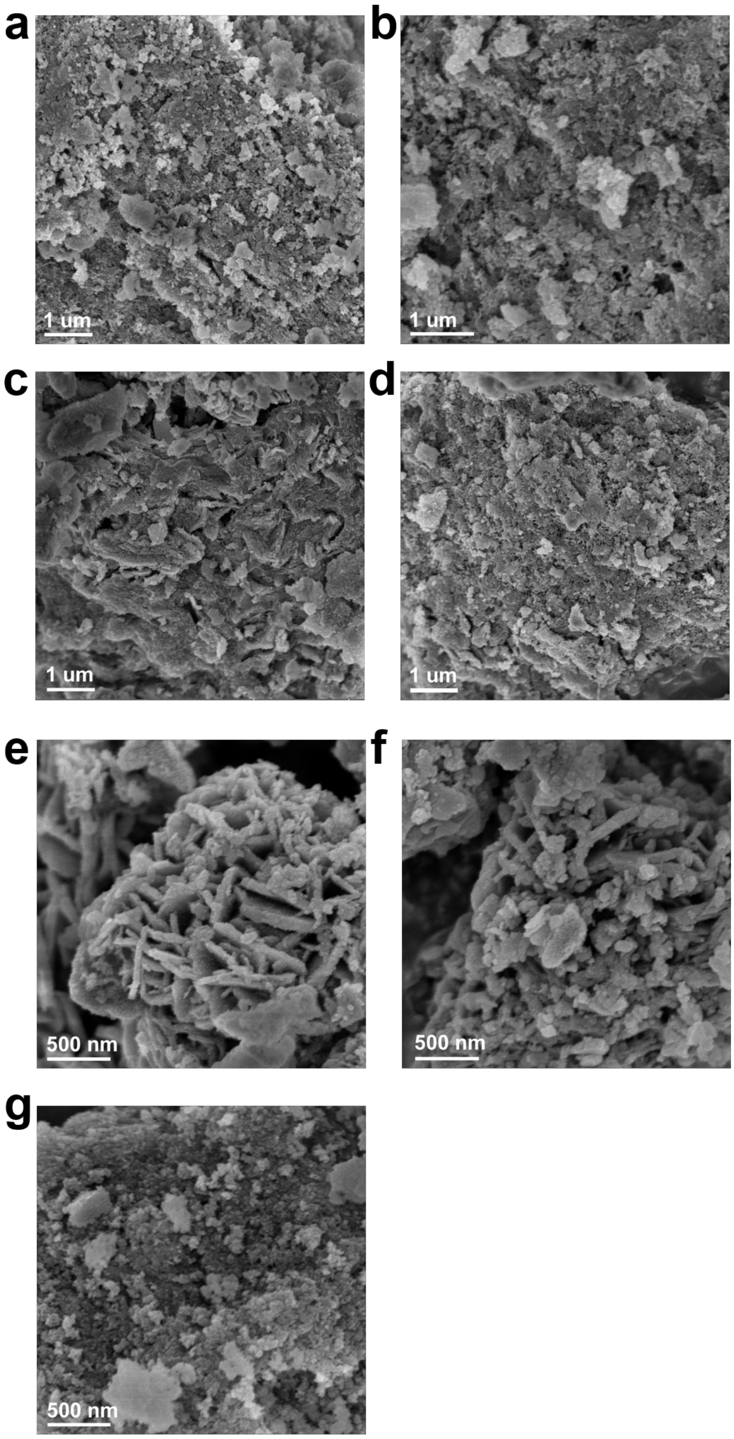

Supplementary Figure 1. Structure characterization of various samples. SEM images of **a** 0.95Cu/Cu(Al)O_x, **b** 2.32Cu/Cu(Al)O_x, **c** 3.06Cu/Cu(Al)O_x, **d** 4.25Cu/Cu(Al)O_x, **e** 5.27Cu/Cu(Al)O_x, **f** 7.18Cu/Cu(Al)O_x and **g** 4.20Cu/Al_2O_3 samples, respectively.

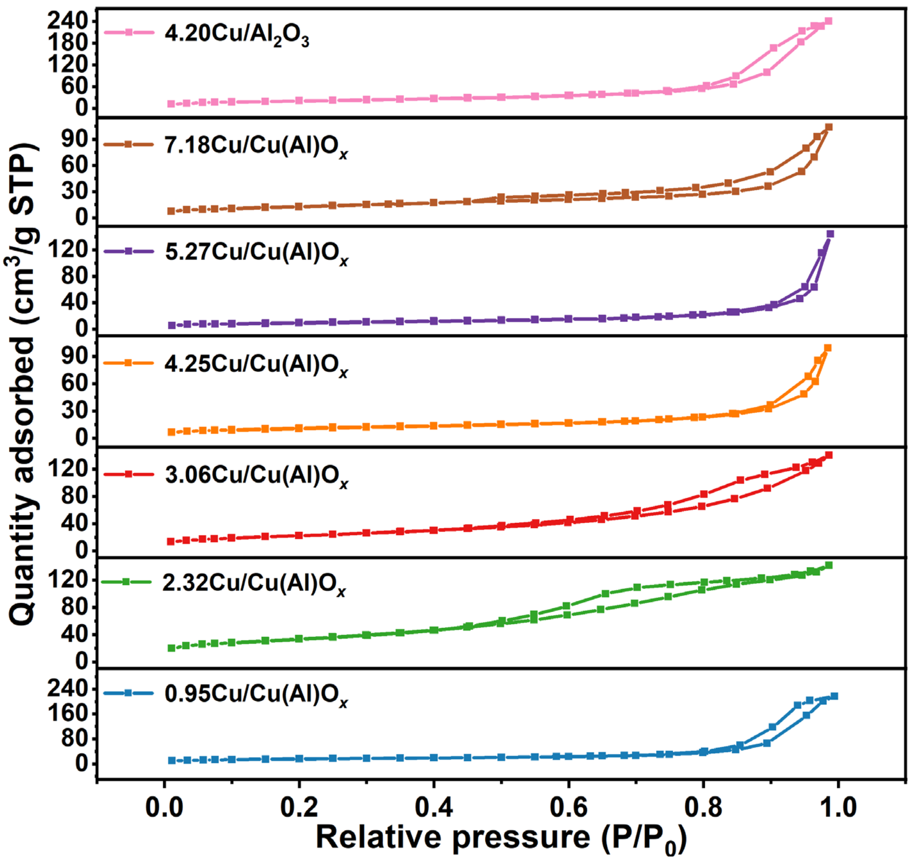

Supplementary Figure 2. N_2 adsorption–desorption experiment of various samples. N_2-sorption isotherm curves of 0.95Cu/Cu(Al)O_x, 2.32Cu/Cu(Al)O_x, 3.06Cu/Cu(Al)O_x, 4.25Cu/Cu(Al)O_x, 5.27Cu/Cu(Al)O_x, 7.18Cu/Cu(Al)O_x and 4.20Cu/Al_2O_3 samples, respectively.

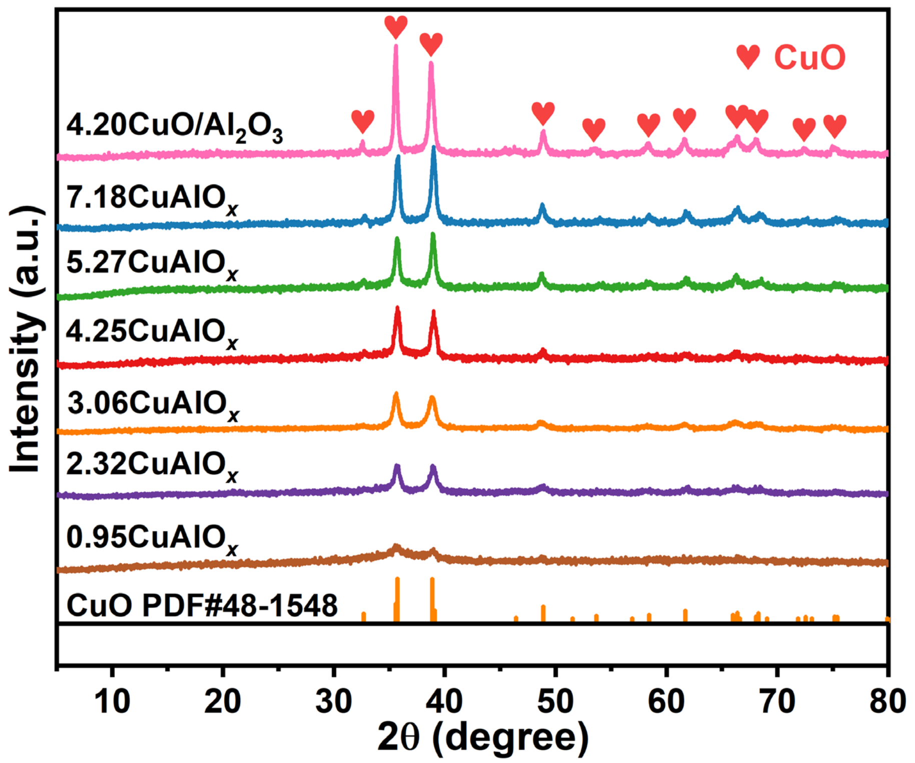

Supplementary Figure 3. Structure characterization of various samples. XRD patterns of the calcined 0.95CuAlO$_x$, 2.32CuAlO$_x$, 3.06Cu/CuAlO$_x$, 4.25CuAlO$_x$, 5.27CuAlO$_x$, 7.18CuAlO$_x$ and 4.20CuO/Al_2O_3 samples.

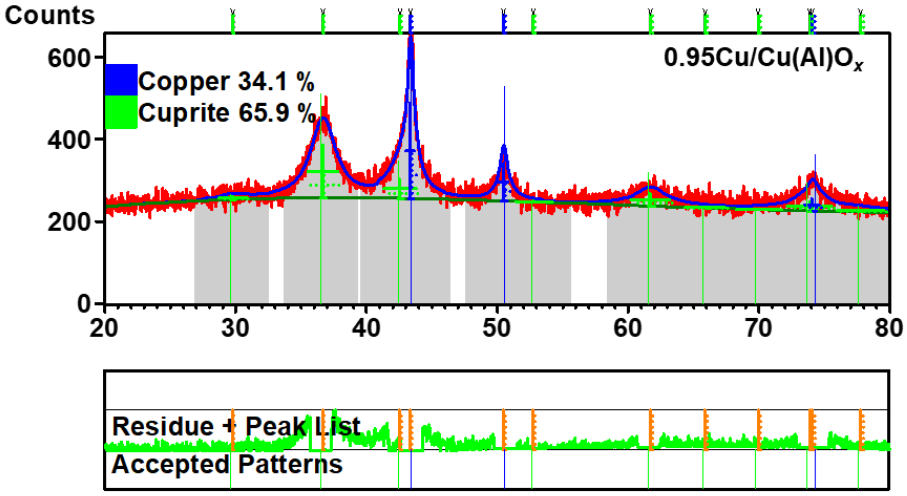

Supplementary Figure 4. Structure characterization. Rietveld analysis of XRD pattern for the 0.95Cu/Cu(Al)O$_x$ sample.

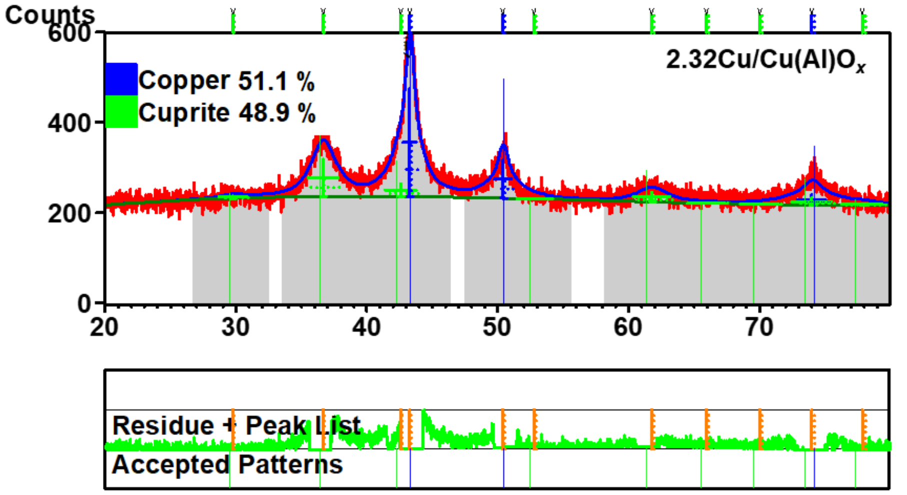

Supplementary Figure 5. Structure characterization. Rietveld analysis of XRD pattern for the 2.32Cu/Cu(Al)O$_x$ sample.

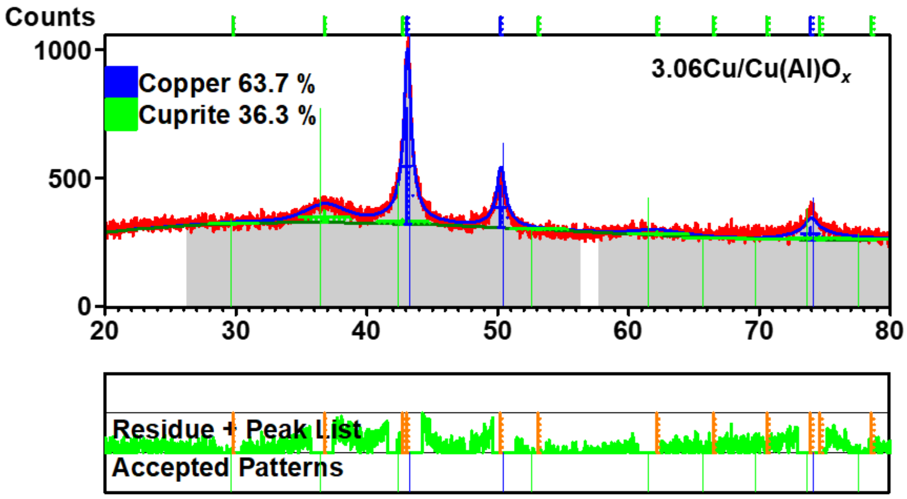

Supplementary Figure 6. Structure characterization. Rietveld analysis of XRD pattern for the 3.06Cu/Cu(Al)O_x sample.

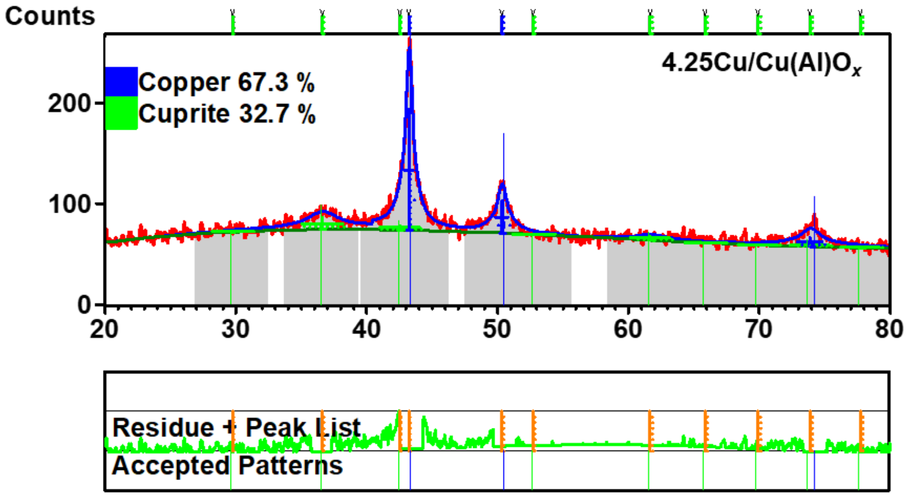

Supplementary Figure 7. Structure characterization. Rietveld analysis of XRD pattern for the 4.25Cu/Cu(Al)O_x sample.

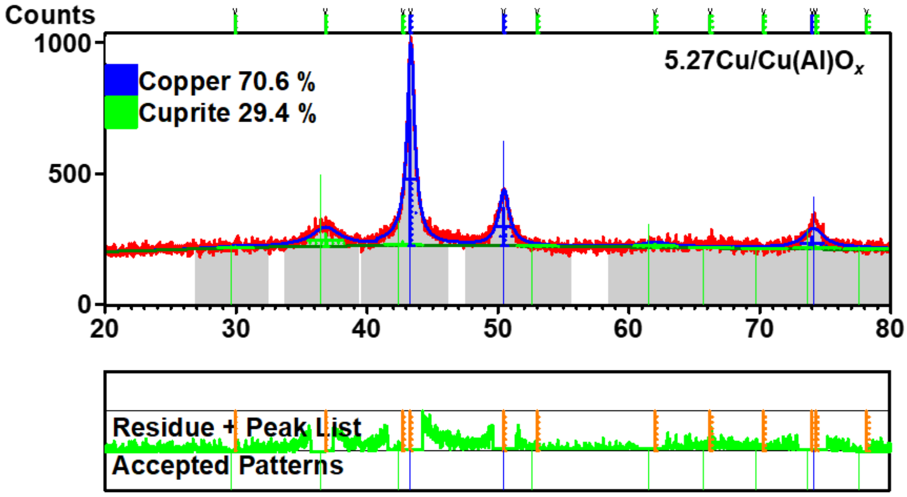

Supplementary Figure 8. Structure characterization. Rietveld analysis of XRD pattern for the 5.27Cu/Cu(Al)O_x sample.

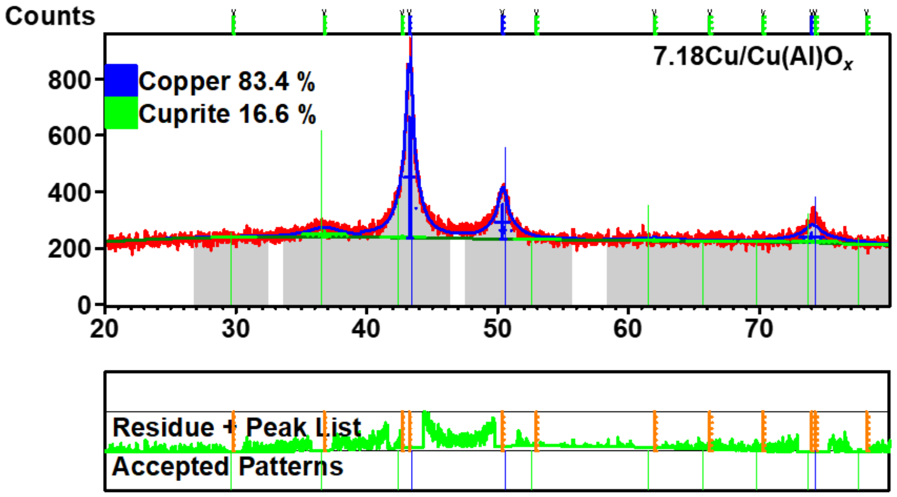

Supplementary Figure 9. Structure characterization. Rietveld analysis of XRD pattern for the 7.18Cu/Cu(Al)O_x sample.

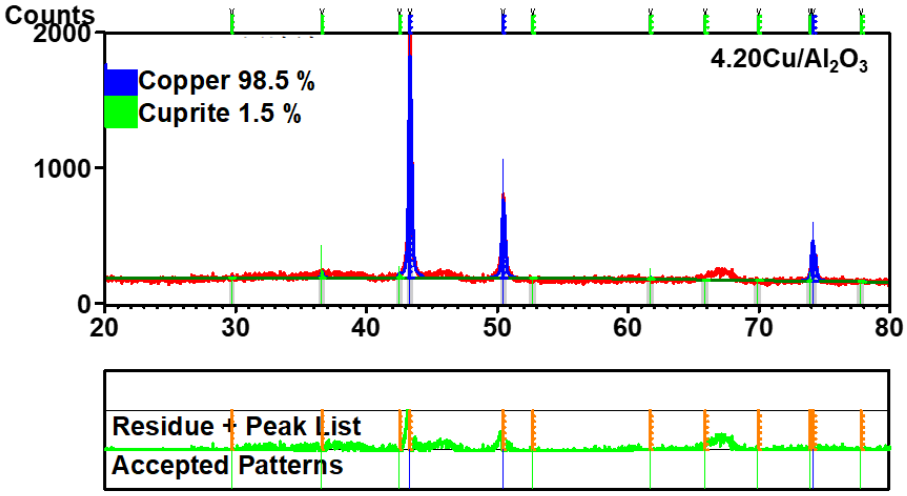

Supplementary Figure 10. Structure characterization. Rietveld analysis of XRD pattern for the 4.20Cu/Al_2O_3 sample.

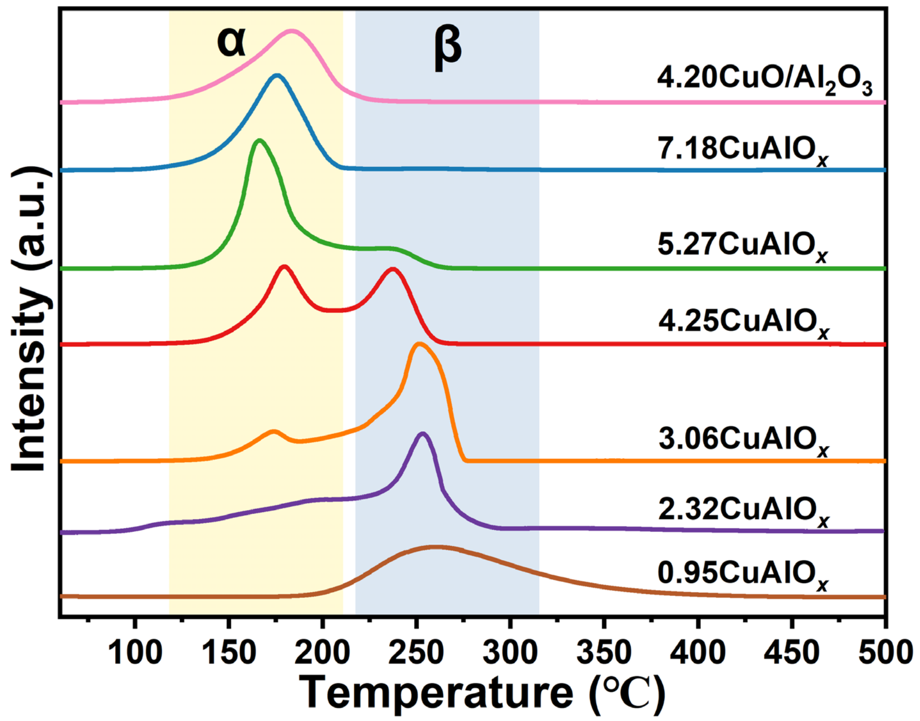

Supplementary Figure 11. H_2-TPR experiment of various samples. H_2-TPR curves of the calcined 0.95CuAlO$_x$, 2.32CuAlO$_x$, 3.06CuAlO$_x$, 4.25CuAlO$_x$, 5.27CuAlO$_x$, 7.18CuAlO$_x$ and 4.20CuO/Al_2O_3 samples.

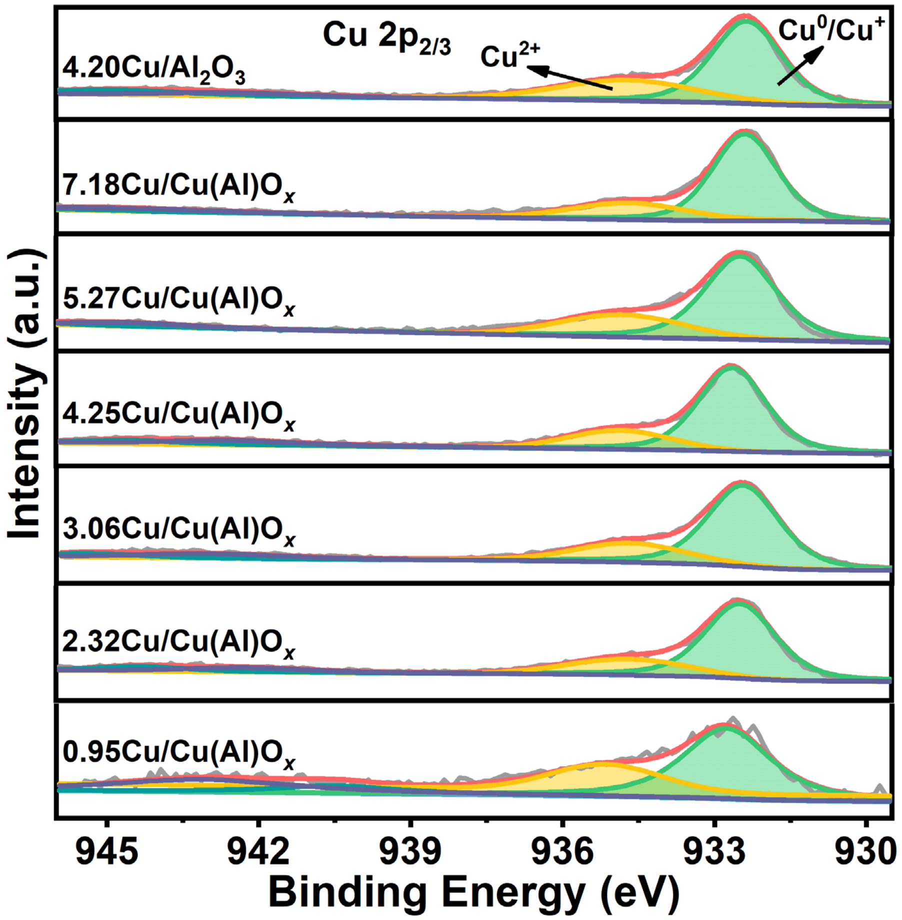

Supplementary Figure 12. XPS spectra of various samples. *Quasi-in situ* XPS spectra of Cu 2*p* for 0.95Cu/Cu(Al)O_x, 2.32Cu/Cu(Al)O_x, 3.06Cu/Cu(Al)O_x, 4.25Cu/Cu(Al)O_x, 5.27Cu/Cu(Al)O_x, 7.18Cu/Cu(Al)O_x and 4.20Cu/Al_2O_3 samples, respectively.

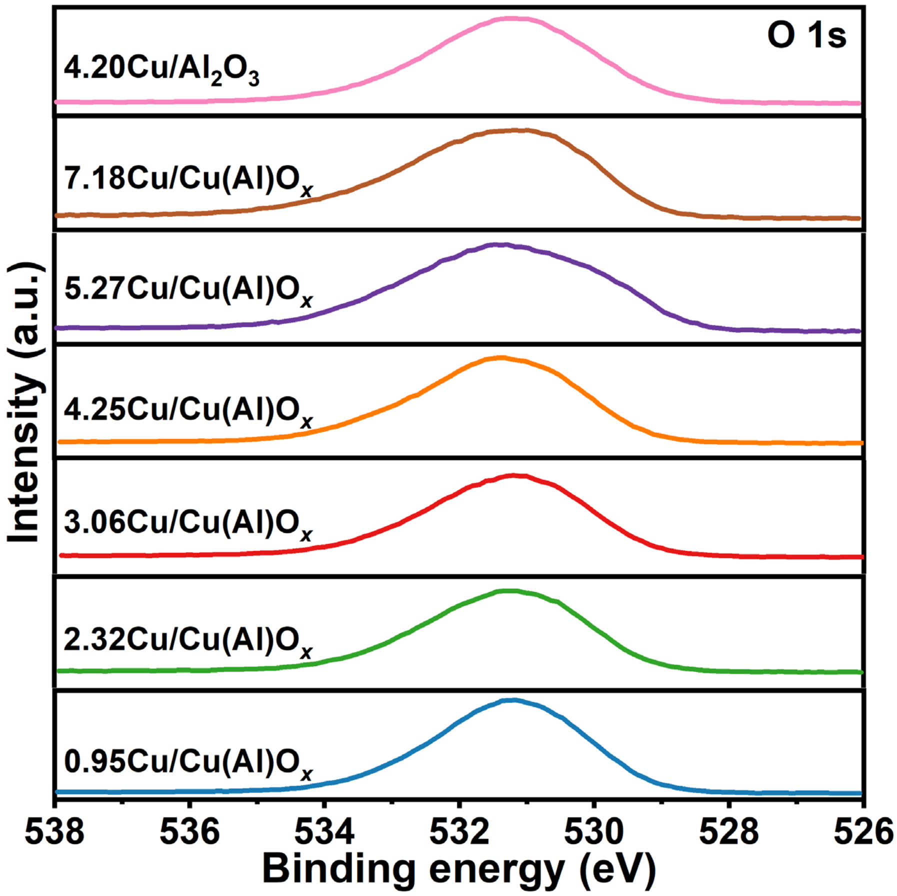

Supplementary Figure 13. XPS spectra of various samples. *Quasi-in situ* XPS spectra of O 1*s* for 0.95Cu/Cu(Al)O$_x$, 2.32Cu/Cu(Al)O$_x$, 3.06Cu/Cu(Al)O$_x$, 4.25Cu/Cu(Al)O$_x$, 5.27Cu/Cu(Al)O$_x$, 7.18Cu/Cu(Al)O$_x$ and 4.20Cu/Al_2O_3 samples, respectively.

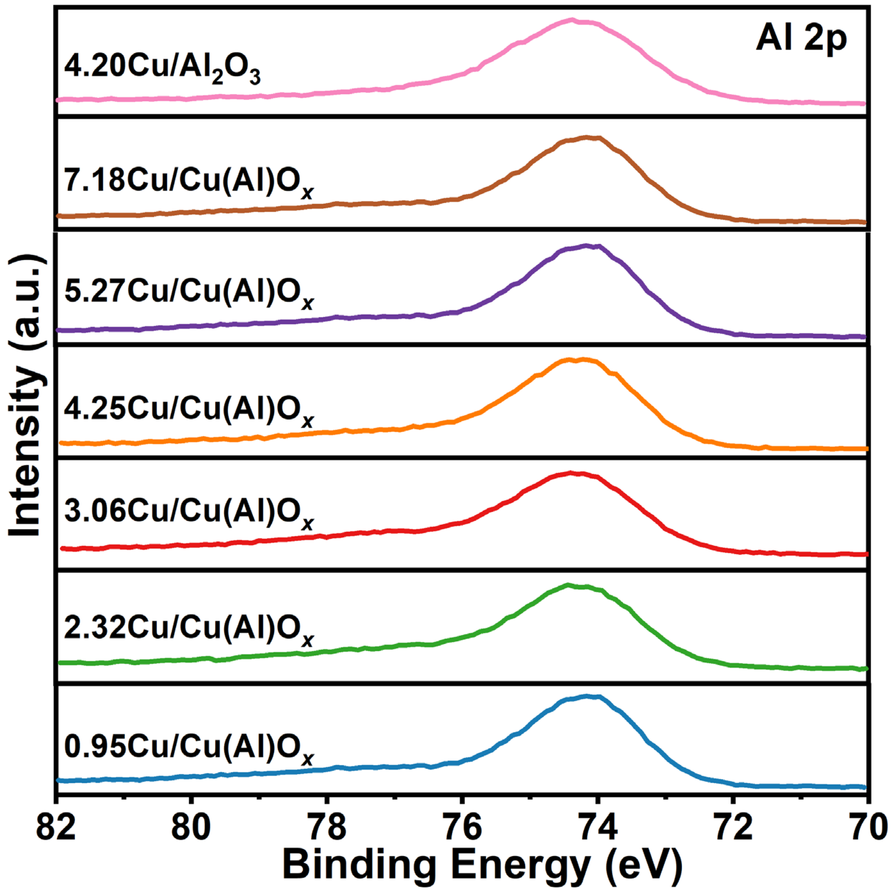

Supplementary Figure 14. XPS spectra of various samples. *Quasi-in situ* XPS spectra of Al 2*p* for 0.95Cu/Cu(Al)O_x, 2.32Cu/Cu(Al)O_x, 3.06Cu/Cu(Al)O_x, 4.25Cu/Cu(Al)O_x, 5.27Cu/Cu(Al)O_x, 7.18Cu/Cu(Al)O_x and 4.20Cu/Al_2O_3 samples, respectively.

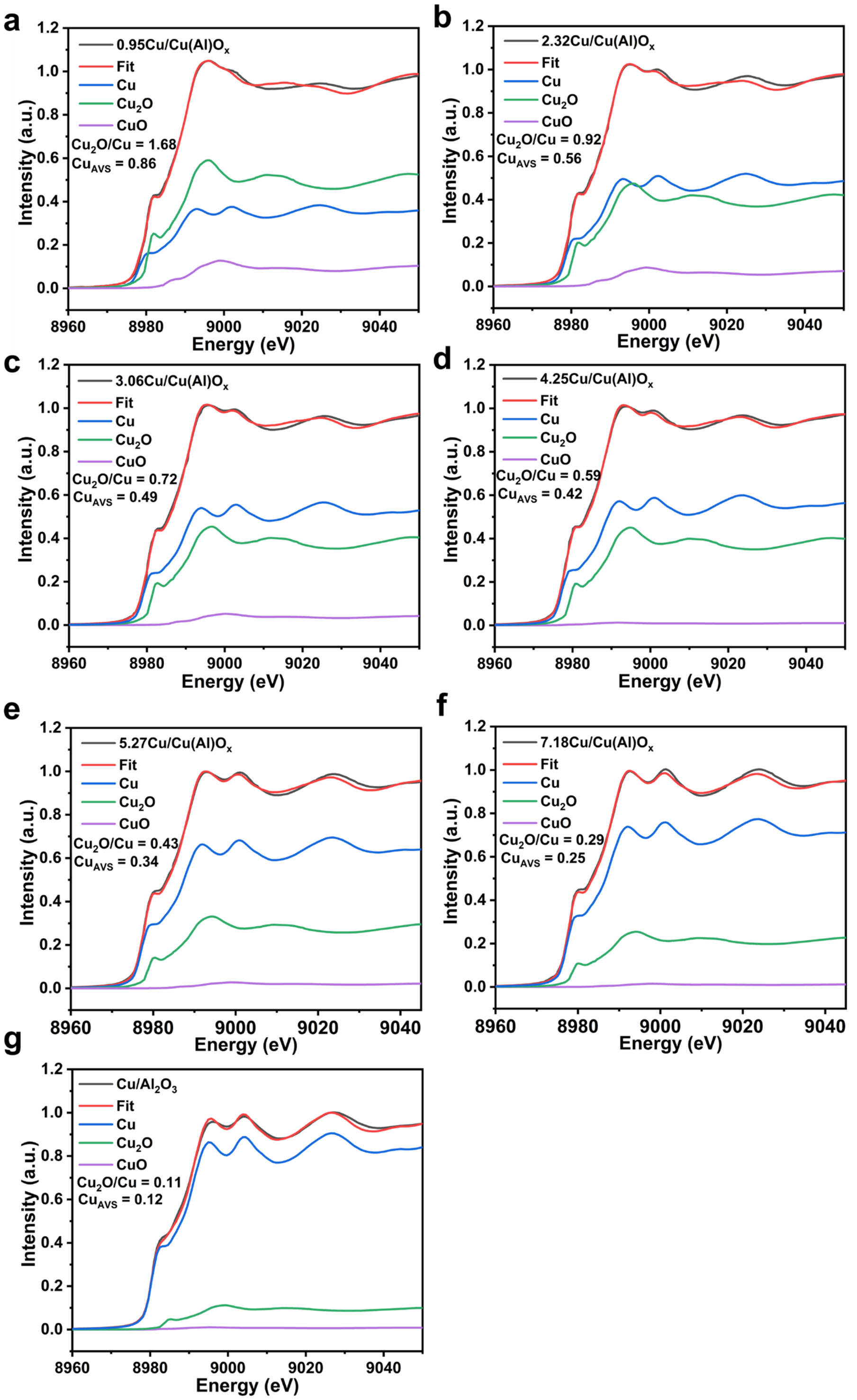

Supplementary Figure 15. XANES data fit of various samples. Linear combination fitting (LCF) analysis on the XANES data of **a** 0.95Cu/Cu(Al)O$_x$, **b** 2.32Cu/Cu(Al)O$_x$, **c** 3.06Cu/Cu(Al)O$_x$, **d** 4.25Cu/Cu(Al)O$_x$, **e** 5.27Cu/Cu(Al)O$_x$, **f** 7.18Cu/Cu(Al)O$_x$ and **g** 4.20Cu/Al$_2$O$_3$, respectively. Cu$_{AVS}$ is the average valence state of Cu species.

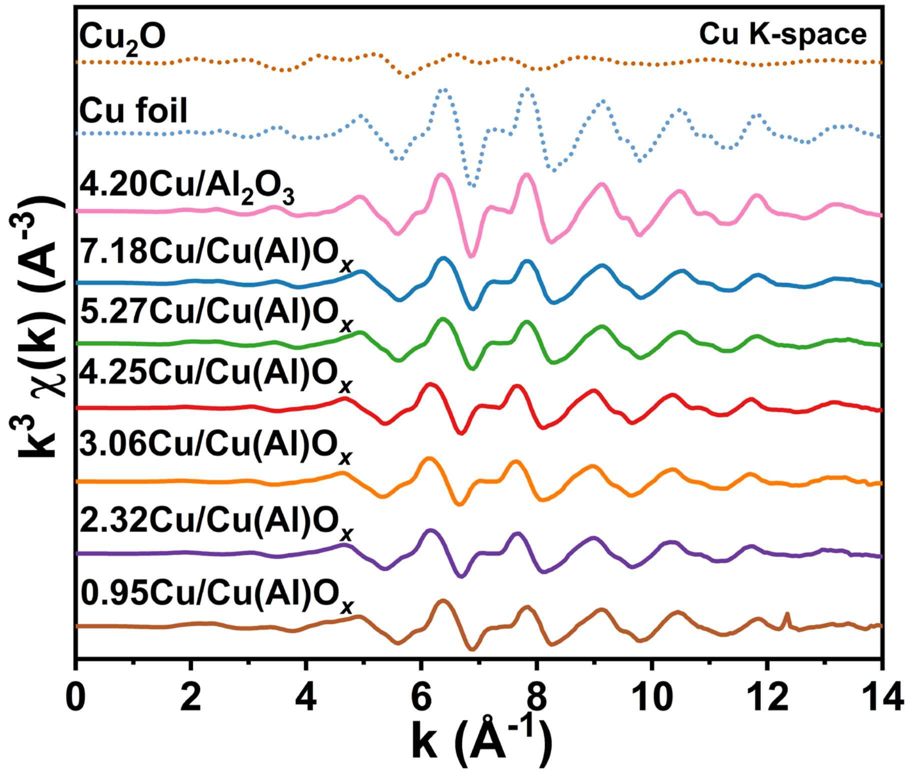

Supplementary Figure 16. Fine-structure characterization of various samples. Fourier-transform EXAFS spectra at Cu K-edge of 0.95Cu/Cu(Al)O_x, 2.32Cu/Cu(Al)O_x, 3.06Cu/Cu(Al)O_x, 4.25Cu/Cu(Al)O_x, 5.27Cu/Cu(Al)O_x, 7.18Cu/Cu(Al)O_x, 4.20Cu/Al_2O_3 and necessary standard samples.

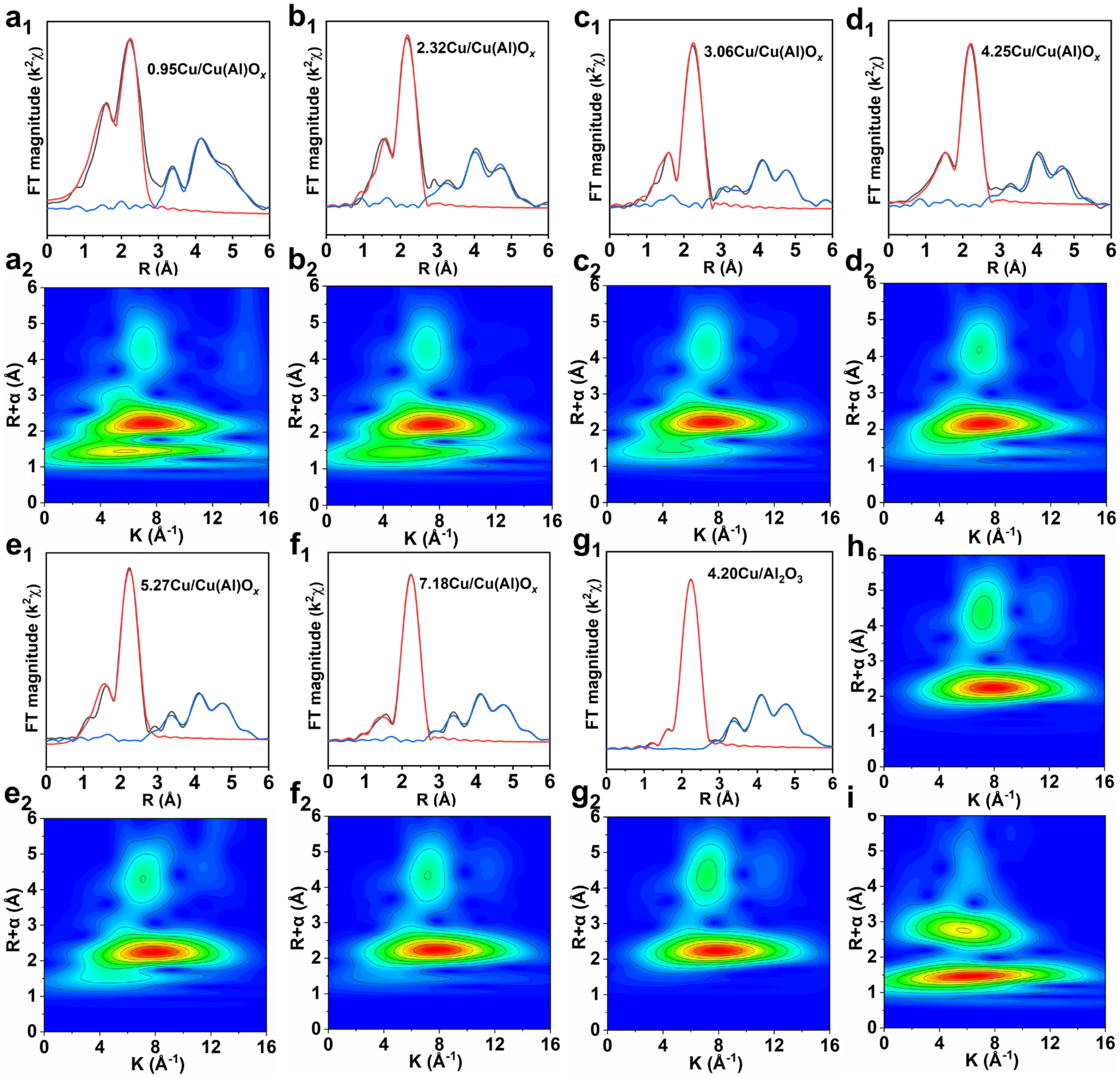

Supplementary Figure 17. Fine-structure characterization of various samples. Fitting results of **a_1–g_1** EXAFS spectra and **a_2–g_2** wavelet transform at Cu K-edge of **a_1,a_2** 0.95Cu/Cu(Al)O_x, **b_1,b_2** 2.32Cu/Cu(Al)O_x, **c_1,c_2** 3.06Cu/Cu(Al)O_x, **d_1,d_2** 4.25Cu/Cu(Al)O_x, **e_1,e_2** 5.27Cu/Cu(Al)O_x, **f_1,f_2** 7.18Cu/Cu(Al)O_x and **g_1,g_2** 4.20Cu/Cu(Al)O_x (the black line: experimental data; the red line: fitting curve; the blue line: residual data). Wavelet transform at Cu K-edge of **h** Cu foil and **i** Cu_2O standard samples.

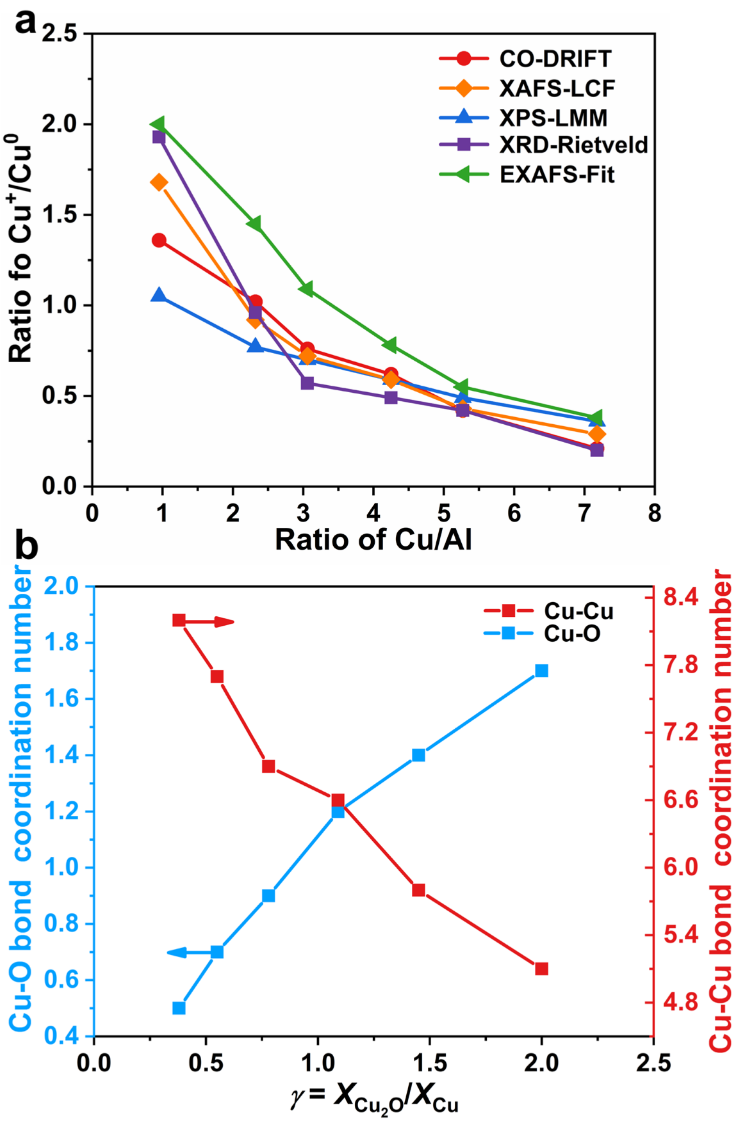

Supplementary Figure 18. Statistical analysis of various characterization results. a Ratios of Cu^{+}/Cu^{0} obtained from XRD Rietveld refinement, *quasi-in situ* Cu LMM, CO-DRIFT, XAFS-LCF and EXAFS-Fit analysis results as a function of Cu/Al molar ratio based on ICP-AES for these six *y*Cu/Cu(Al)O_x samples. **b** Coordination number of Cu–O or Cu–Cu bond as a function of Cu_2O/Cu ratio for various samples.

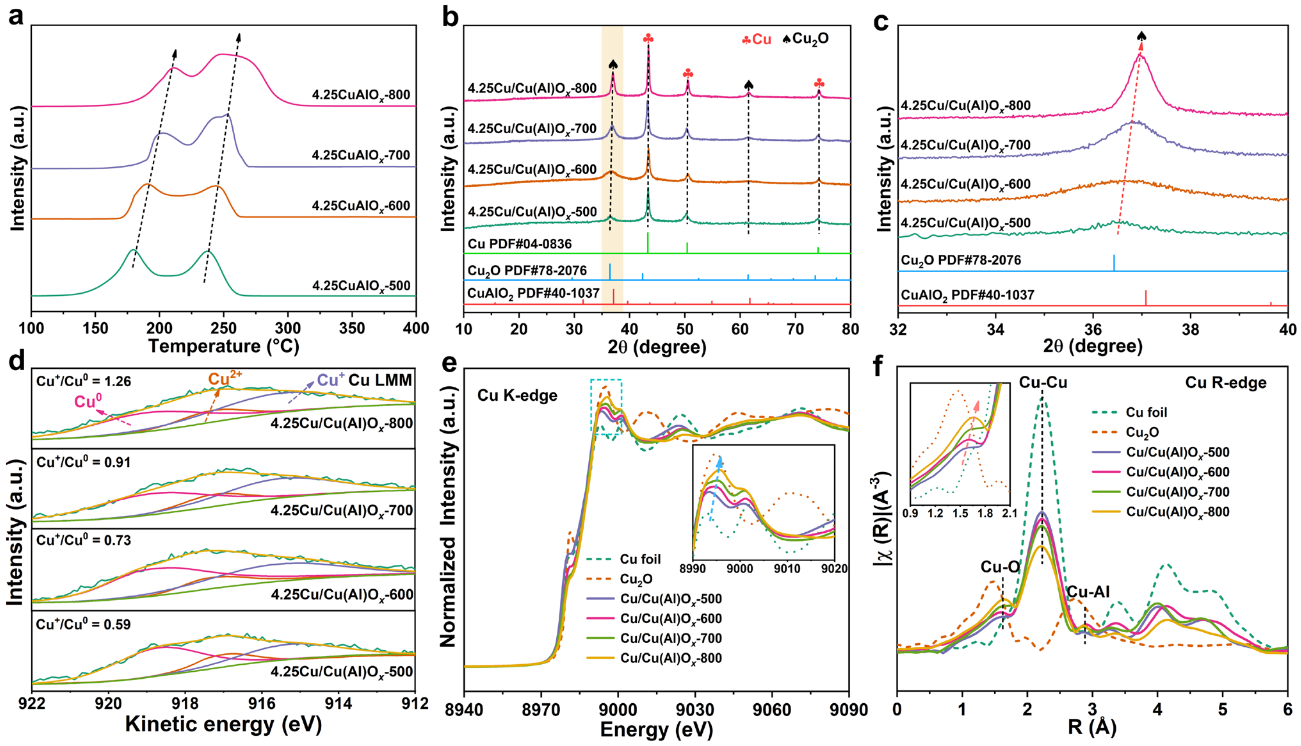

Supplementary Figure 19. Structure characterization of various samples. **a** H_2-TPR curves, **b,c** XRD patterns, **d** *quasi-in situ* Cu LMM AES spectra, **e** Cu K-edge XANES and **f** Cu K-edge EXAFS spectra of 4.25Cu/Cu(Al)O_x-y samples (y = 500, 600, 700, 800 °C, which denotes the roasting temperature of precursor. The 4.25Cu/Cu(Al)O_x-500 herein refers to the sample of 4.25Cu/Cu(Al)O_x in the manuscript).

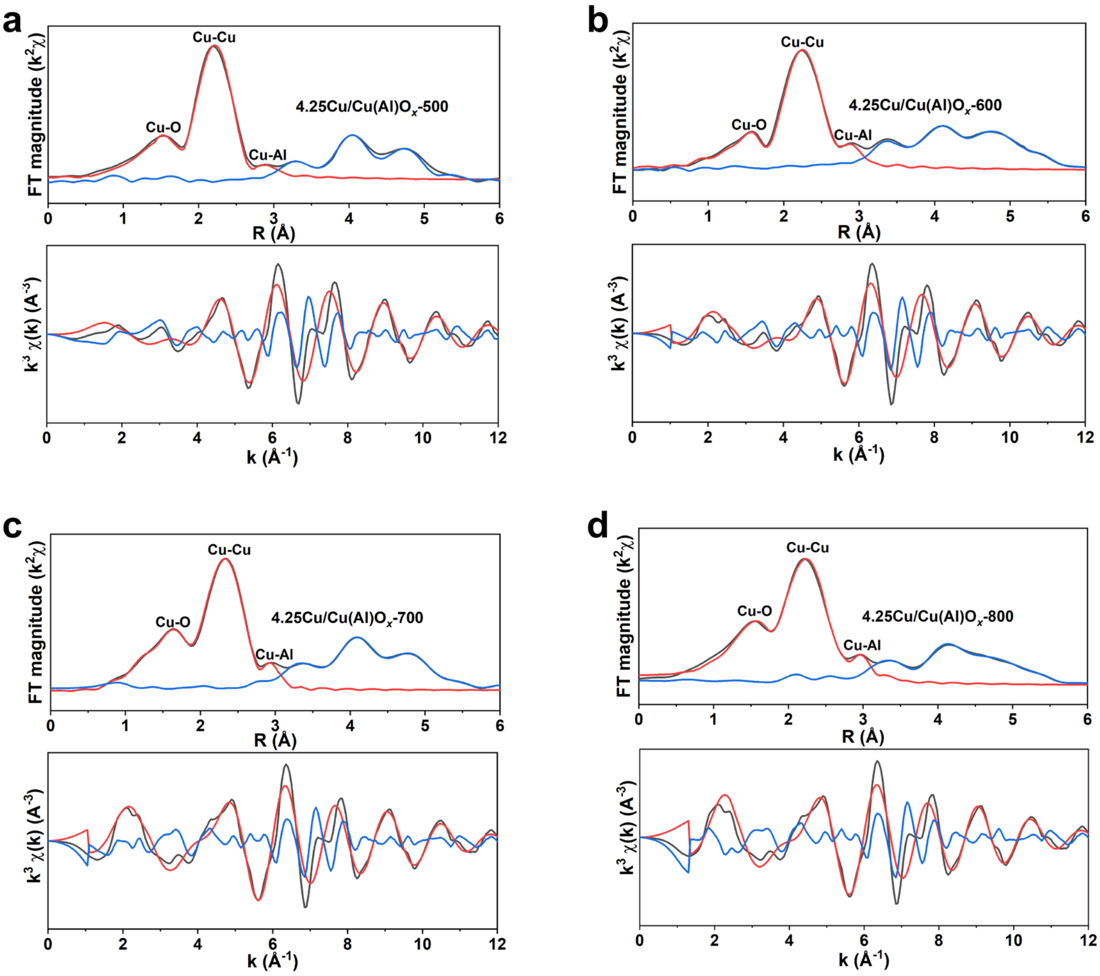

Supplementary Figure 20. Fine-structure characterization of various samples. Fitting results of EXAFS spectra at the Cu K-edge of **a** 4.25Cu/Cu(Al)O$_x$-500, **b** 4.25Cu/Cu(Al)O$_x$-600, **c** 4.25Cu/Cu(Al)O$_x$-700 and **d** 4.25Cu/Cu(Al)O$_x$-800 samples (black line: the experimental data; red line: the fitting curve; blue line: the residual data).

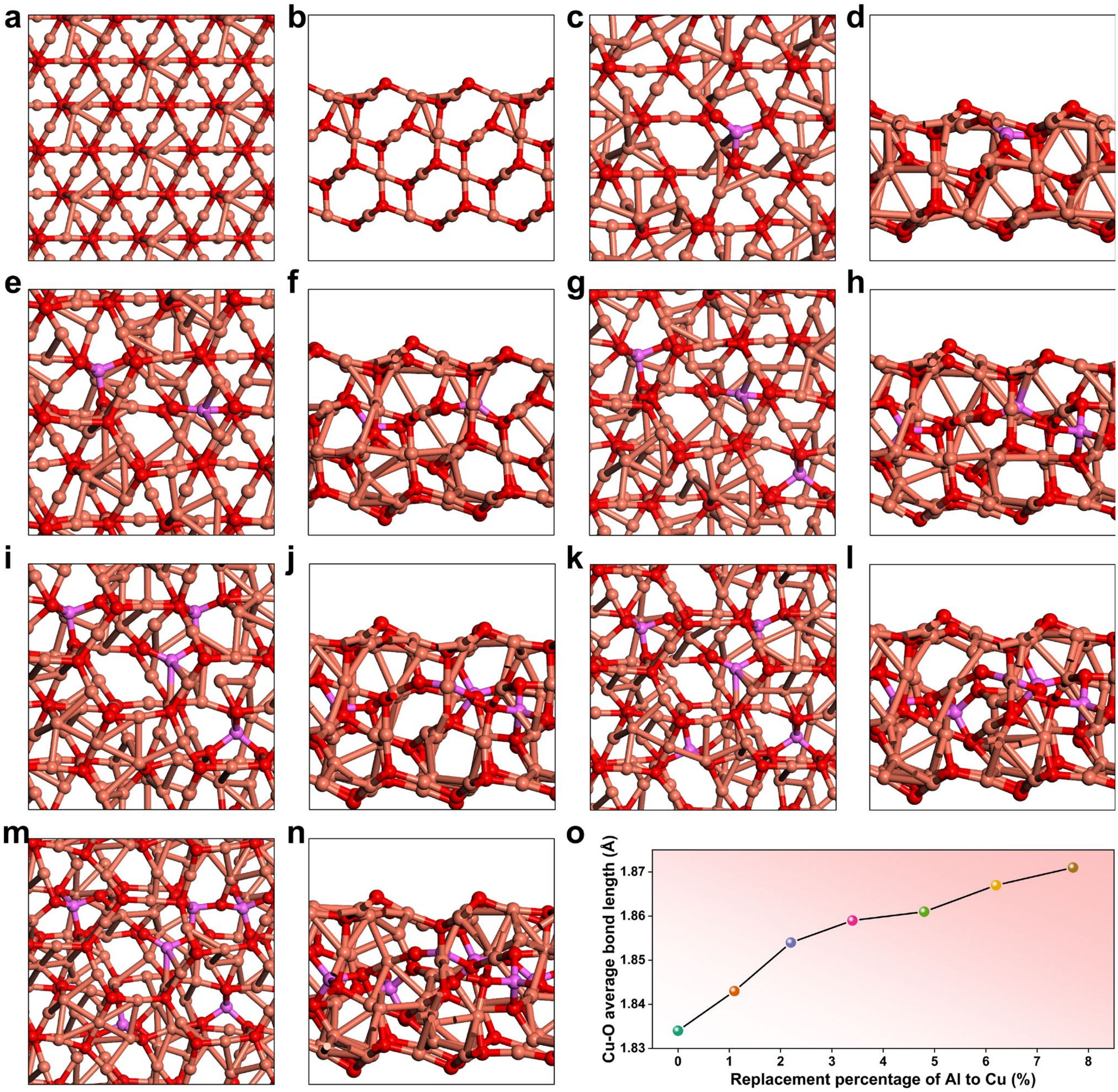

Supplementary Figure 21. DFT calculation of various models. Modified Cu_2O models with substitution of Cu^+ by Al^{3+} with various replacement percentage: **a,b** 0% (96 Cu and 48 O atoms), **c,d** 1.1% (93 Cu, 1 Al and 48 O atoms), **e,f** 2.2% (90 Cu, 2 Al and 48 O atoms), **g,h** 3.4% (87 Cu, 3 Al and 48 O atoms), **i,j** 4.8% (84 Cu, 4 Al and 48 O atoms), **k,l** 6.2% (81 Cu, 5 Al and 48 O atoms), **m,n** 7.7% (78 Cu, 6 Al and 48 O atoms). **o** Average bond length of Cu−O from 30 random data points as a function of replacement percentage of Al to Cu.

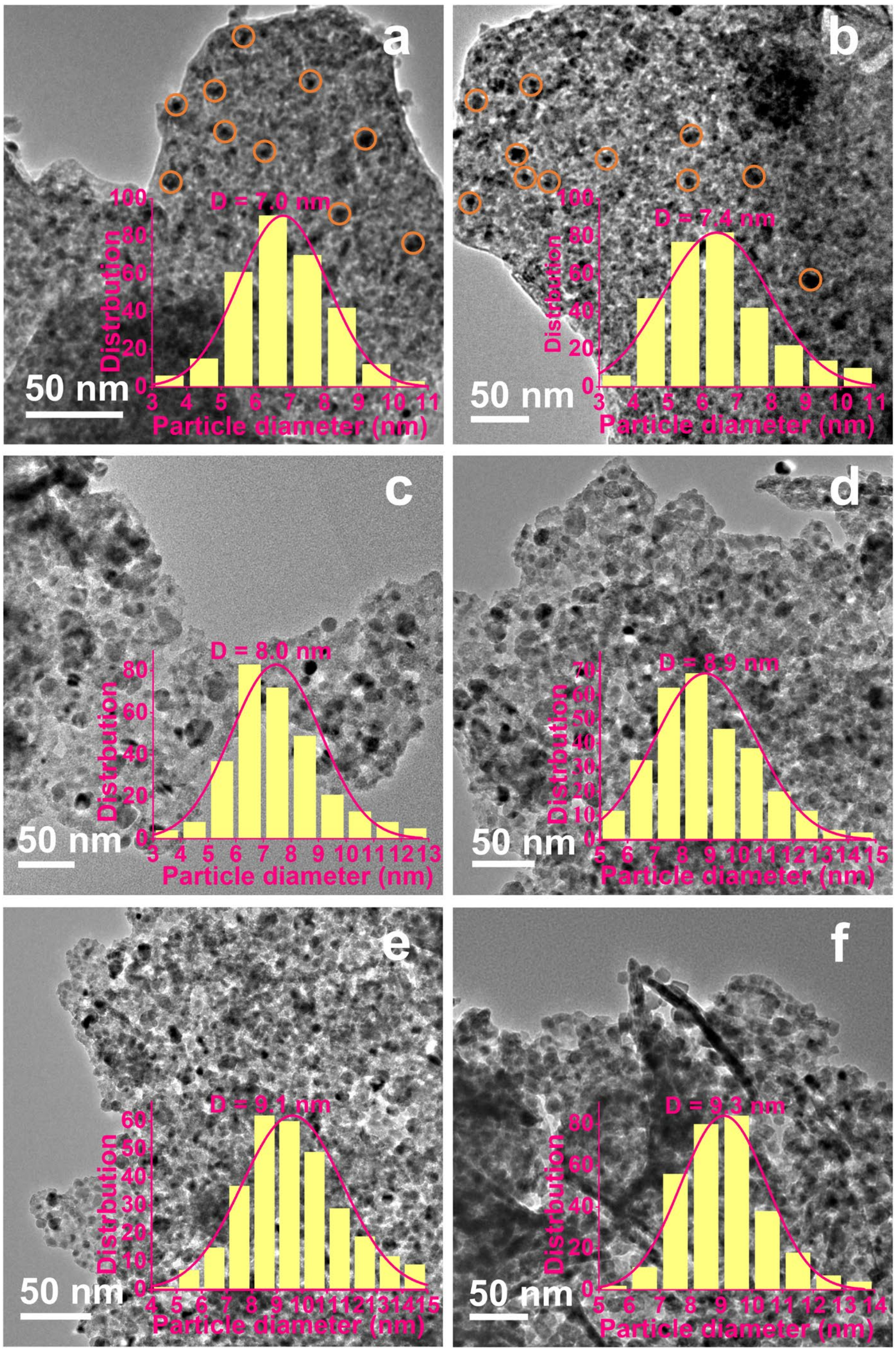

Supplementary Figure 22. Electron micrology studies on various samples. TEM images and particle size distributions of **a** 0.95Cu/Cu(Al)O$_x$, **b** 2.32Cu/Cu(Al)O$_x$, **c** 3.06Cu/Cu(Al)O$_x$, **d** 4.25Cu/Cu(Al)O$_x$, **e** 5.27Cu/Cu(Al)O$_x$ and **f** 7.18Cu/Cu(Al)O$_x$ samples, respectively.

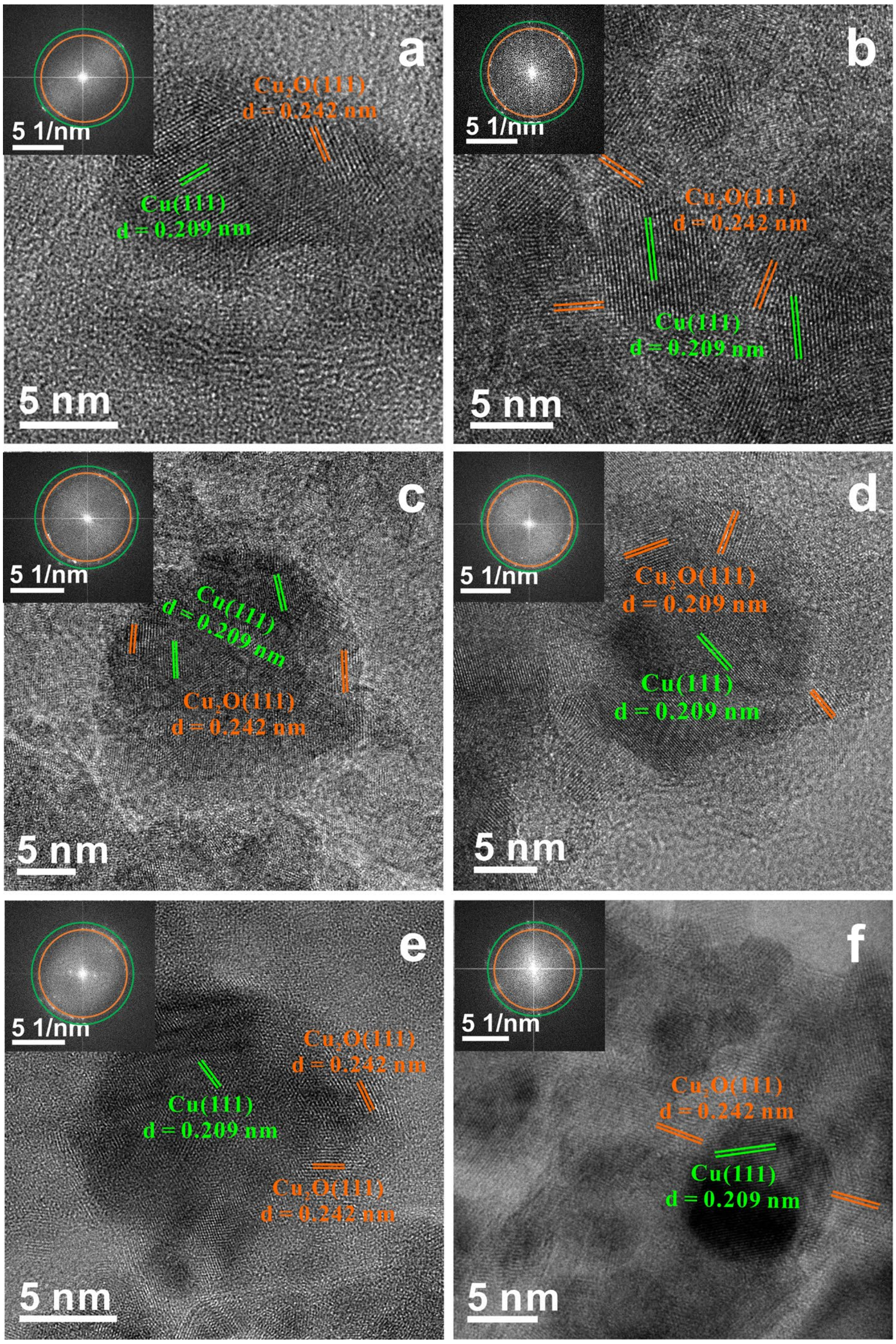

Supplementary Figure 23. Electron micrology studies on various samples. HR-TEM images of **a** 0.95Cu/Cu(Al)O_x, **b** 2.32Cu/Cu(Al)O_x, **c** 3.06Cu/Cu(Al)O_x, **d** 4.25Cu/Cu(Al)O_x, **e** 5.27Cu/Cu(Al)O_x and **f** 7.18Cu/Cu(Al)O_x samples (insets: fast Fourier transform (FFT) patterns of panel).

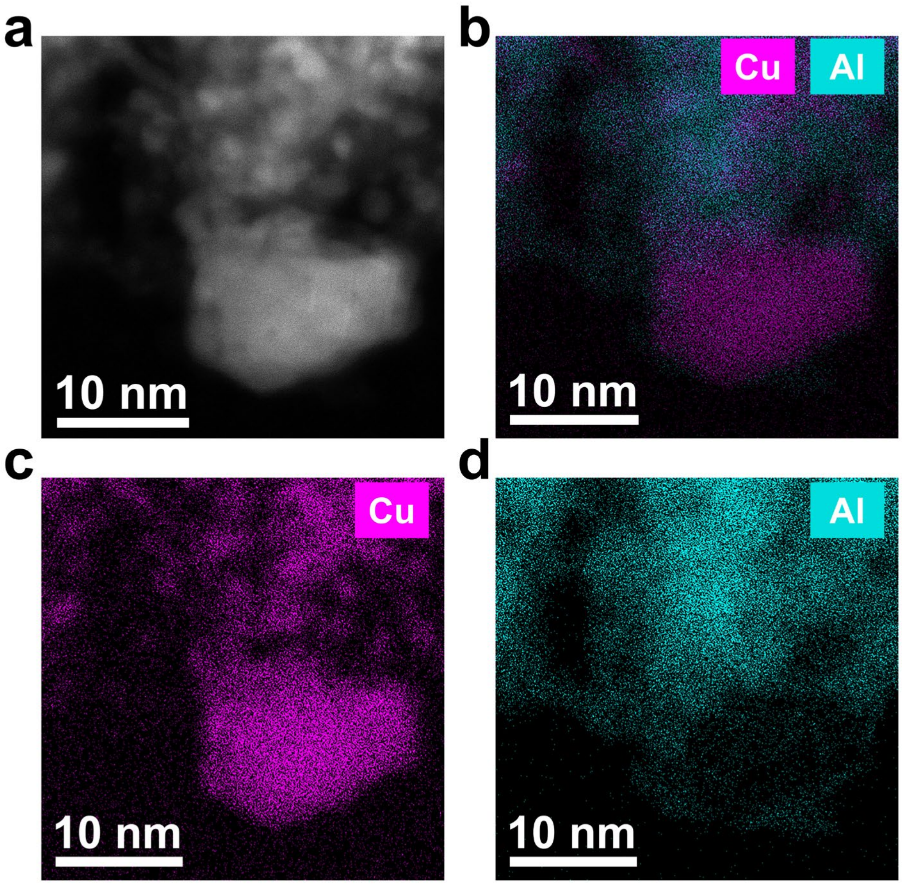

Supplementary Figure 24. EDS elemental mapping of 3.06Cu/Cu(Al)O_x. a TEM bright field image and **b–d** EDS elemental mapping images of the 3.06Cu/Cu(Al)O_x sample.

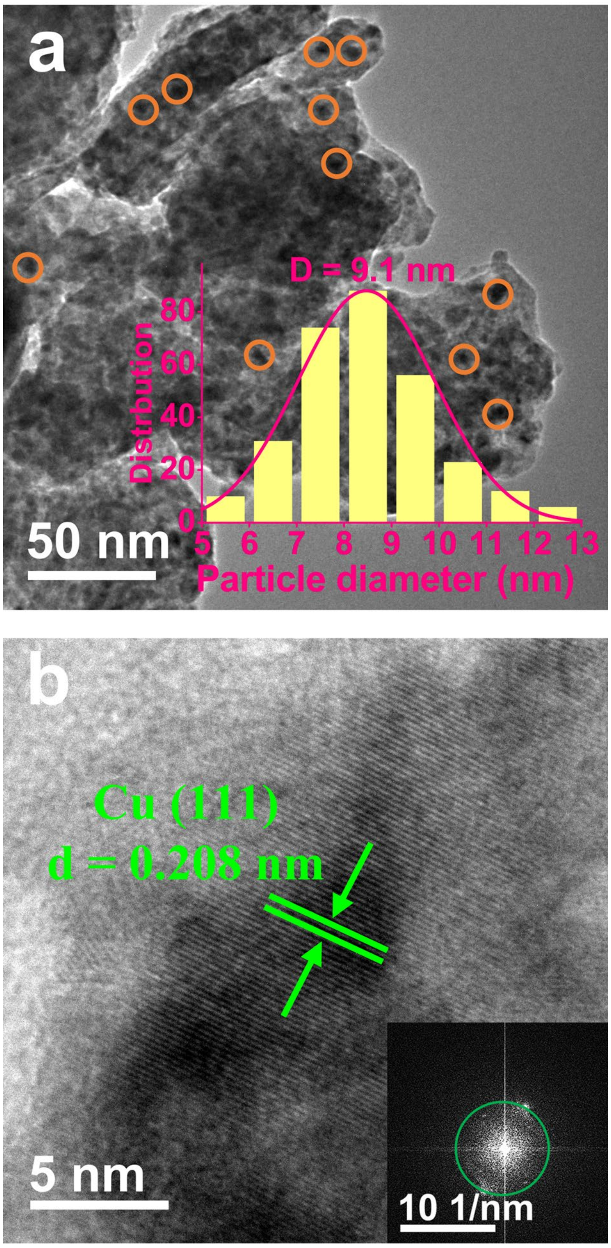

Supplementary Figure 25. Electron micrology studies on the 4.20Cu/Al_2O_3. a TEM (inset: the histogram for size distribution of Cu nanoparticles) image and **b** HR-TEM image of the 4.20Cu/Al_2O_3 sample (inset: fast Fourier transform (FFT) patterns of panel).

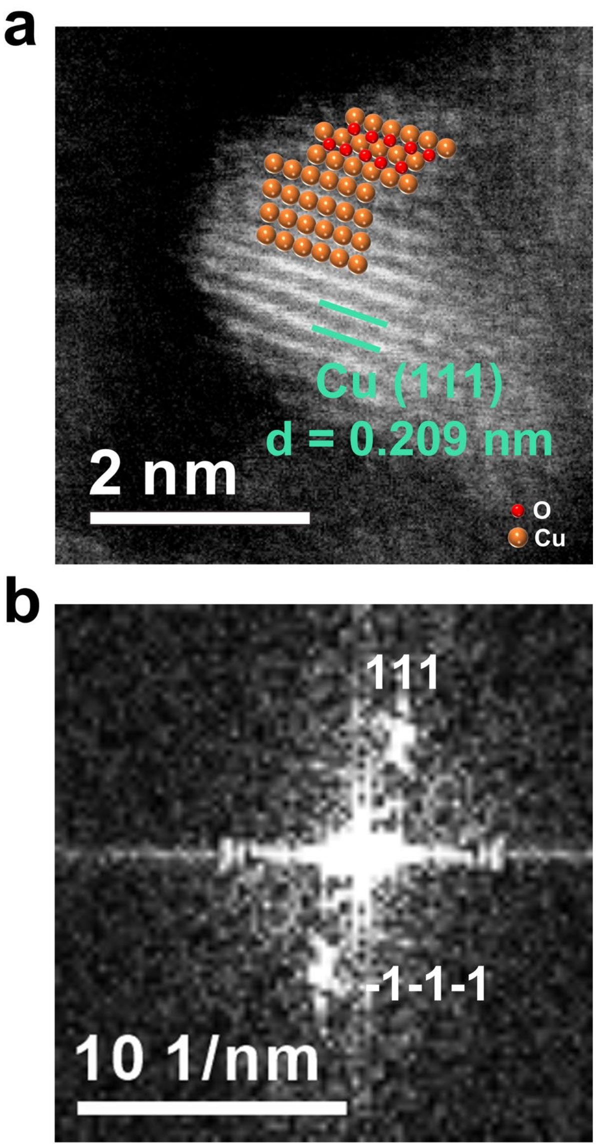

Supplementary Figure 26. Electron micrology studies on the 4.25Cu/Cu(Al)O$_x$. a High-resolution STEM image and **b** fast Fourier transform (FFT) patterns of the 4.25Cu/Cu(Al)O$_x$ sample.

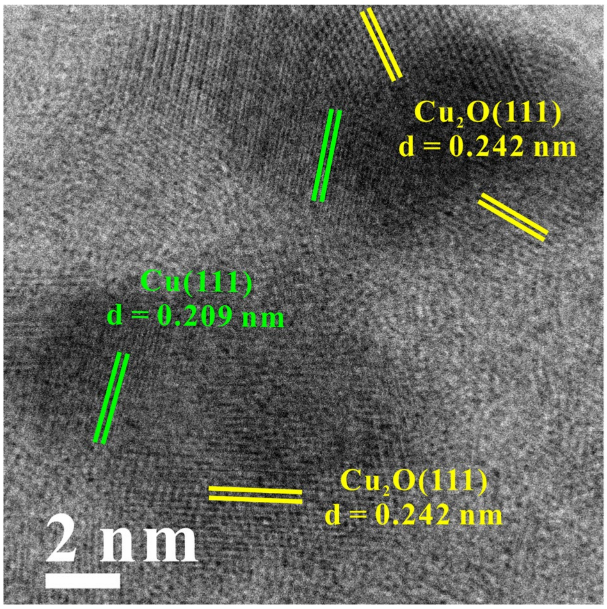

Supplementary Figure 27. Electron micrology studies on the 4.25Cu/Cu(Al)O_x. High-magnification STEM-BF image of the 4.25Cu/Cu(Al)O_x sample.

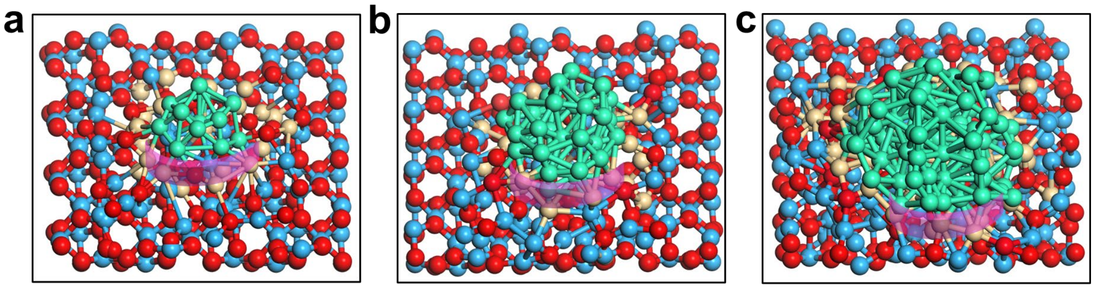

Supplementary Figure 28. Schematic diagram of Cu/Cu(Al)O$_x$. Schematic structure diagram of the Cu/Cu(Al)O$_x$ samples with increasing Cu loading (from **a** to **c**). The purple region indicates the Cu^0–Cu^+ interface sites. Red, earthy yellow, green and blue balls denote O, Cu^+, Cu^0 and Al, respectively.

Supplementary Note 1

As shown in Supplementary Fig. 28, both the size of Cu nanoparticles and the Cu/Al ratio increase with the increment of Cu loading. The Cu^0 site is located at the exterior surface of Cu nanoparticles whilst Cu^+ is located at the edge of Cu particles that has a strong interaction with Al_2O_3 support, resulting in the formation of specific Cu^0–Cu^+ interface sites as demonstrated by EELS results. XRD and EXAFS verify that such Cu^+ species exists as Cu_2O with partial doping of aluminum. However, this Cu^+ species is difficult to be reduced to a metallic state due to the stabilizing effect of amorphous alumina on Cu_2O, which becomes more significant with the increase of Al content as confirmed by H_2-TPR results.

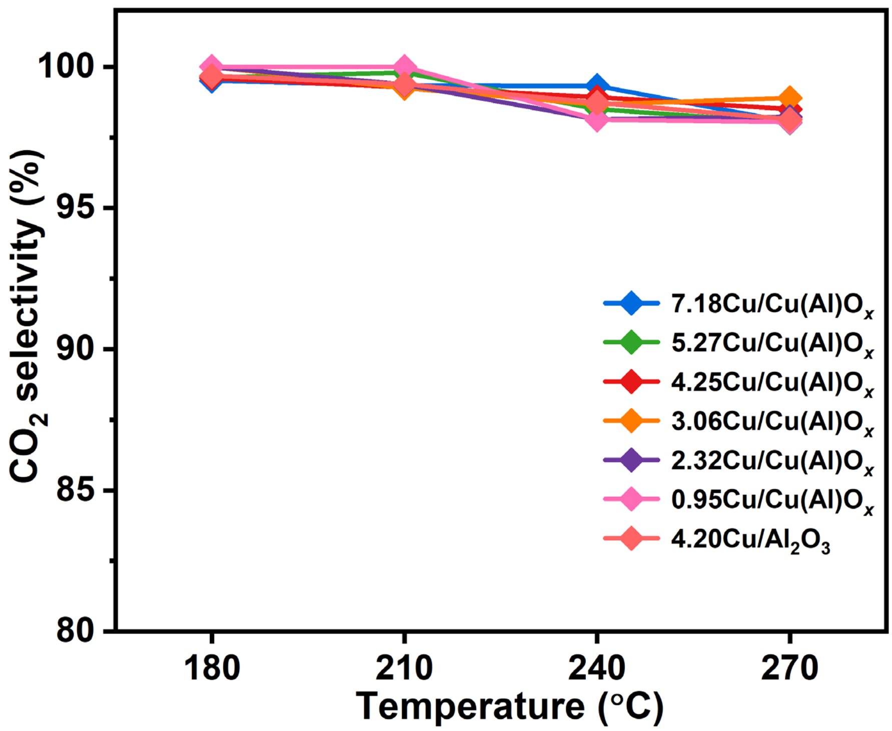

Supplementary Figure 29. CO_2 selectivity of various samples. CO_2 selectivity in MSR reaction over 0.95Cu/Cu(Al)O_x, 2.32Cu/Cu(Al)O_x, 3.06Cu/Cu(Al)O_x, 4.25Cu/Cu(Al)O_x, 5.27Cu/Cu(Al)O_x, 7.18Cu/Cu(Al)O_x and 4.20Cu/Al_2O_3 samples at 180–270 °C.

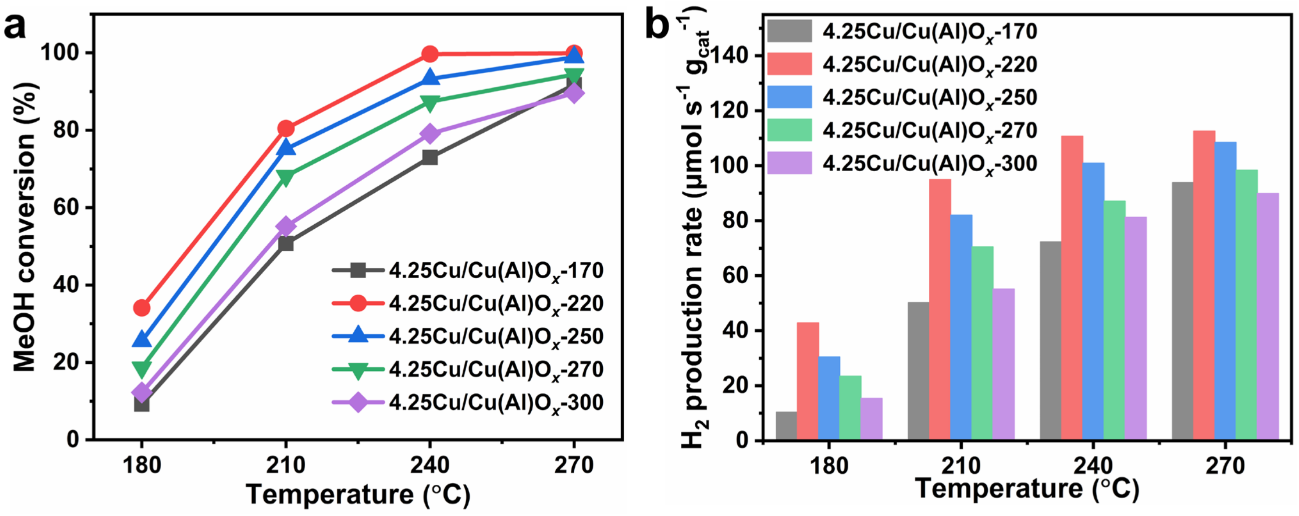

Supplementary Figure 30. Catalytic performance of various samples. a Methanol conversion and **b** H_2 production rate in the presence of 4.25CuAlO$_x$ samples reduced at 170, 220, 250, 270, 300 °C, respectively. The 4.25Cu/Cu(Al)O$_x$-220 herein and 4.25Cu/Cu(Al)O$_x$ in the manuscript refer to the same catalyst. Reaction conditions: catalyst (0.25 g) + SiO_2 (2.50 g); liquid feed of S/C = 2 at 0.040 mL min^{-1}; He carrier at 50.0 mL min^{-1}; time on stream: 1.0 h.

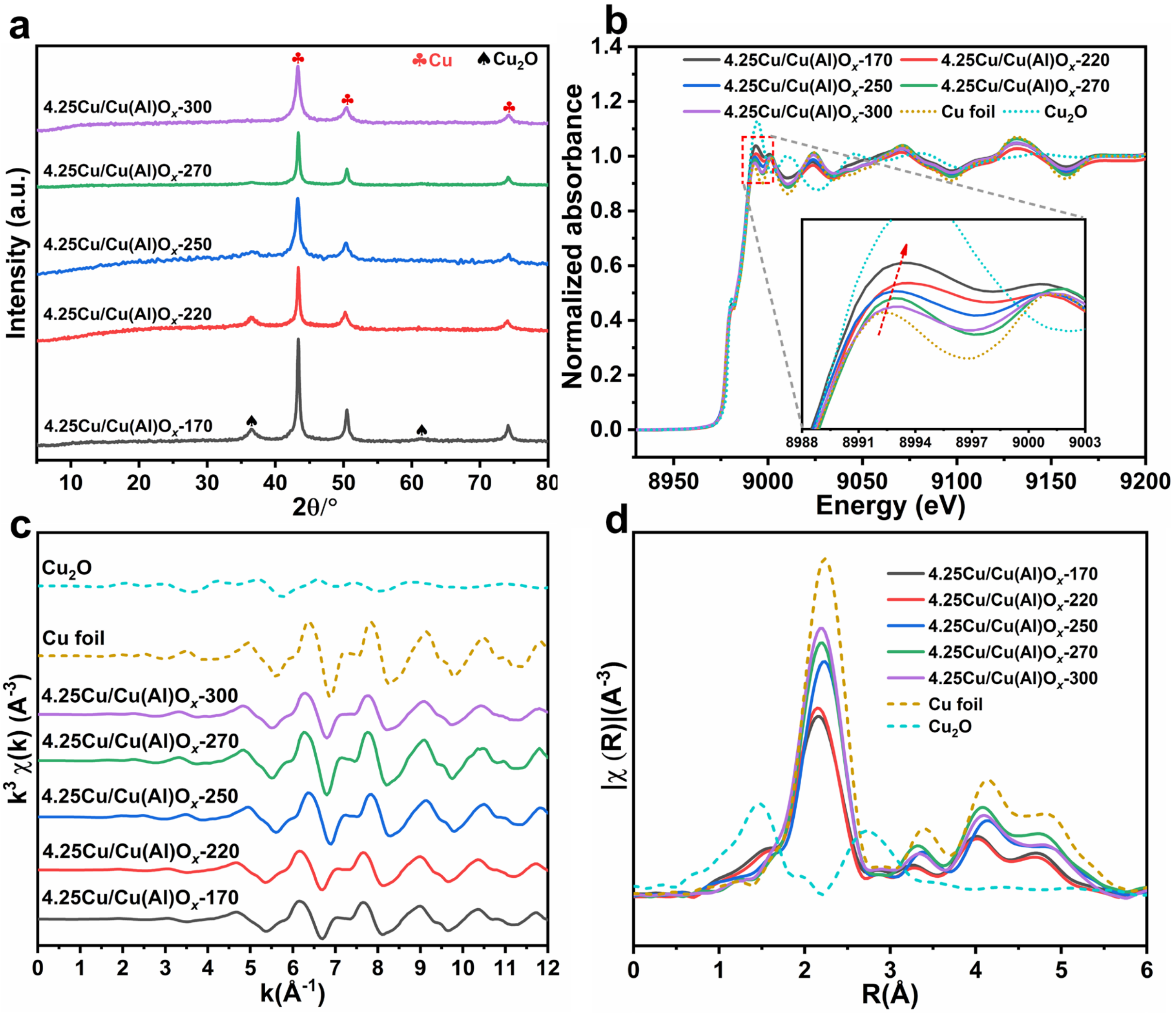

Supplementary Figure 31. Structure characterization of various samples. **a** XRD patterns, **b** XANES spectra, **c** EXAFS spectra at k-space and **d** R-space of the 4.25Cu/Cu(Al)O_x catalysts reduced at 170, 220, 250, 270 and 300 °C, respectively. The 4.25Cu/Cu(Al)O_x-220 herein and 4.25Cu/Cu(Al)O_x in the manuscript refer to the same catalyst.

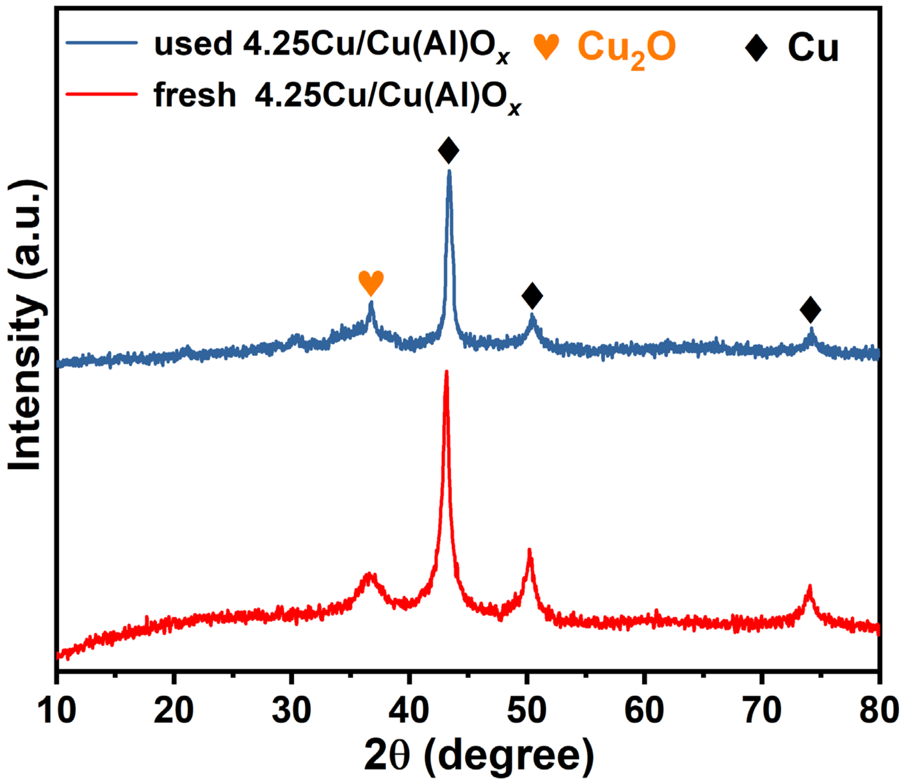

Supplementary Figure 32. Structure characterization of various samples. XRD patterns of the fresh and used 4.25Cu/Cu(Al)O_x catalyst.

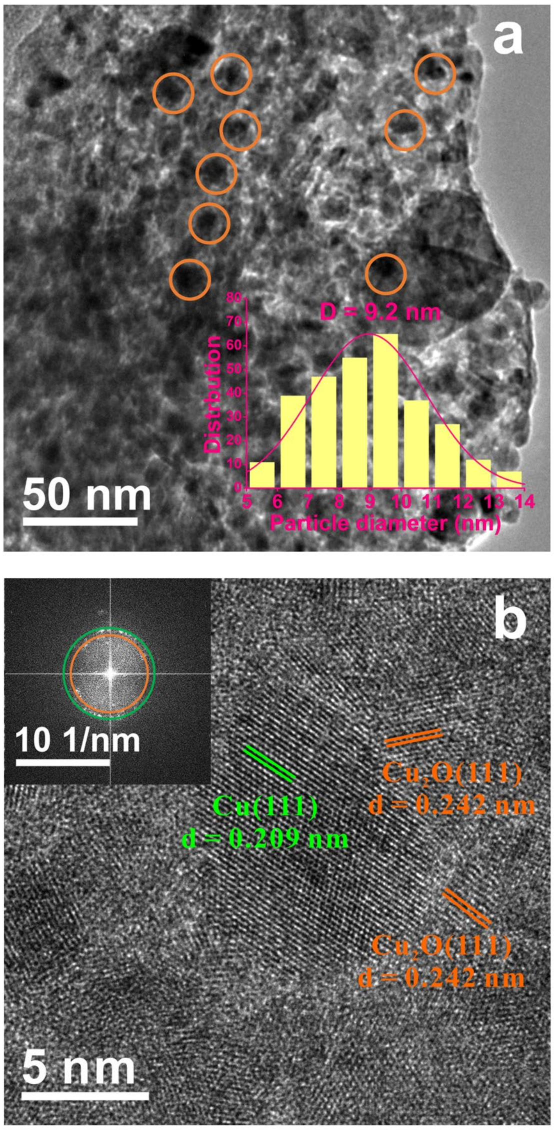

Supplementary Figure 33. Electron micrology studies on the used 4.25Cu/Cu(Al)O$_x$. a TEM image (inset: the histogram for size distribution of Cu nanoparticles) and **b** HR-TEM image (inset: fast Fourier transform (FFT) patterns of panel) of the used 4.25Cu/Cu(Al)O$_x$ catalyst.

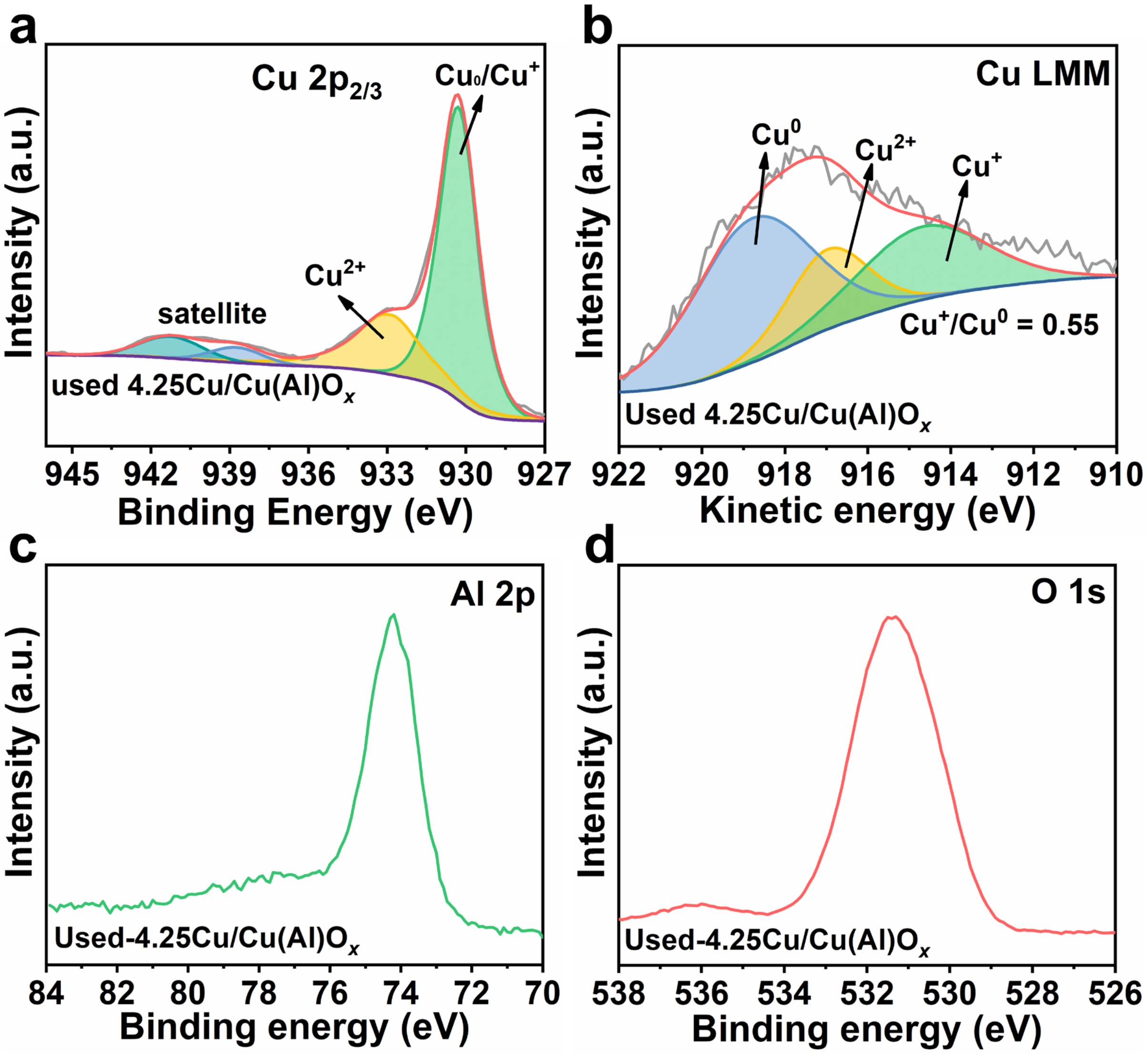

Supplementary Figure 34. XPS spectra of used 4.25Cu/Cu(Al)O*x*. XPS spectra of **a** Cu 2*p*, **b** Cu LMM, **c** Al 2*p* and **d** O 1*s* for the used 4.25Cu/Cu(Al)O*x* catalyst.

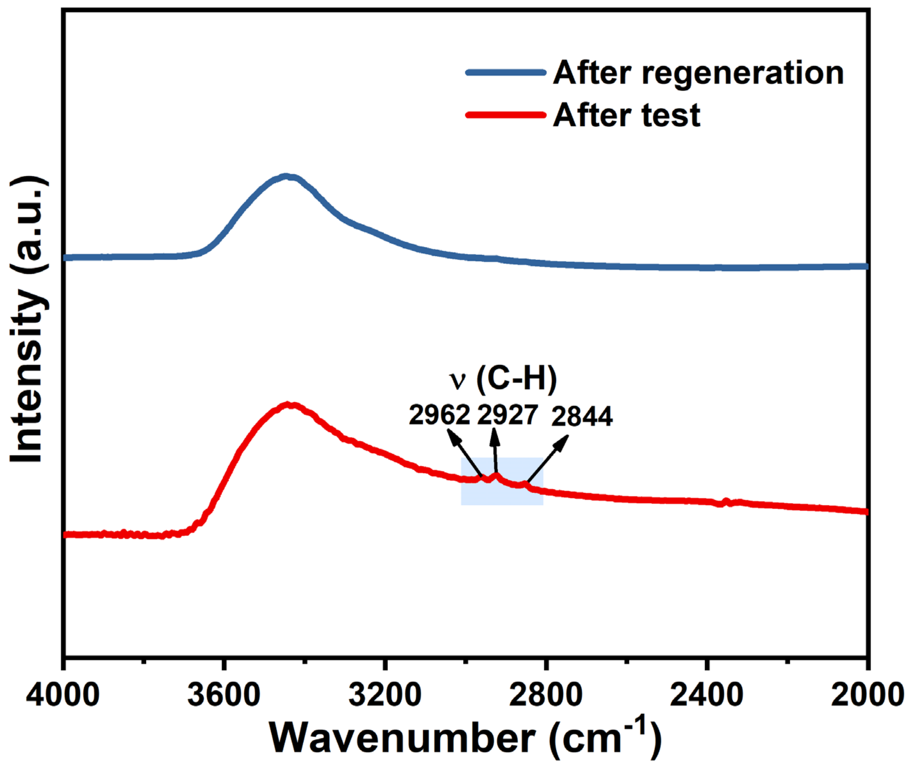

Supplementary Figure 35. FT-IR spectra of used 4.25Cu/Cu(Al)O$_x$. FT-IR spectra of the 4.25Cu/Cu(Al)O$_x$ catalyst after stability test and regeneration process, respectively.

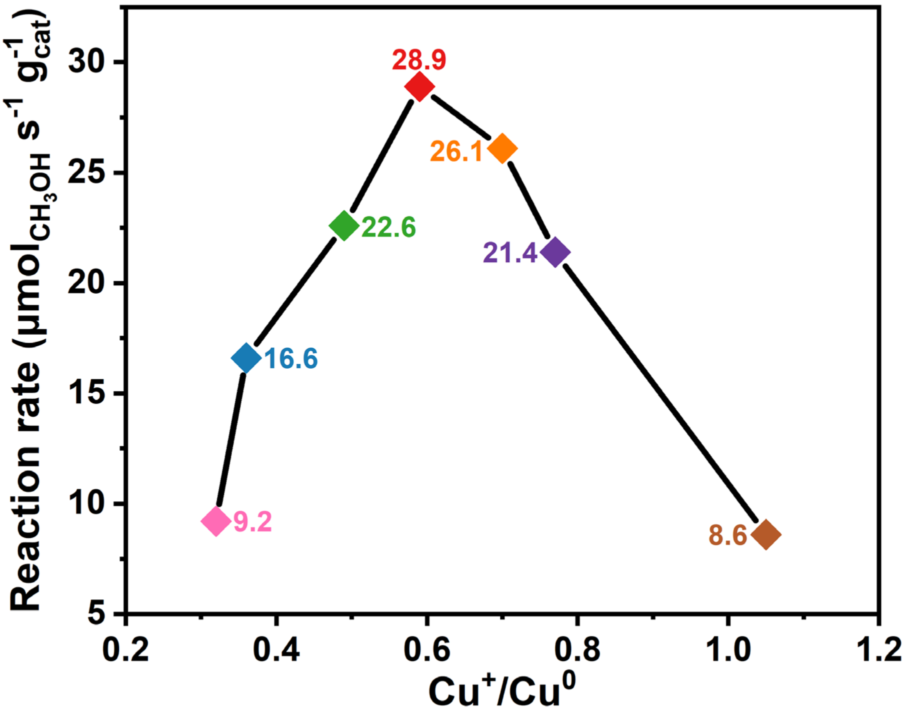

Supplementary Figure 36. Statistical analysis of data results. Reaction rate of CH_3OH *vs.* Cu^+/Cu^0 ratio based on *quasi-in situ* Cu *LMM* spectra.

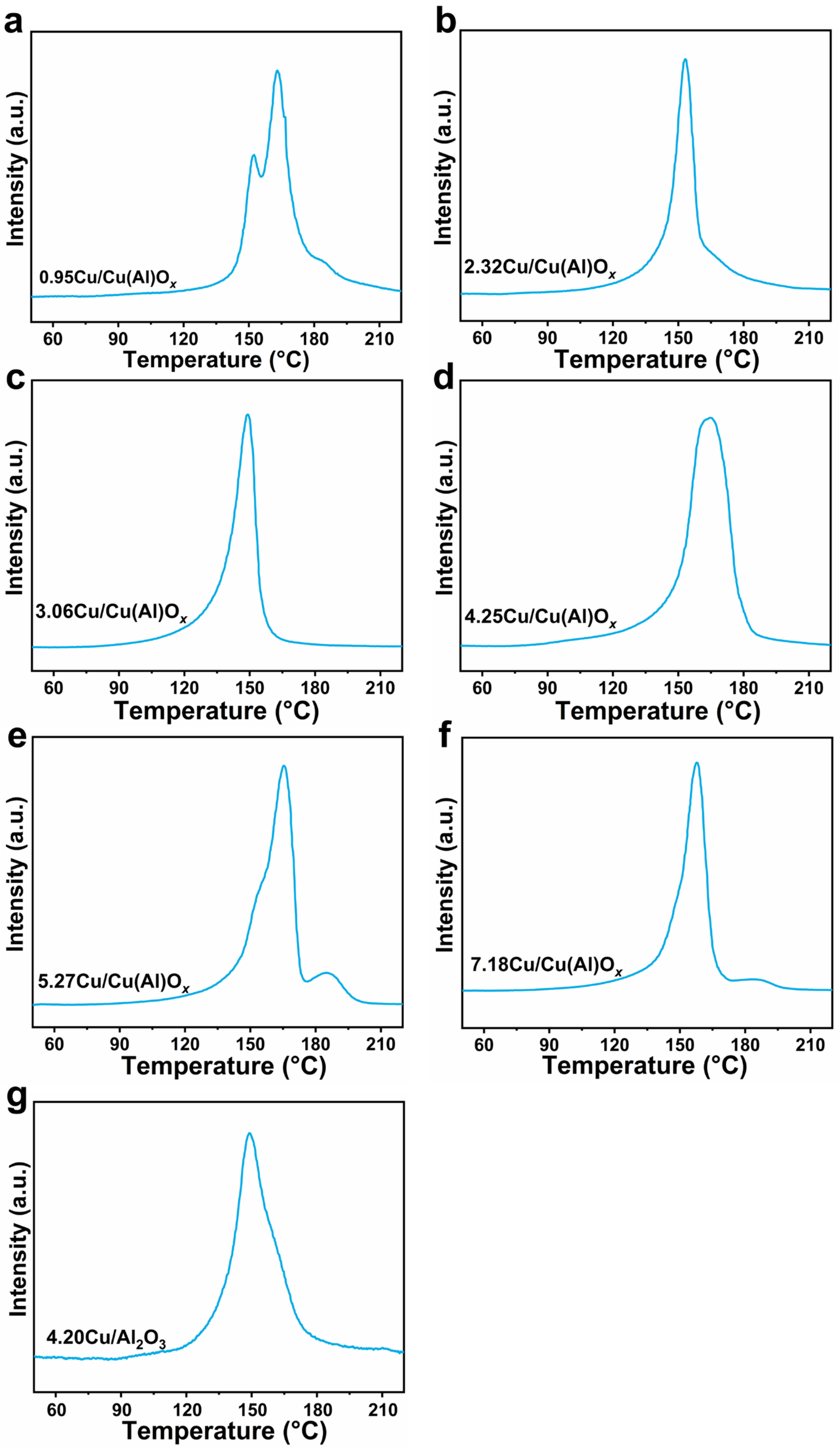

Supplementary Figure 37. N_2O-H_2 titration experiment. N_2O oxidation-H_2 reduction titration of **a** 0.95Cu/Cu(Al)O$_x$, **b** 2.32Cu/Cu(Al)O$_x$, **c** 3.06Cu/Cu(Al)O$_x$, **d** 4.25Cu/Cu(Al)O$_x$, **e** 5.27Cu/Cu(Al)O$_x$, **f** 7.18Cu/Cu(Al)O$_x$ and **g** 4.20Cu/Al$_2$O$_3$, respectively.

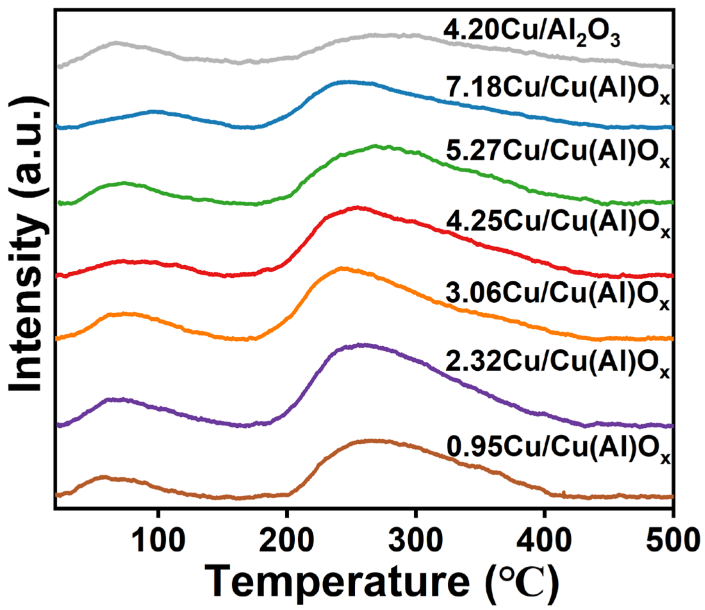

Supplementary Figure 38. CO-TPD experiment. CO-TPD curves of 0.95Cu/Cu(Al)O_x, 2.32Cu/Cu(Al)O_x, 3.06Cu/Cu(Al)O_x, 4.25Cu/Cu(Al)O_x, 5.27Cu/Cu(Al)O_x, 7.18Cu/Cu(Al)O_x and 4.20Cu/Al_2O_3 samples, respectively.

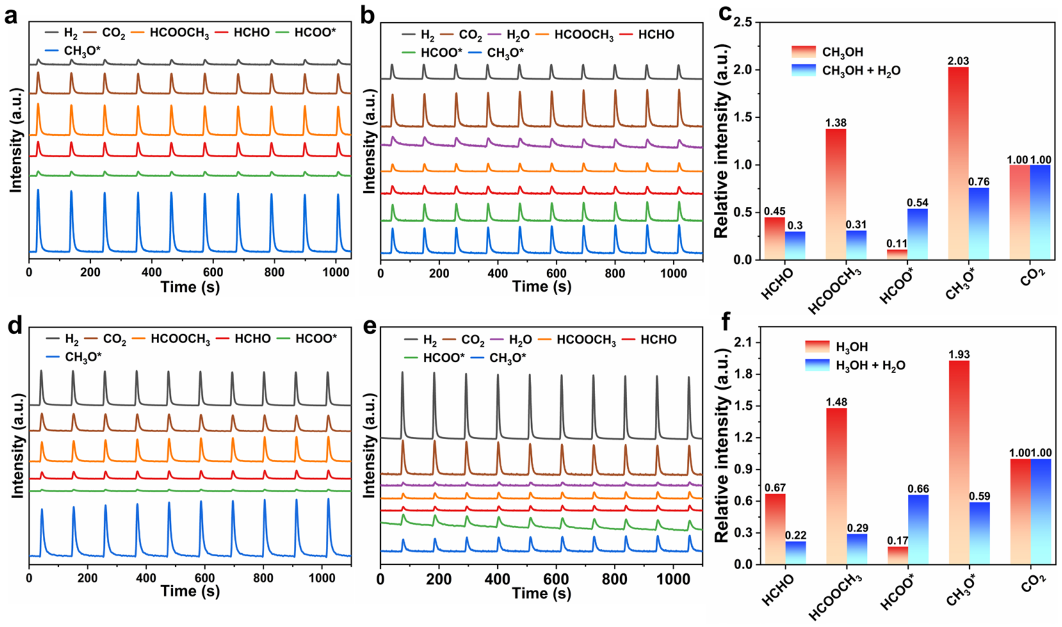

Supplementary Figure 39. Pulse experiments with mass spectral analysis. MS signals from the pulse experiments of **a**,**d** single methanol and **b**,**e** methanol-water mixed solution over **a**,**b** 0.95Cu/Cu(Al)O$_x$ and **d**,**e** 7.18Cu/Cu(Al)O$_x$ at 240 °C, respectively. Relative intensity of the reaction intermediates normalized by CO_2 signal intensity over **c** 0.95Cu/Cu(Al)O$_x$ and **f** 7.18Cu/Cu(Al)O$_x$ samples, respectively.

Supplementary Note 2

The pulse experiments of CH_3OH and H_2O were also carried out over 0.95Cu/Cu(Al)O$_x$, 4.25Cu/Cu(Al)O$_x$ and 7.18Cu/Cu(Al)O$_x$ samples, respectively. From the results in Fig. 4a,b and Supplementary Fig. 39, the signals of H_2, CH_3O*, HCOO*, HCHO, $HCOOCH_3$ and CO_2 are captured by mass spectrometer (MS) detector after pulsing CH_3OH and H_2O, which is consistent with the reaction route demonstrated by *in situ* DRIFT spectra. The results from the normalized statistics by using CO_2 signal intensity as standard are shown in Fig. 4c and Supplementary Fig. 39c,f. After the co-introduction of CH_3OH and H_2O, the 4.25Cu/Cu(Al)O$_x$ catalyst shows the lowest relative intensity of CH_3O*, HCOO*, HCHO and $HCOOCH_3$ intermediates in comparison with the 0.95Cu/Cu(Al)O$_x$ and 7.18Cu/Cu(Al)O$_x$ samples (especially for CH_3O* and HCOO*), indicating the

fastest conversion rate over 4.25Cu/Cu(Al)O_x catalyst.

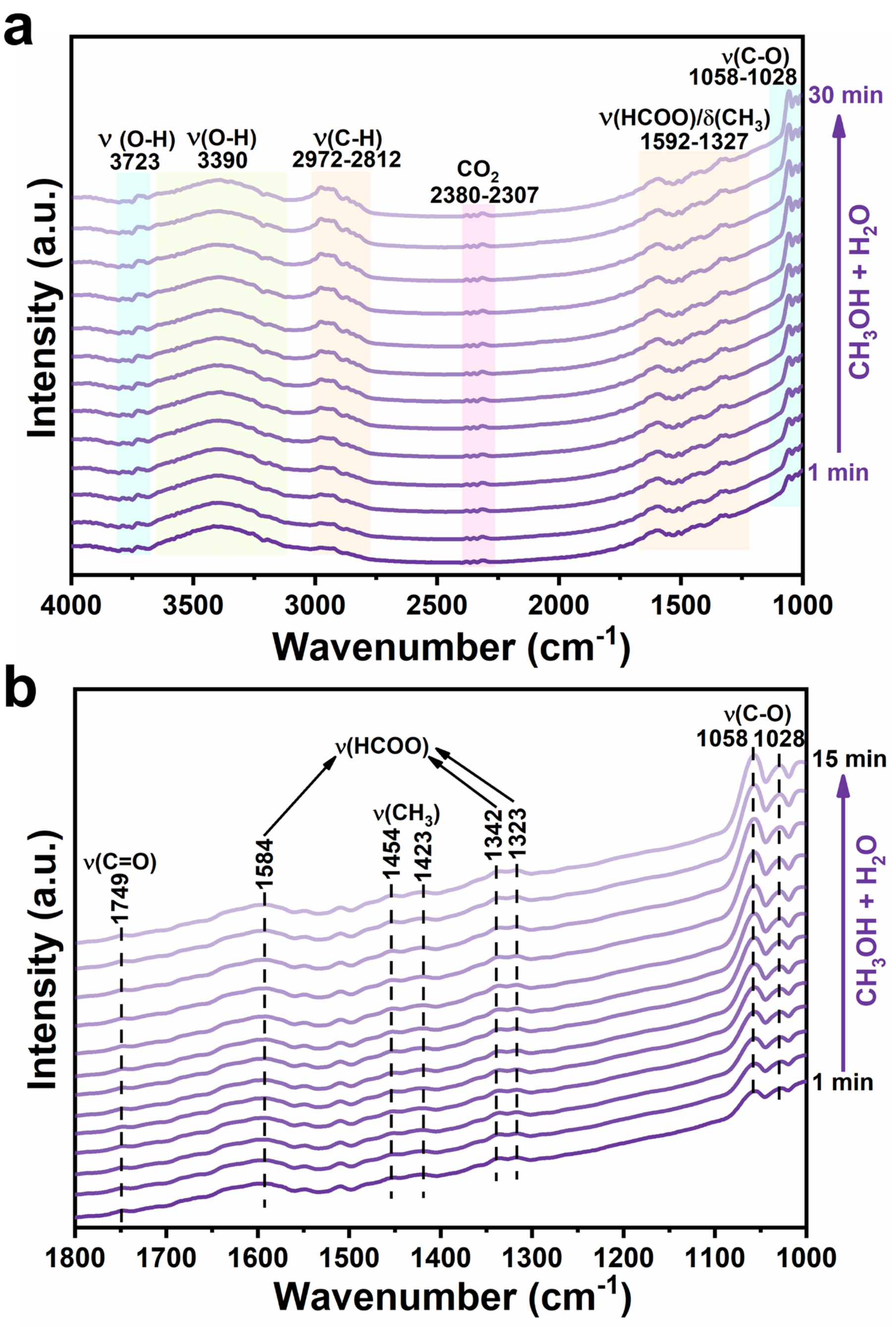

Supplementary Figure 40. *In situ* DRIFTS spectra of 4.25Cu/Cu(Al)O_x catalyst. *In situ* FT-IR spectra of 4.25Cu/Cu(Al)O_x catalyst with the introduction of $CH_3OH/H_2O/He$ at 240 °C within **a** 4000–1000 cm^{-1} and **b** 1800–1000 cm^{-1}, respectively.

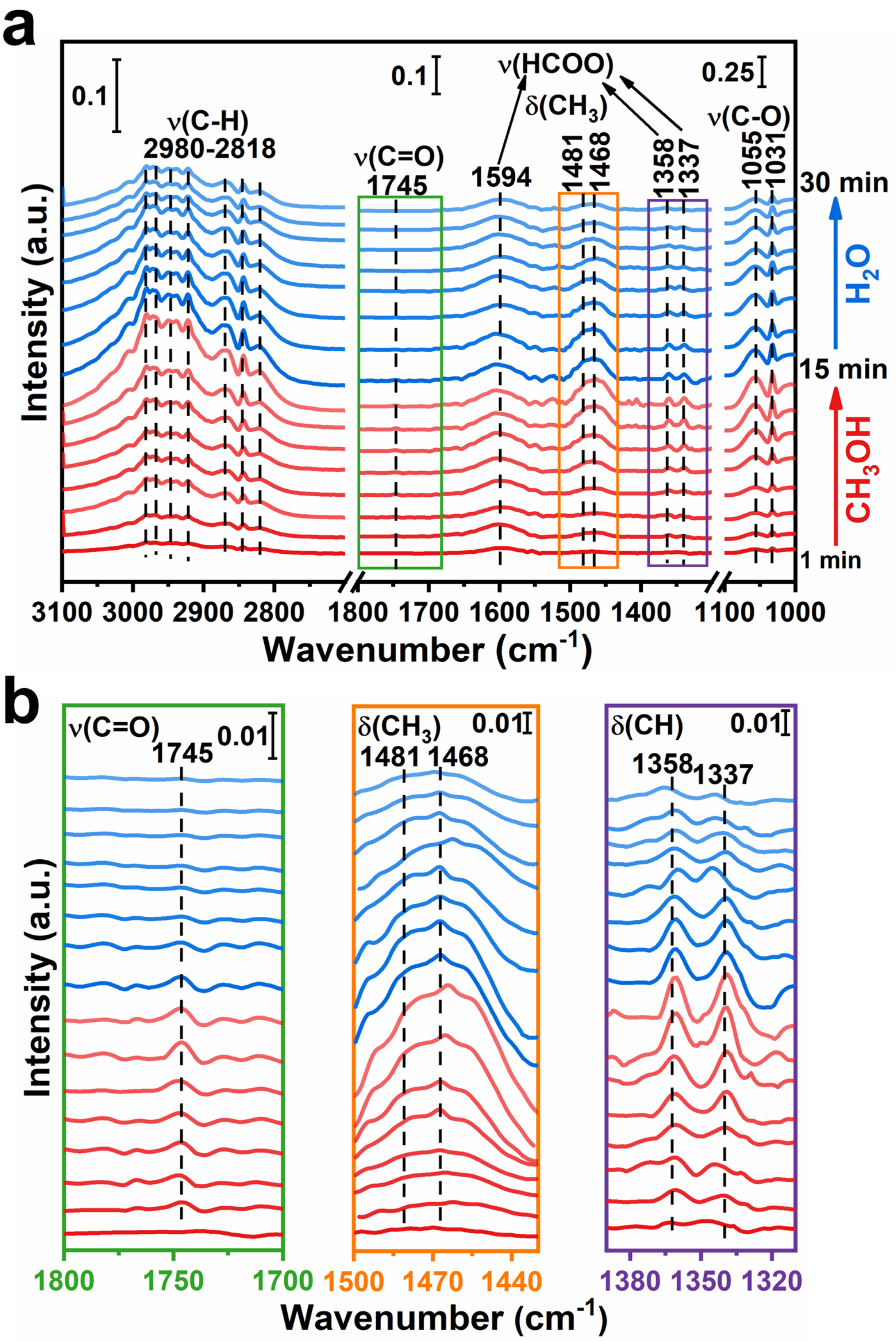

Supplementary Figure 41. *In situ* DRIFTS spectra of 0.95Cu/Cu(Al)O_x sample. *In situ* FT-IR spectra of 0.95Cu/Cu(Al)O_x along with the sequential introduction of CH_3OH/He (1−15 min) and H_2O/He (15−30 min) at 240 °C within **a** 3100–2700 cm^{-1}, 1800–1300 cm^{-1} and 1100–1000 cm^{-1}, respectively. **b** Local magnification regions in (a).

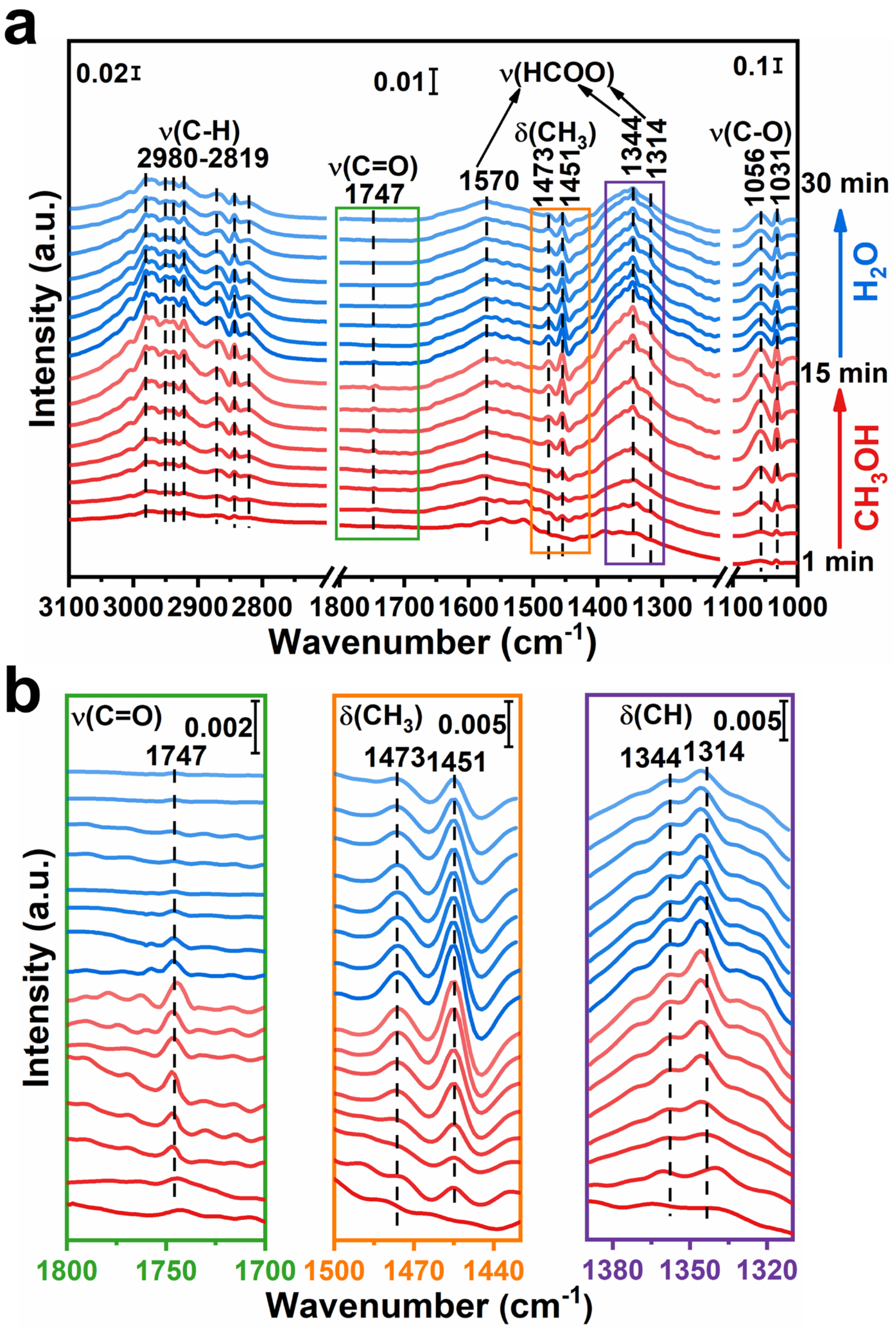

Supplementary Figure 42. *In situ* DRIFTS spectra of 7.18Cu/Cu(Al)O$_x$ sample. *In situ* FT-IR spectra of 7.18Cu/Cu(Al)O$_x$ along with the sequential introduction of CH_3OH/He (1−15 min) and H_2O/He (15–30 min) at 240 °C within **a** 3100–2700 cm^{-1}, 1800–1300 cm^{-1} and 1100–1000 cm^{-1}, respectively. **b** Local magnification regions in (a).

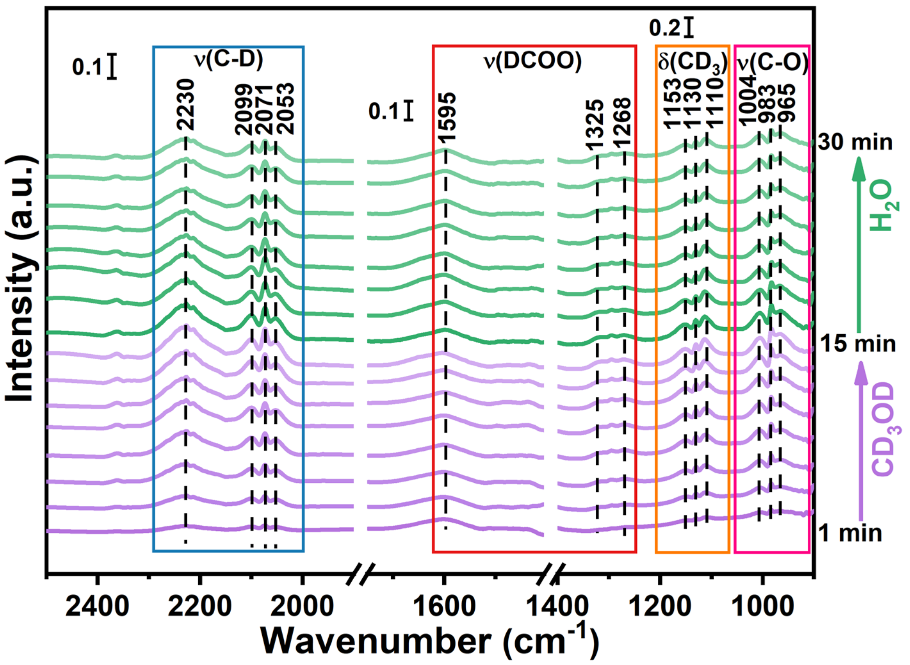

Supplementary Figure 43. ***In situ*** **DRIFTS spectra of 0.95Cu/Cu(Al)O_x catalyst.** *In situ* FT-IR spectra of 0.95Cu/Cu(Al)O_x along with adsorption of CD_3OD/He (1–15 min) and subsequent switching to H_2O/He (15–30 min) at 240 °C.

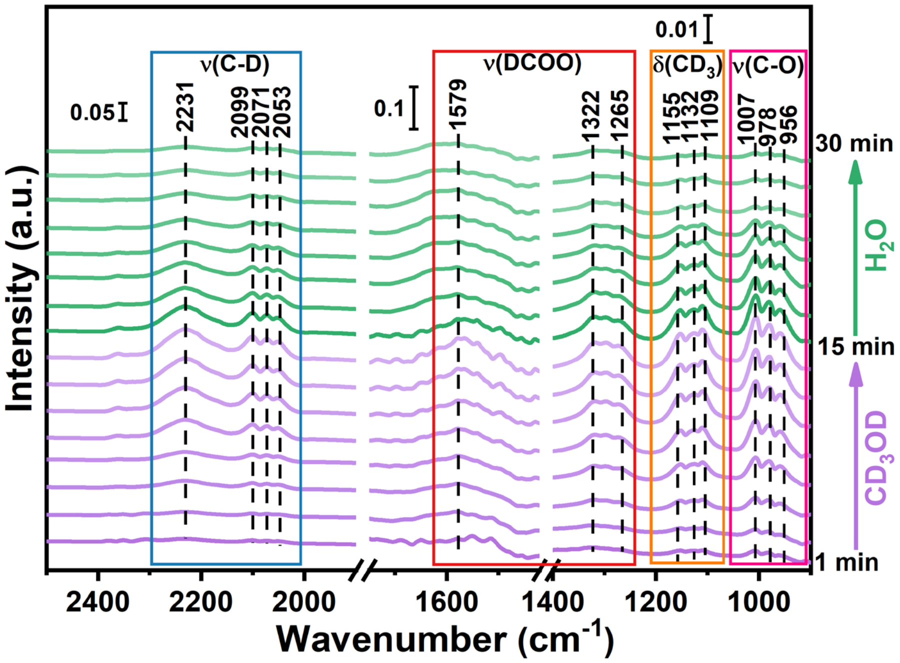

Supplementary Figure 44. ***In situ*** **DRIFTS spectra of 4.25Cu/Cu(Al)O$_x$ catalyst.** *In situ* FT-IR spectra of 4.25Cu/Cu(Al)O$_x$ along with adsorption of CD_3OD/He (1−15 min) and subsequent switching to H_2O/He (15−30 min) at 240 °C.

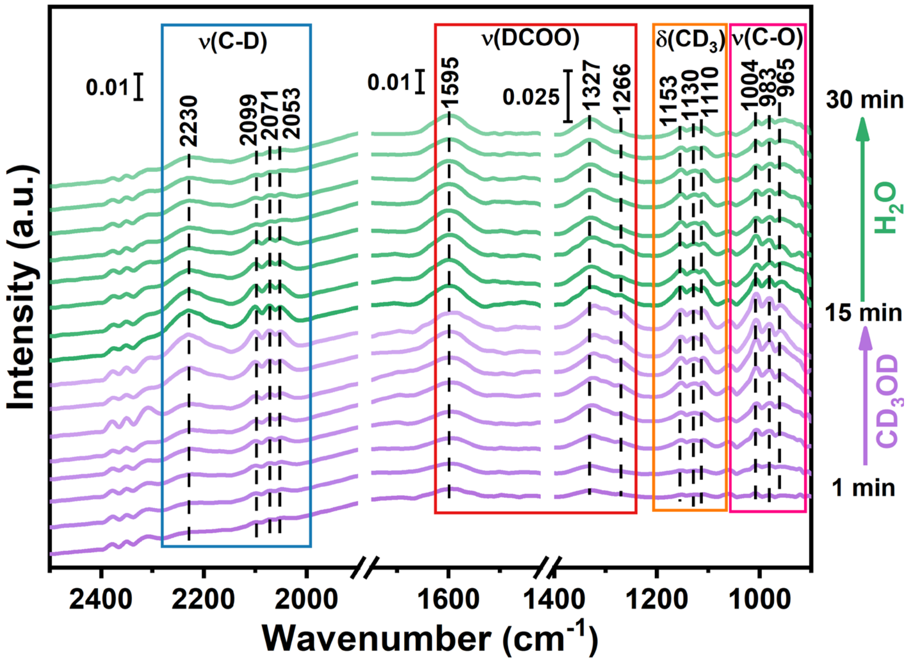

Supplementary Figure 45. *In situ* DRIFTS spectra of 7.18Cu/Cu(Al)O_x catalyst. *In situ* FT-IR spectra of 7.18Cu/Cu(Al)O_x along with adsorption of CD_3OD/He (1–15 min) and subsequent switching to H_2O/He (15–30 min) at 240 °C.

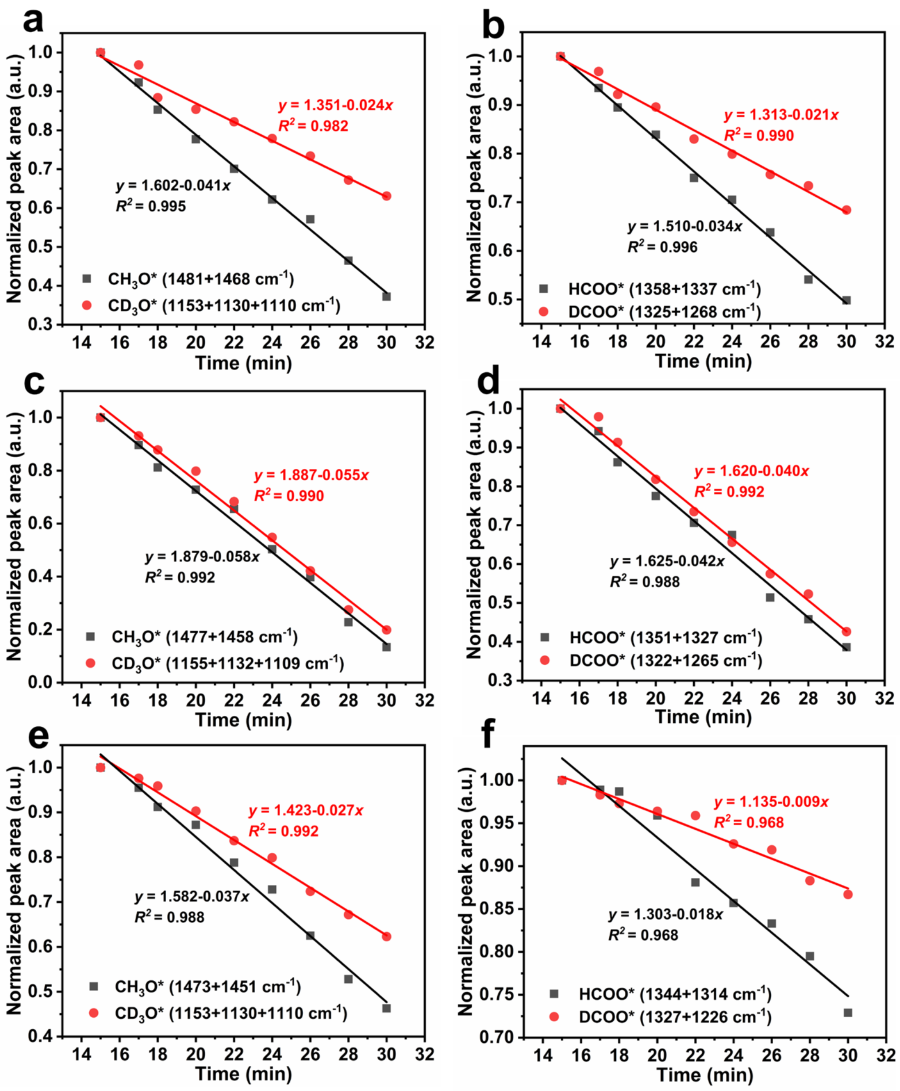

Supplementary Figure 46. Statistical results of *in situ* DRIFT spectra. Linear fitting results from normalized peak area of C–H and C–D bonds in **a,c,e** CH_3O^*/CD_3O^* ratio and **b,d,f** HCOO*/DCOO* ratio *vs*. ventilation time of water (within 15–30 min) for the sample of **a,b** 0.95Cu/Cu(Al)O_x, **c,d** 4.25Cu/Cu(Al)O_x and **e,f** 7.18Cu/Cu(Al)O_x. Data are obtained from Fig. 5b and Supplementary Figs. 44–48, respectively (the slope represents the relative consumption rate).

Supplementary Note 3

Supplementary Figs. 43–45 shows *in situ* FT-IR spectra of 0.95Cu/Cu(Al)O_x, 4.25Cu/Cu(Al)O_x and 7.18Cu/Cu(Al)O_x catalysts along with adsorption of CD_3OD/He (1−15 min) and subsequent switching to H_2O/He (15–30 min) at 240 °C. For the adsorption of CD_3OD, due to the isotopic effect,

the vibration frequency of C–D bonds moves towards a lower wavenumber relative to C–H bonds. The band at 2000–2300 cm^{-1} is attributed to the stretching vibration of C–D bonds; the ones at 1268, 1325 and 1595 cm^{-1} is assigned to the bending vibration of C–D bonds and DCOO* species, respectively[9,10]. The peaks at 1110, 1130 and 1153 cm^{-1} are related to the bending vibration of C–D bonds in CD_3O* group[11,12]. Based on *in situ* FT-IR results (Fig. 5b and Supplementary Figs. 41–45), the correlation between normalized peak area of C–H bonds in CH_3O* and HCOO* as well as C–D bonds in CD_3O* and DCOO* versus ventilation time of saturated water vapor (within 15–30 min) was established, respectively (Supplementary Fig. 46). Compared with corresponding non-deuterium species, the relative consumption rates decrease from 0.041 (R_{H_m}) and 0.034 (R_{H_f}) to 0.024 (R_{D_m}) and 0.021 (R_{D_f}) in the presence of 0.95Cu/Cu(Al)O_x catalyst, respectively (Supplementary Fig. 46a,b). For the 7.18Cu/Cu(Al)O_x catalyst, the relative consumption rates decrease from 0.037 (R_{H_m}) and 0.018 (R_{H_f}) to 0.027 (R_{D_m}) and 0.009 (R_{D_f}), respectively (Supplementary Figs. 46e,f). Remarkably, in the case of 4.25Cu/Cu(Al)O_x catalyst, the values merely show a slight decrease from 0.058 (R_{H_m}) and 0.042 (R_{H_f}) to 0.055 (R_{D_m}) and 0.040 (R_{D_f}) (Supplementary Figs. 46c,d). The slight isotopic effect in the transformation of CD_3O* and DCOO* indicates that the C–D bonds breakage is significantly promoted on the surface of 4.25Cu/Cu(Al)O_x catalyst. The results agree well with the catalytic evaluations and kinetics studies.

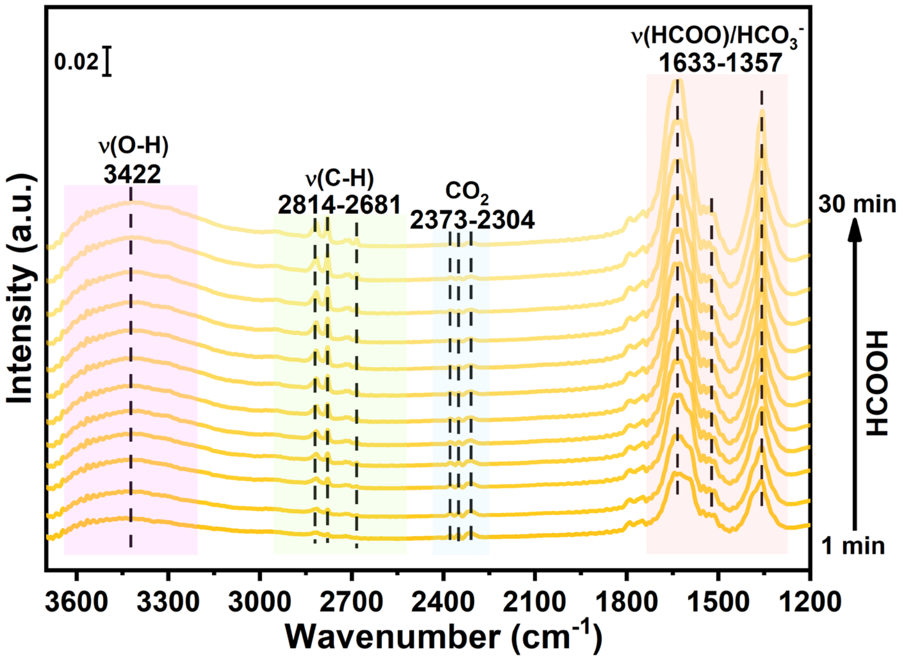

Supplementary Figure 47. *In situ* DRIFTS spectra of 0.95Cu/Cu(Al)O_x catalyst. *In situ* DRIFTS spectra of 0.95Cu/Cu(Al)O_x catalyst for the adsorption of HCOOH/He at 180 °C within 30 min.

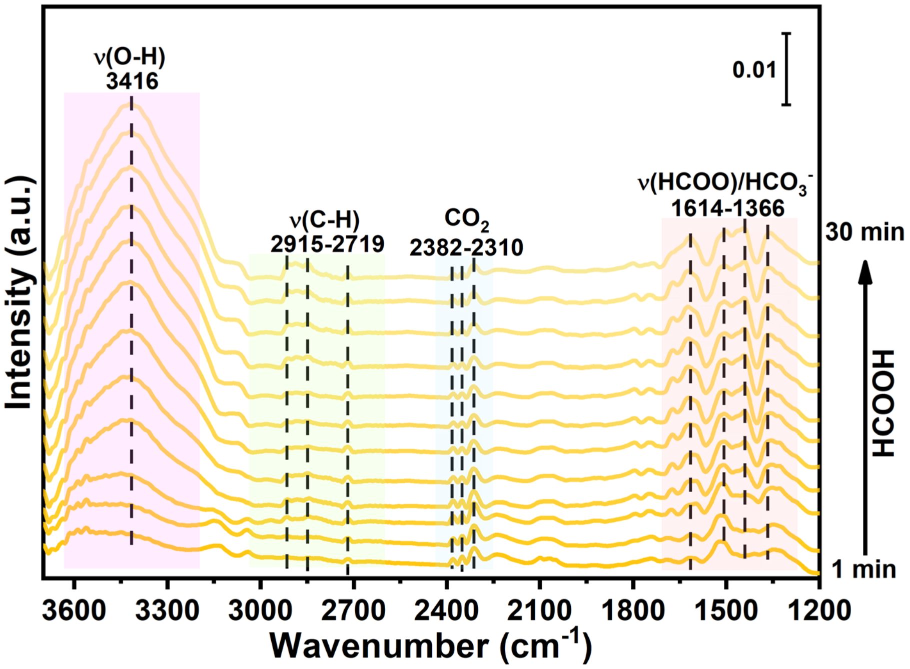

Supplementary Figure 48. ***In situ*** **DRIFTS spectra of 7.18Cu/Cu(Al)O_x catalyst.** *In situ* DRIFTS spectra of 7.18Cu/Cu(Al)O_x catalyst for the adsorption of HCOOH/He at 180 °C within 30 min.

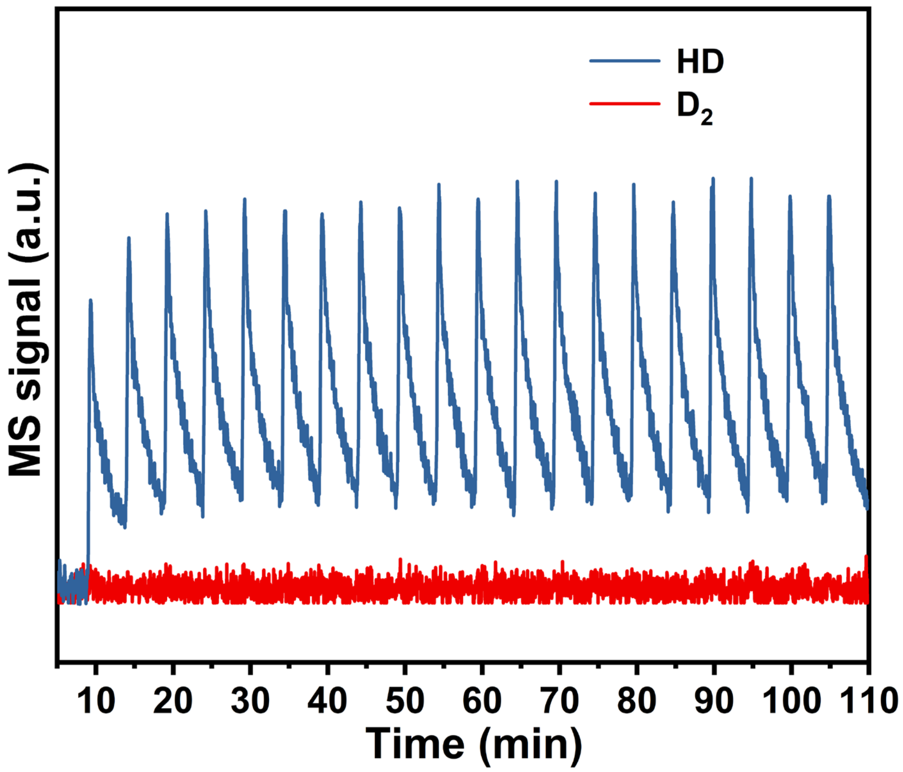

Supplementary Figure 49. H_2/D_2 exchange experiment. H_2/D_2 exchange experiment over the 4.25Cu/Cu(Al)O_x catalyst.

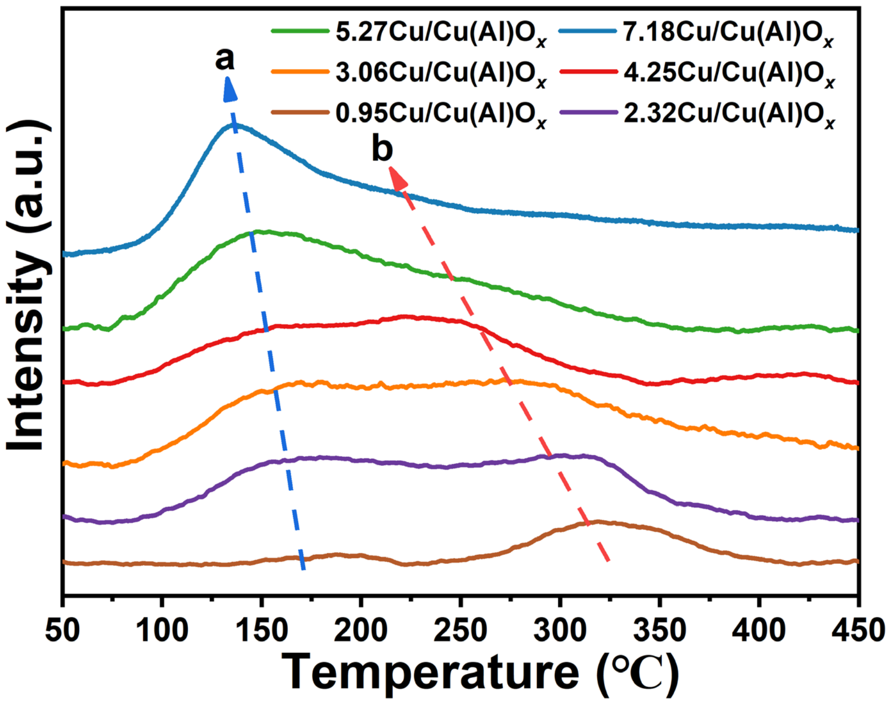

Supplementary Figure 50. H_2-TPD measurements. H_2-TPD curves of *y*Cu/Cu(Al)O_x and 4.20Cu/Al_2O_3 samples (a and b denote the H_2 desorption peaks at the lower and higher temperature, respectively).

Supplementary Note 4

In order to study the hydrogen dissociation and desorption capacity of the 4.25Cu/Cu(Al)O_x catalyst, the H_2/D_2 exchange experiment (Supplementary Fig. 49) was carried out. Remarkably, D_2 molecule undergoes dissociation and binds with H atom to generate HD during consecutive pulses, indicating that the detachment of H from the catalyst surface is facile. As shown in H_2-TPD curves (Supplementary Fig. 50), the temperature of H_2 desorption peak decreases along with the increase of Cu^0/Cu^+ ratio on the catalyst surface (from 0.95Cu/Cu(Al)O_x to 7.18Cu/Cu(Al)O_x), due to the strong dehydrogenation capacity of Cu^0 species. The results demonstrate that Cu^0 serves as the binding site of H species, promoting the desorption of the H_2.

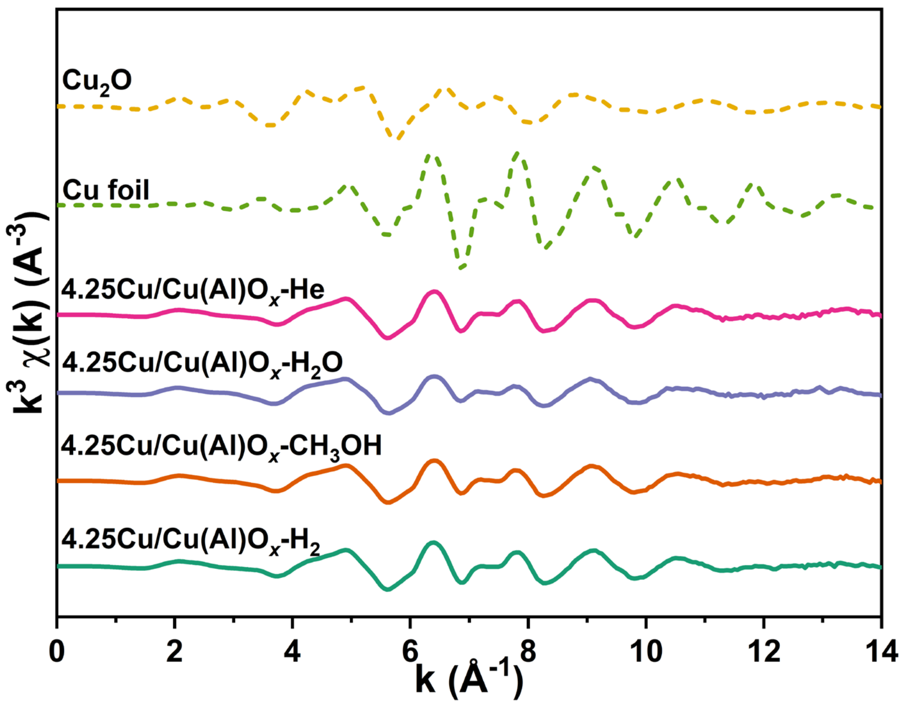

Supplementary Figure 51. Fine-structure characterization of various samples. *In situ* EXAFS spectra at k-space of Cu K-edge for the 4.25Cu/Cu(Al)O_x catalyst after H_2 reducing, CH_3OH pumping, H_2O pumping and He purging in turn.

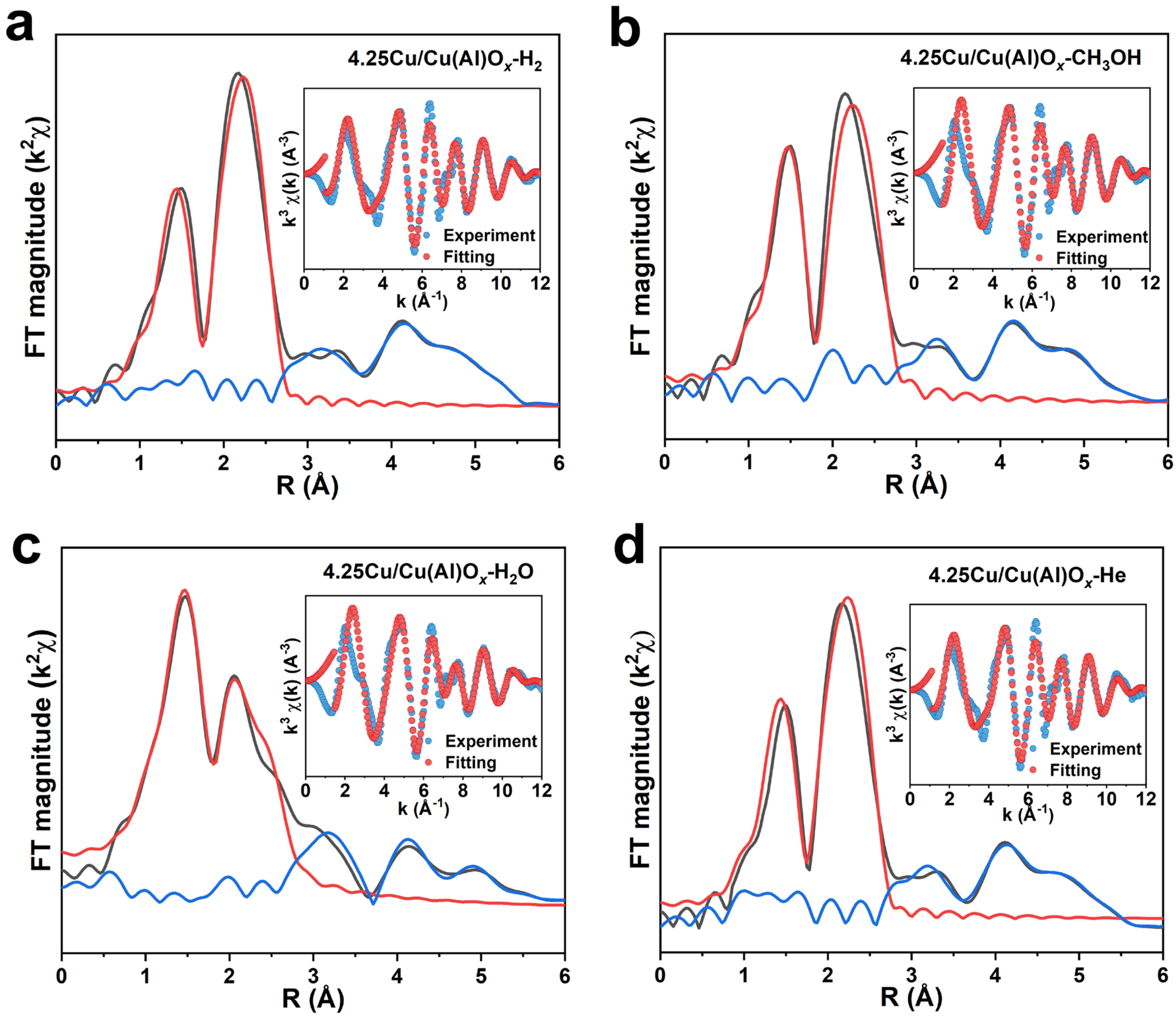

Supplementary Figure 52. *In situ* XAFS analysis of 4.25Cu/Cu(Al)O$_x$. Fitting results of *in situ* fourier-transform EXAFS spectra at Cu K-edge for the 4.25Cu/Cu(Al)O$_x$ catalyst after **a** H_2 reducing, **b** CH_3OH pumping, **c** H_2O pumping and **d** He purging in turn (the black line: experimental data; the red line: fitting curve; the blue line: residual data).

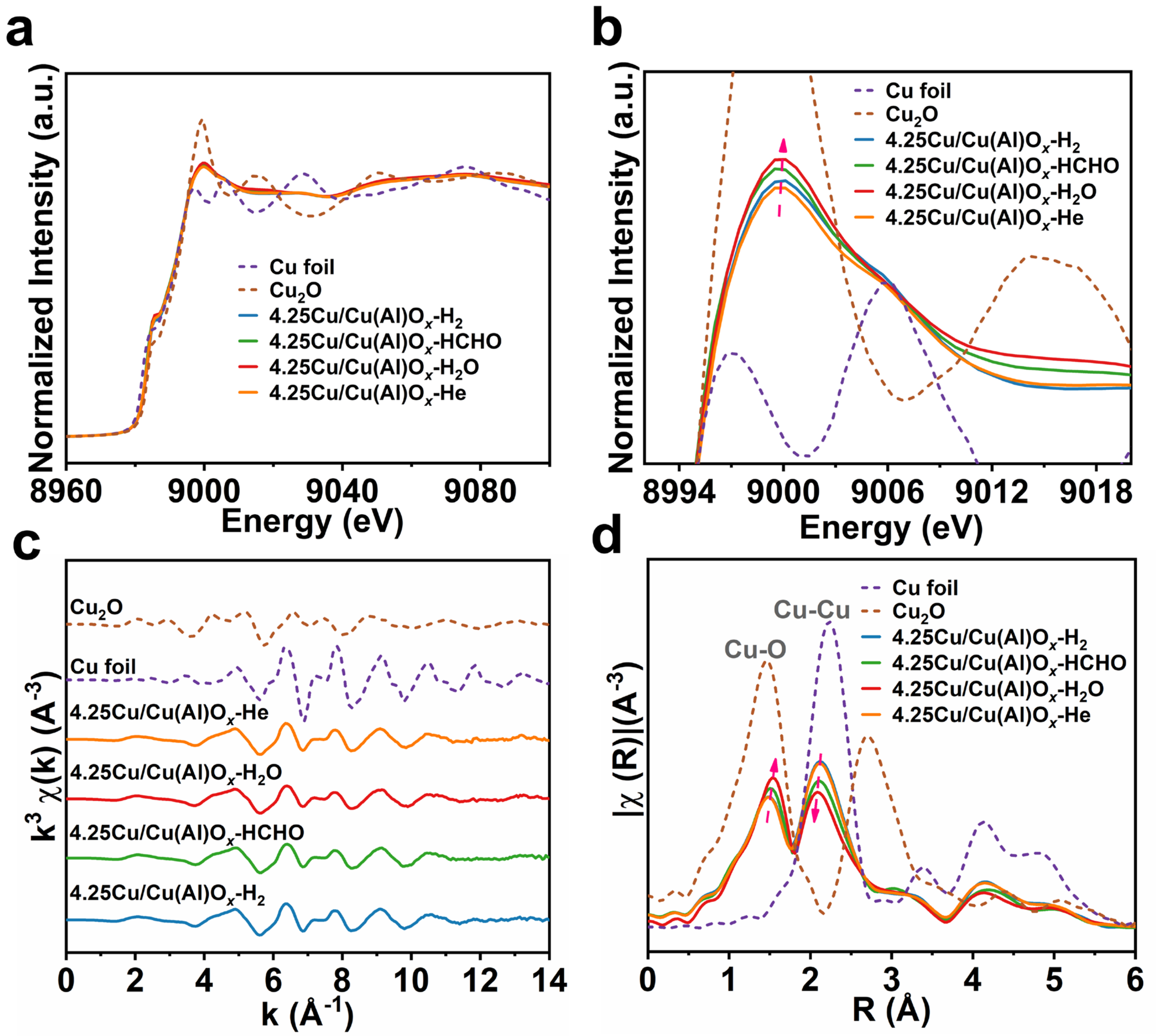

Supplementary Figure 53. *In situ* XAFS analysis of 4.25Cu/Cu(Al)O_x. a,**b** *In situ* XANES spectra, *in situ* EXAFS spectra at **c** k-space and **d** R-space of Cu K-edge for the 4.25Cu/Cu(Al)O_x catalyst after H_2 reducing, HCHO pumping, H_2O pumping and He purging in turn.

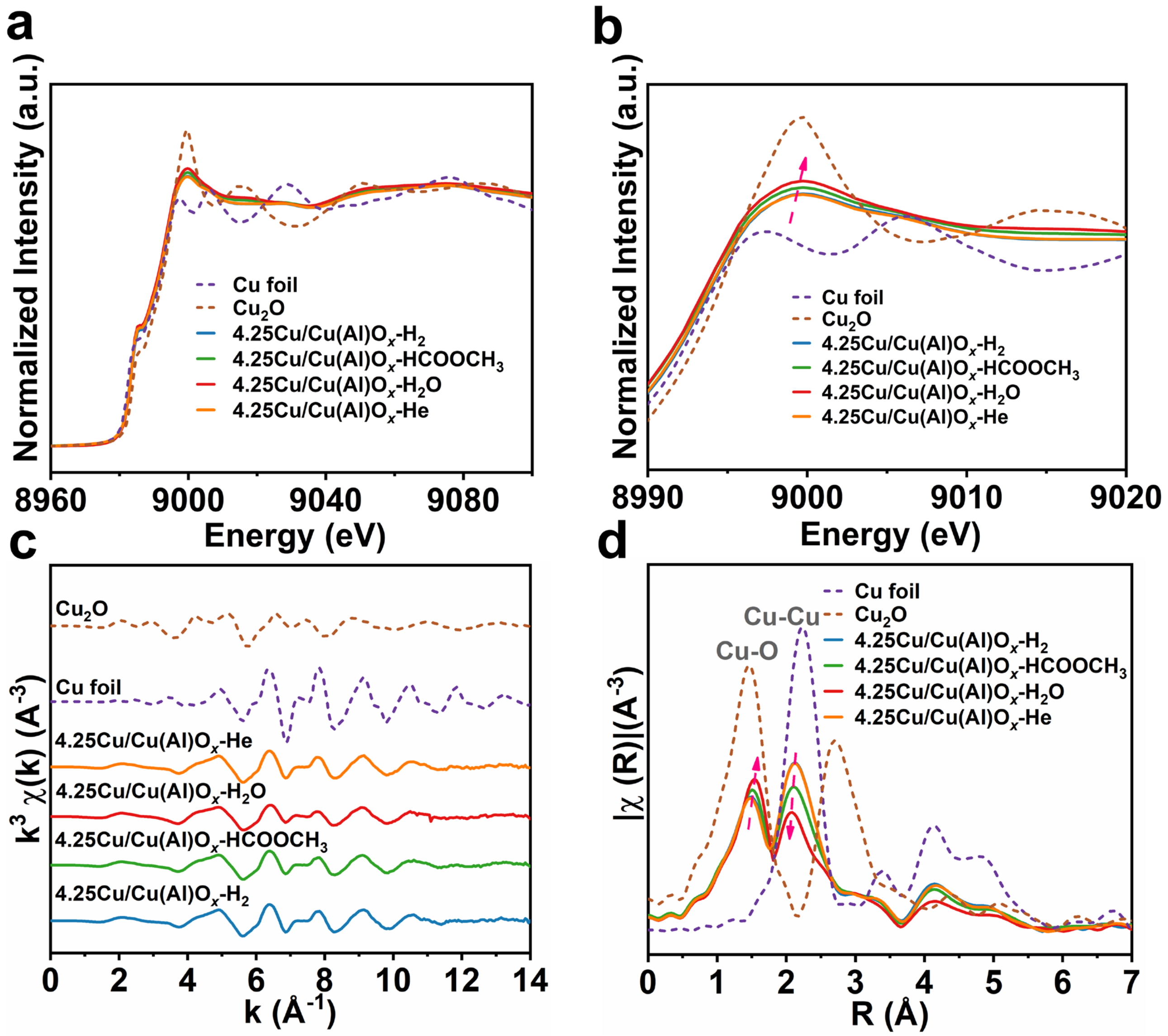

Supplementary Figure 54. *In situ* XAFS analysis of 4.25Cu/Cu(Al)O*x*. a,**b** *In situ* XANES spectra, *in situ* EXAFS spectra at **c** k-space and **d** R-space of Cu K-edge for the 4.25Cu/Cu(Al)O_x catalyst after H_2 reducing, $HCOOCH_3$ pumping, H_2O pumping and He purging in turn.

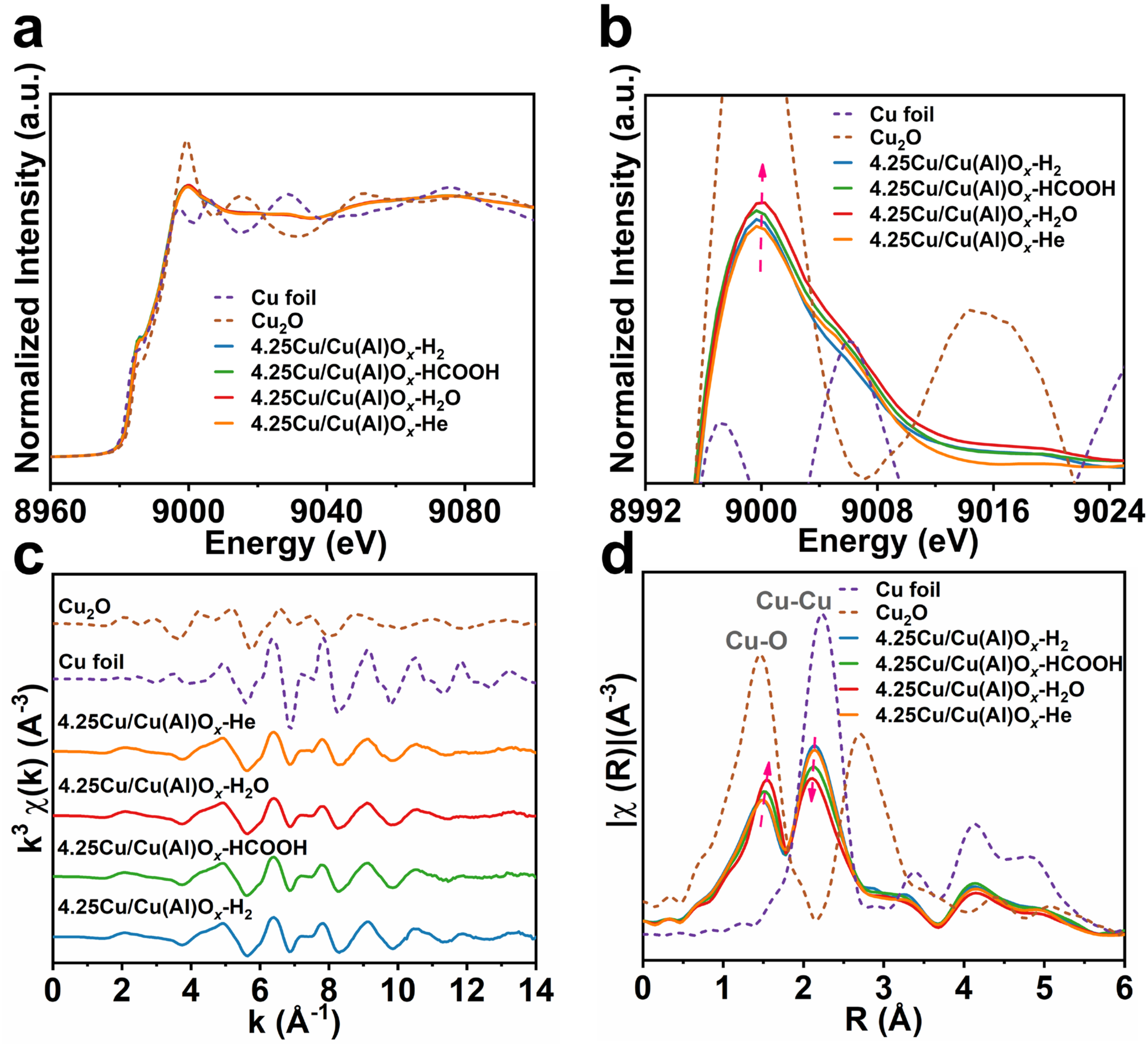

Supplementary Figure 55. *In situ* XAFS analysis of 4.25Cu/Cu(Al)O_x. a,b *In situ* XANES spectra, *in situ* EXAFS spectra at **c** k-space and **d** R-space of Cu K-edge for the 4.25Cu/Cu(Al)O_x catalyst after H_2 reducing, HCOOH pumping, H_2O pumping and He purging in turn.

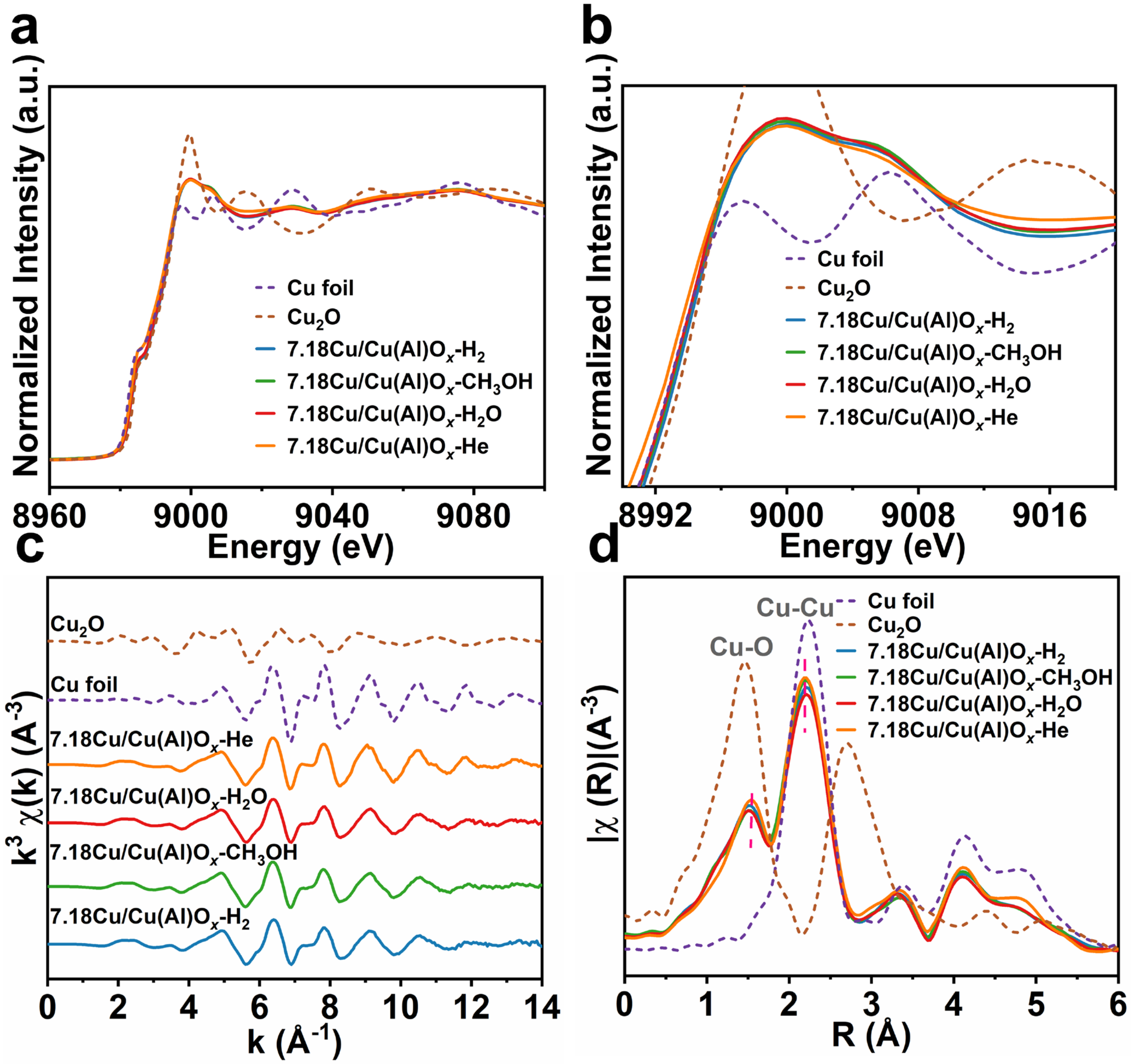

Supplementary Figure 56. *In situ* XAFS analysis of 7.18Cu/Cu(Al)O_x. a,**b** *In situ* XANES spectra, *in situ* EXAFS spectra at **c** k-space and **d** R-space of Cu K-edge for the 7.18Cu/Cu(Al)O_x catalyst after H_2 reducing, CH_3OH pumping, H_2O pumping and He purging in turn.

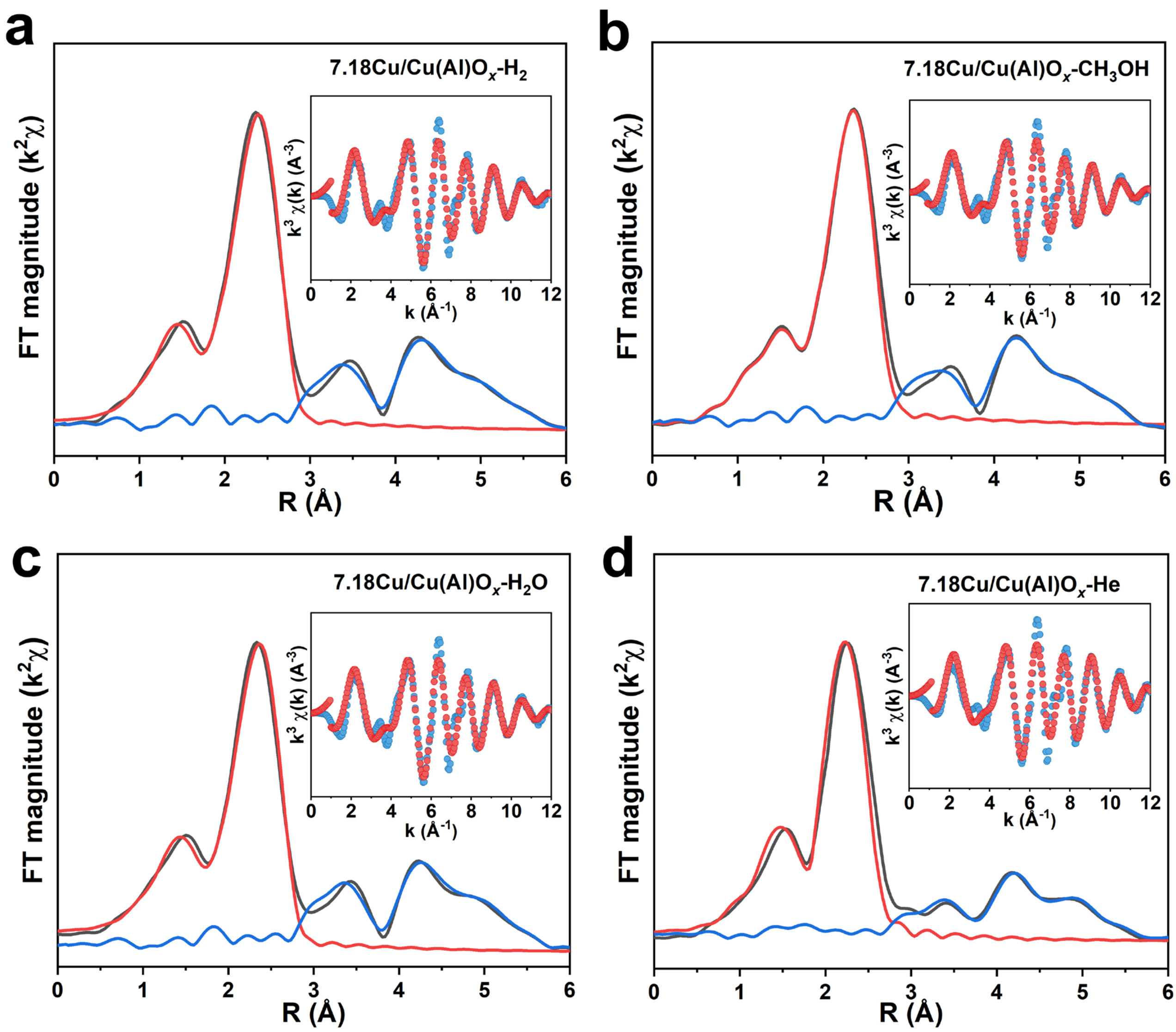

Supplementary Figure 57. *In situ* XAFS analysis of 7.18Cu/Cu(Al)O_x. Fitting results of *in situ* fourier-transform EXAFS spectra at Cu K-edge for the 7.18Cu/Cu(Al)O_x catalyst after **a** H_2 reducing, **b** CH_3OH pumping, **c** H_2O pumping and **d** He purging in turn (the black line: experimental data; the red line: fitting curve; the blue line: residual data).

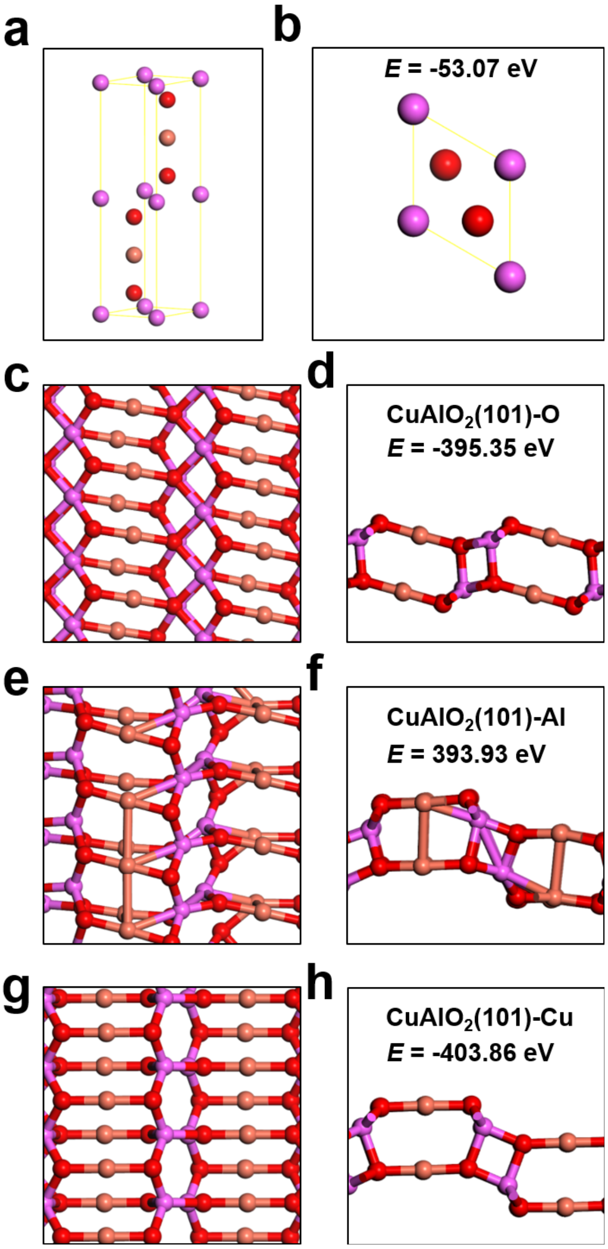

Supplementary Figure 58. DFT calculation models of $CuAlO_2$(101). a The unit cell and **b** (101) crystal facet of optimized $CuAlO_2$ structure model. The supercell (1×4×1) of $CuAlO_2$(101) with **c**,**d** the oxygen atoms, **e**,**f** aluminium atoms and **g**,**h** copper atoms exposed (red, orange and purple balls represent O, Cu and Al atoms, respectively; *E* is the structural stability energy).

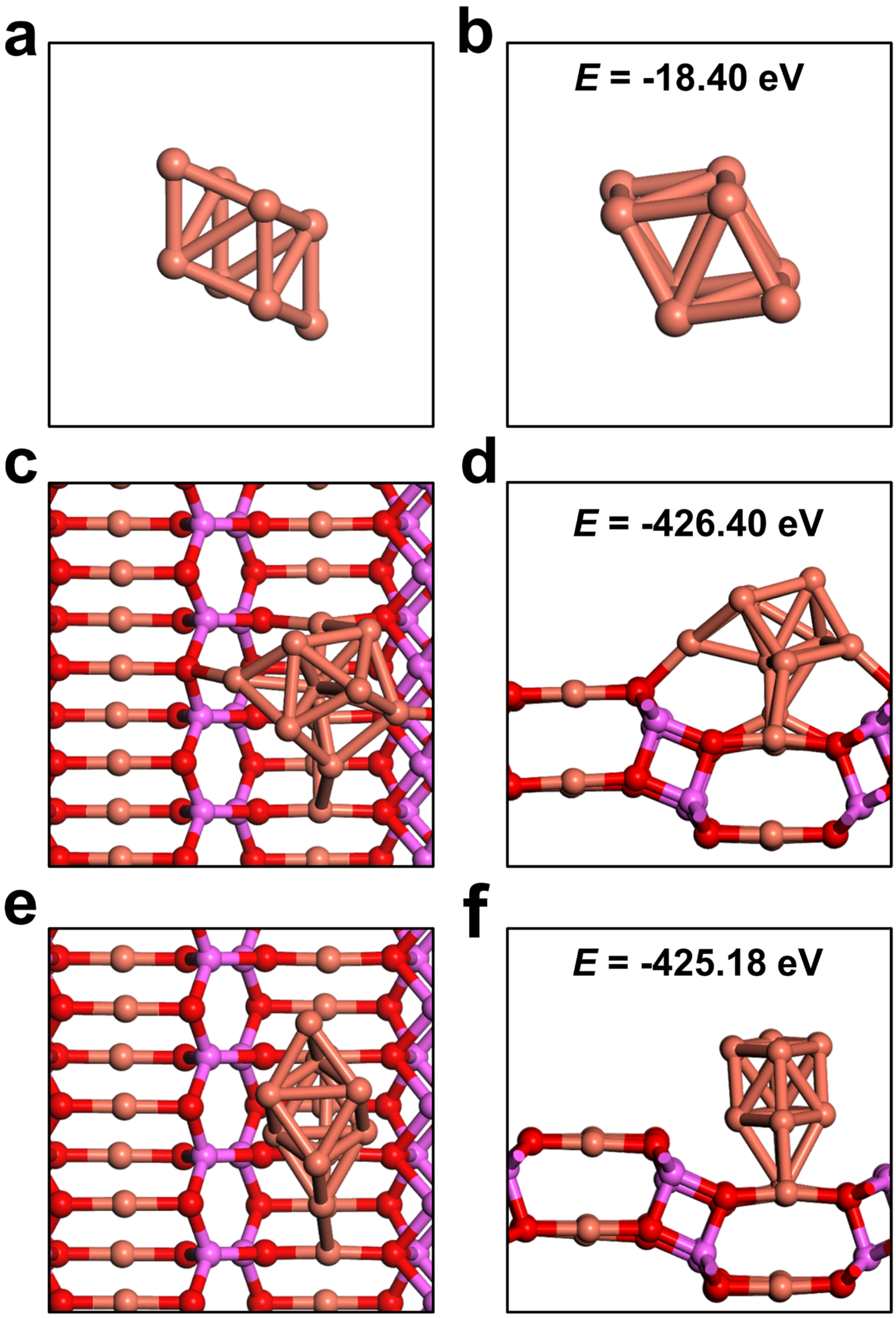

Supplementary Figure 59. DFT calculation models of Cu(111)/$CuAlO_2$(101). The Cu cluster with 8 atoms shown in **a** vertical and **b** horizontal orientations. The Cu cluster located **c**,**d** vertically and **e**,**f** horizontally on the surface of $CuAlO_2$(101), respectively (red, orange and purple balls represent O, Cu and Al atoms, respectively; *E* is the structural stability energy).

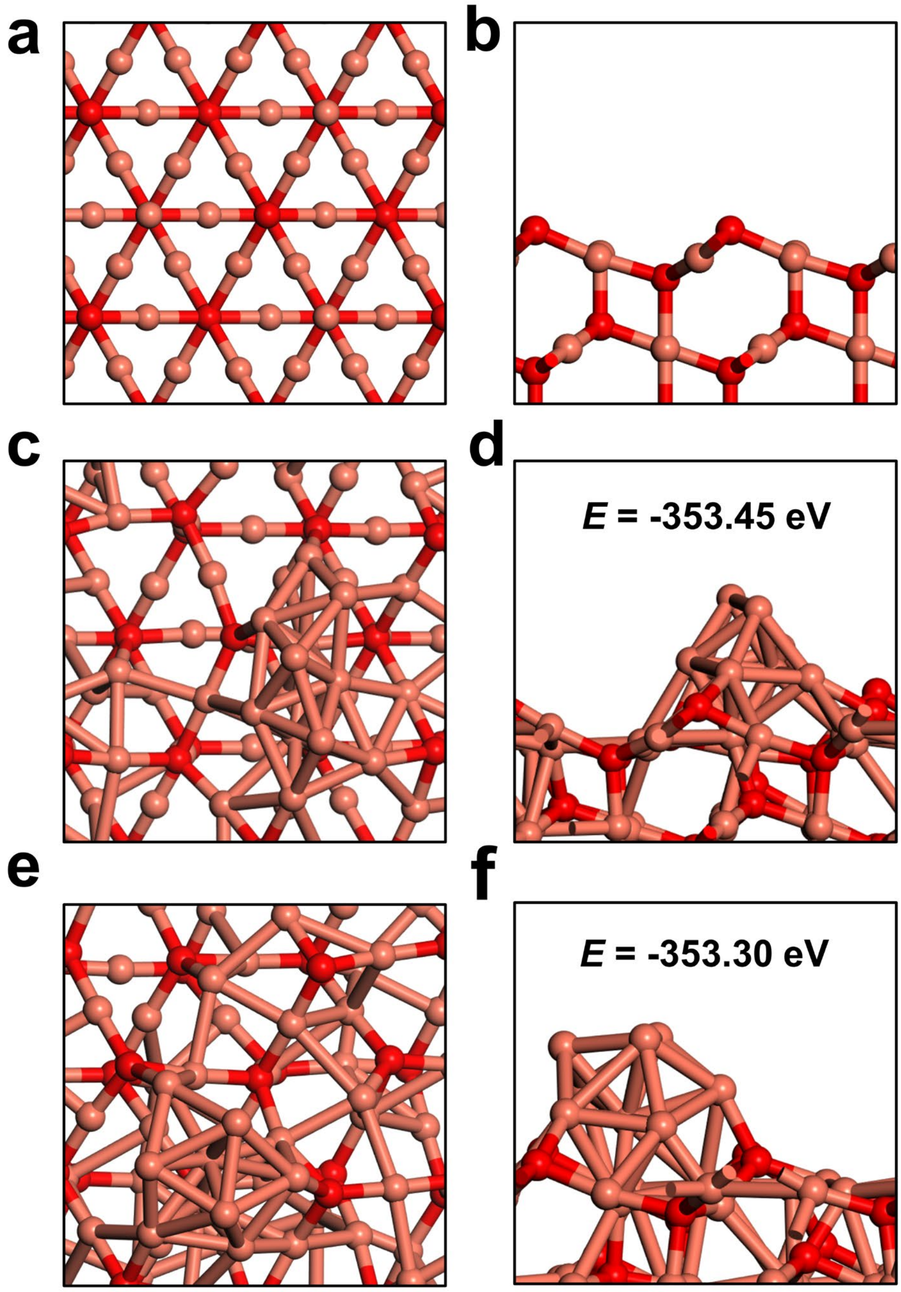

Supplementary Figure 60. DFT calculation models of Cu(111)/Cu_2O(111). a,**b** The model of optimized Cu_2O(111) crystal face. The Cu cluster with 8 atoms located **c**,**d** vertically and **e**,**f** horizontally on the surface of Cu_2O (111), respectively (red and orange balls represent O and Cu atoms, respectively; *E* is the structural stability energy).

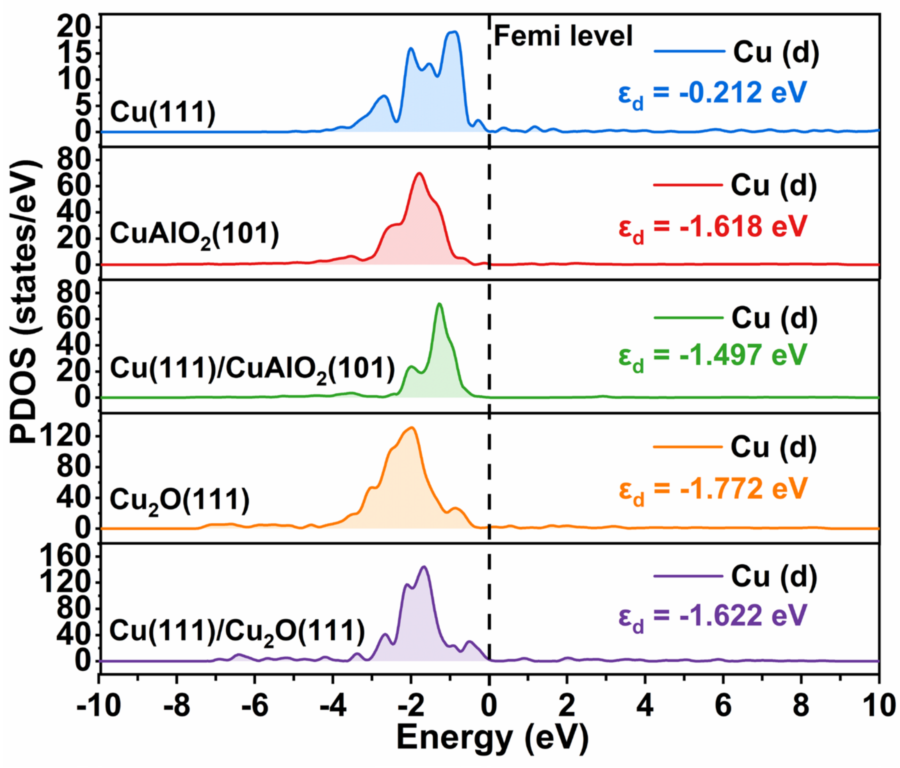

Supplementary Figure 61. PDOS analysis of various models. Projected density of states (PDOS) for Cu(111), $CuAlO_2$(101), Cu(111)/$CuAlO_2$(101), Cu_2O(111) and Cu(111)/Cu_2O(111) models.

Supplementary Note 5

Three kinds of supercells (1 × 4 × 1) for $CuAlO_2$(101) with different outermost atoms were built based on the $CuAlO_2$(101) unit cell (Supplementary Fig. 58). Compared with the O-exposed ($CuAlO_2$(101)-O, Supplementary Fig. 58c,d) and Al-exposed ($CuAlO_2$(101)-Al, Supplementary Figs. 58e,f) surface, Cu-exposed surface shows the highest stability energy ($CuAlO_2$(101)-Cu, Supplementary Figs. 58g,h). Then, the Cu_8 cluster deriving from the crystal facet of Cu(111) was installed on the surface of $CuAlO_2$(101) in a vertical and horizontal manner (Supplementary Fig. 59), respectively, to build the Cu(111)/$CuAlO_2$(101) model. The vertical orientation (Supplementary Figs. 59c,e) gives a higher stability and therefore is chosen in the following calculations. Similarly, for the Cu(111)/Cu_2O(111), the Cu_8 cluster laying on the optimized Cu_2O(111) surface with vertically orientation is also more stable than the horizontal manner (Supplementary Fig. 60). According to the

calculated projected density of states (PDOS) results (Supplementary Fig. 61), the Cu(111)/$CuAlO_2$(101) (ε_d = −1.479 eV) and Cu(111)/Cu_2O (ε_d = −1.622 eV) system displays a mediate *d* band center, in comparison with the Cu(111) (ε_d = −0.212 eV), $CuAlO_2$(101) (ε_d = −1.618 eV) and Cu_2O(111) (−1.772 eV) ones, confirming a remarkable electron coupling at the Cu^0–Cu^+ interfacial sites.

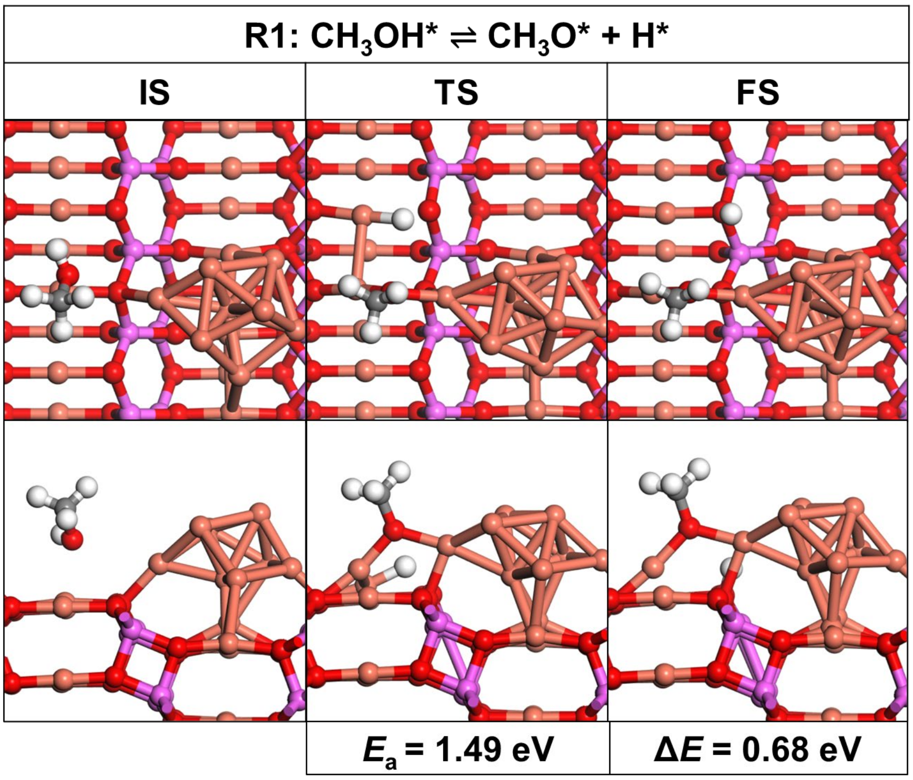

Supplementary Figure 62. DFT studies for CH_3OH dehydrogenation on Cu(111)/$CuAlO_2$(101). Calculated potential energy diagram and corresponding geometric structures for the dehydrogenation of CH_3OH on the surface of Cu(111)/$CuAlO_2$(101) (*, IS, TS and FS represent the adsorption state, initial state, transition state and final state, respectively, E_a, and ΔE is the energy barrier and thermodynamic energy).

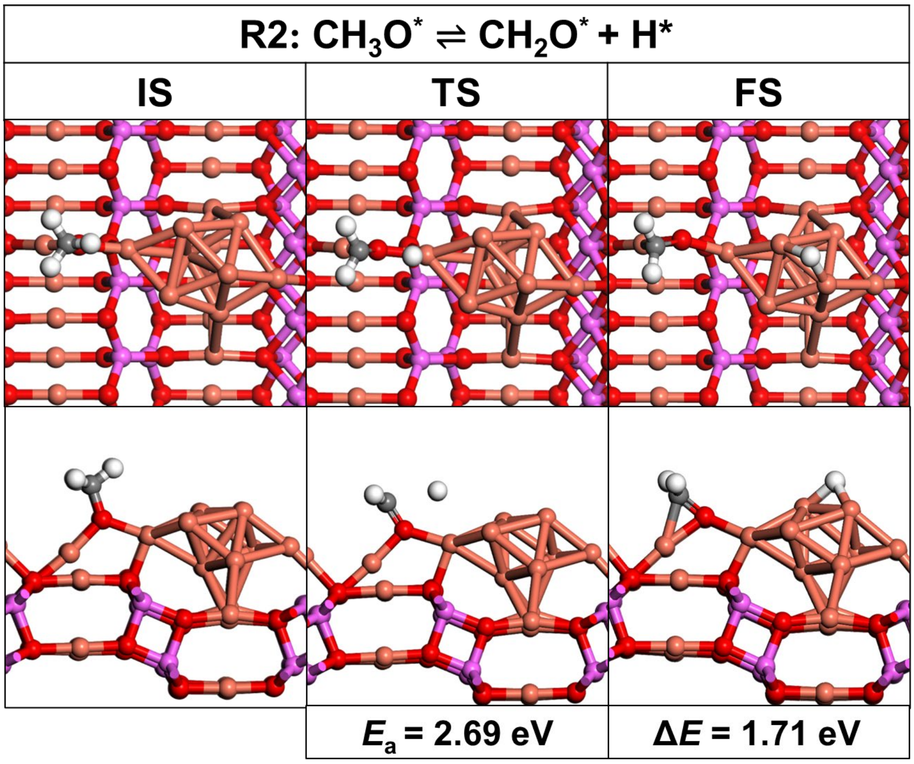

Supplementary Figure 63. DFT studies for CH_3O dehydrogenation on Cu(111)/$CuAlO_2$(101). Calculated potential energy diagram and corresponding geometric structures for the dehydrogenation of CH_3O* on the surface of Cu(111)/$CuAlO_2$(101) (*, IS, TS and FS represent the adsorption state, initial state, transition state and final state, respectively, E_a, and ΔE is the energy barrier and thermodynamic energy).

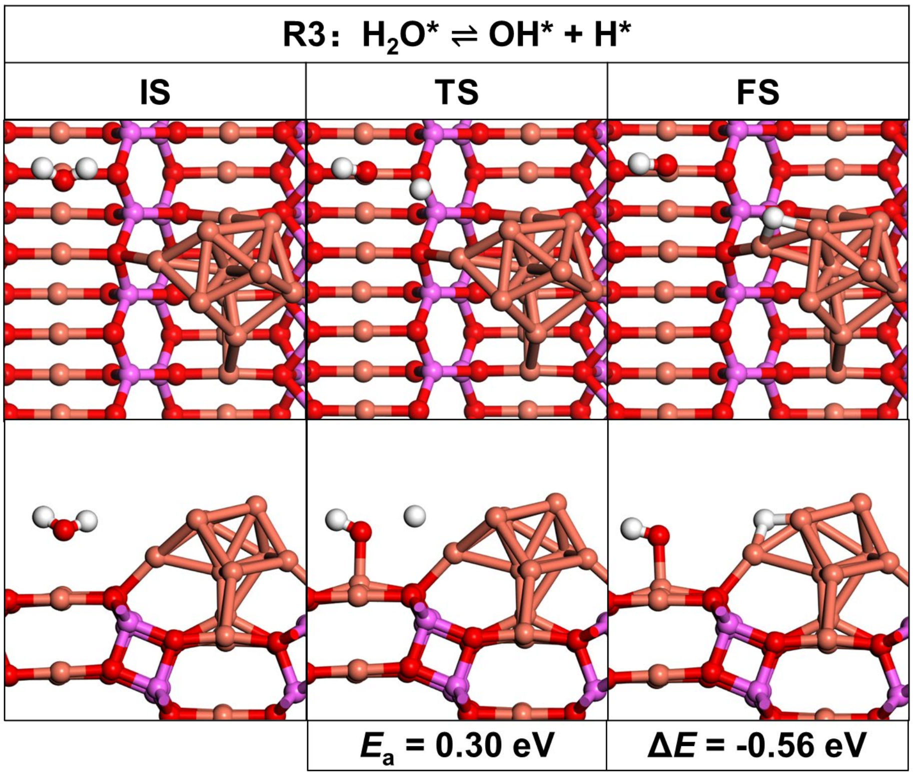

Supplementary Figure 64. DFT studies for H_2O dissociation on Cu(111)/$CuAlO_2$(101). Calculated potential energy diagram and corresponding geometric structures for the dissociation of H_2O on the surface of Cu(111)/$CuAlO_2$(101) (*, IS, TS and FS represent the adsorption state, initial state, transition state and final state, respectively, E_a, and ΔE is the energy barrier and thermodynamic energy).

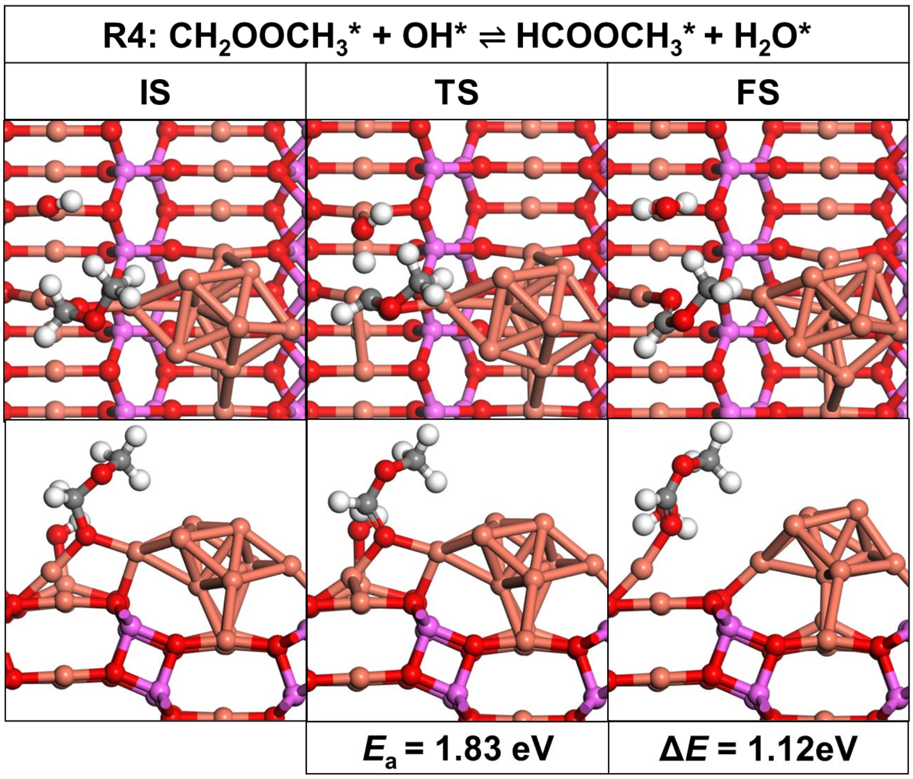

Supplementary Figure 65. DFT studies for CH_2OOCH_3 dehydrogenation on Cu(111)/$CuAlO_2$(101). Calculated potential energy diagram and corresponding geometric structures for the hydroxyl assisted dehydrogenation of CH_2OOCH_3 to $HCOOCH_3$ on the surface of Cu(111)/$CuAlO_2$(101) (*, IS, TS and FS represent the adsorption state, initial state, transition state and final state, respectively, E_a, and ΔE is the energy barrier and thermodynamic energy).

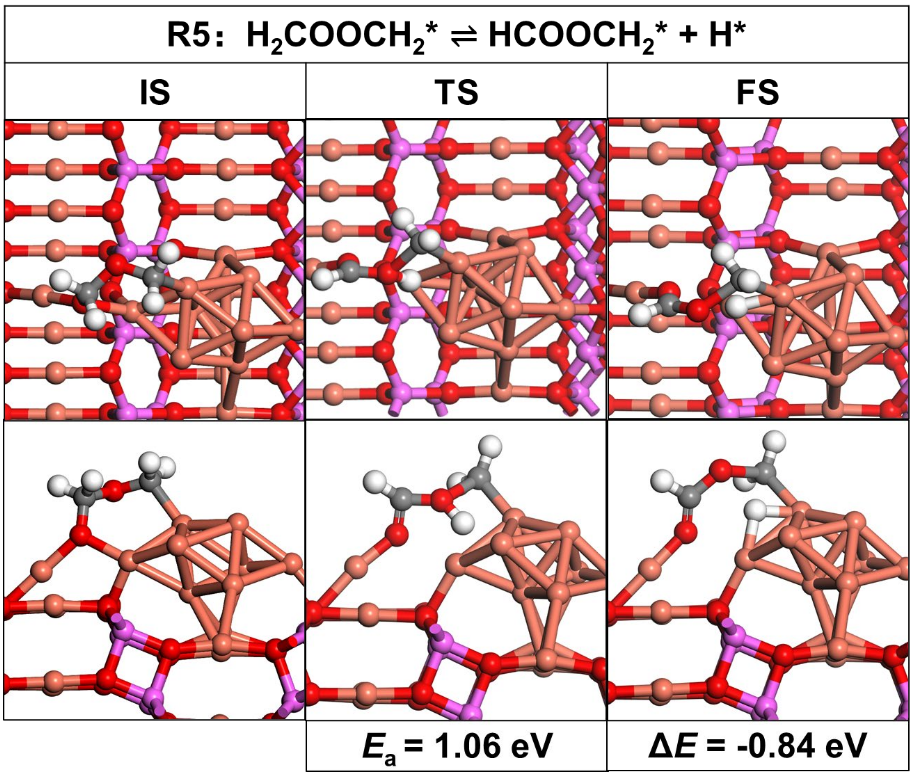

Supplementary Figure 66. DFT studies for CH_2OOCH_2 dehydrogenation on Cu(111)/$CuAlO_2$(101). Calculated potential energy diagram and corresponding geometric structures for the dehydrogenation of H_2COOCH_2 to $HCOOCH_2$ on the surface of Cu(111)/$CuAlO_2$(101) (*, IS, TS and FS represent the adsorption state, initial state, transition state and final state, respectively. E_a and ΔE are the energy barrier and thermodynamic energy).

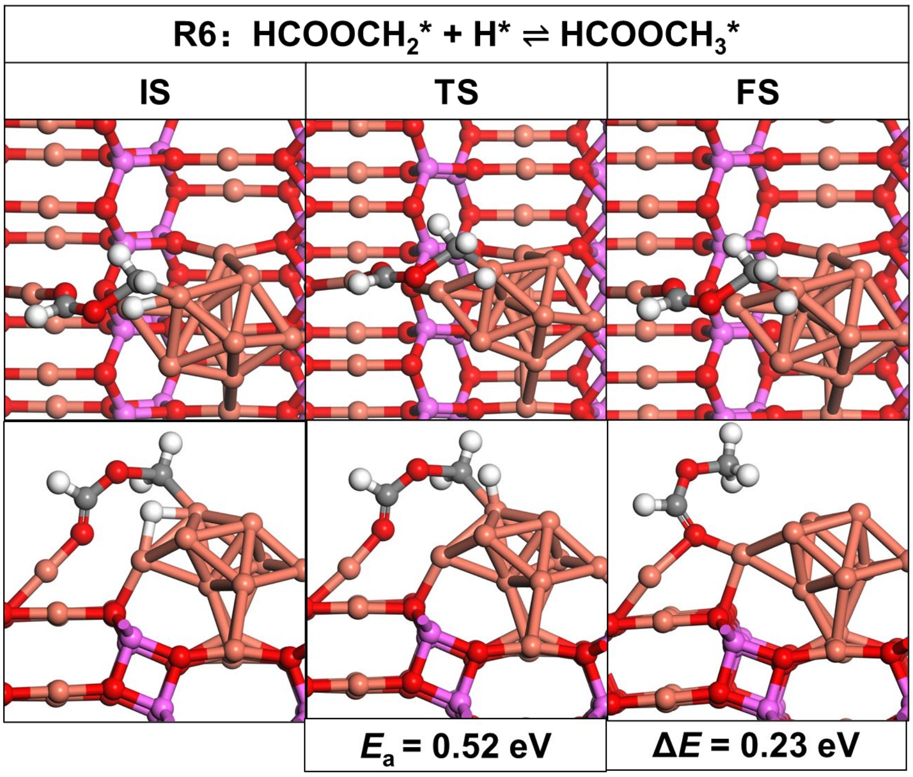

Supplementary Figure 67. DFT studies for $HCOOCH_2$ hydrogenation on Cu(111)/$CuAlO_2$(101). Calculated potential energy diagram and corresponding geometric structures for the hydrogenation of $HCOOCH_2$ to $HCOOCH_3$ on the surface of Cu(111)/$CuAlO_2$(101) (*, IS, TS and FS represent the adsorption state, initial state, transition state and final state, respectively. E_a and ΔE are the energy barrier and thermodynamic energy).

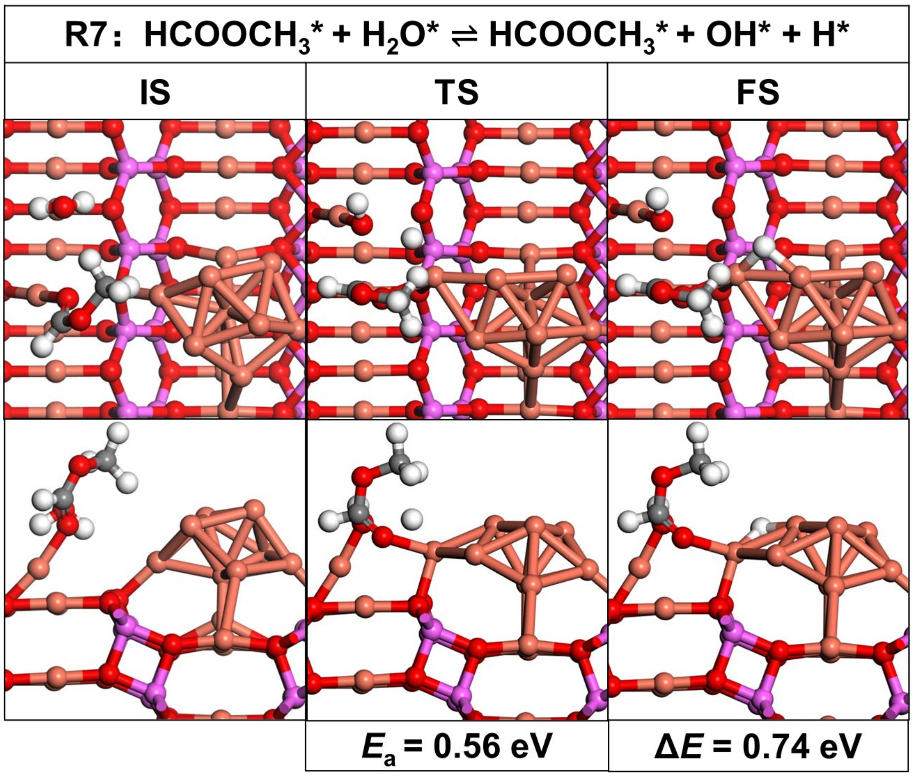

Supplementary Figure 68. DFT studies for H_2O dissociation on Cu(111)/$CuAlO_2$(101). Calculated potential energy diagram and corresponding geometric structures for the dissociation of H_2O on the surface of Cu(111)/$CuAlO_2$(101) with the existence of $HCOOCH_3$ (*, IS, TS and FS represent the adsorption state, initial state, transition state and final state, respectively, E_a, and ΔE is the energy barrier and thermodynamic energy).

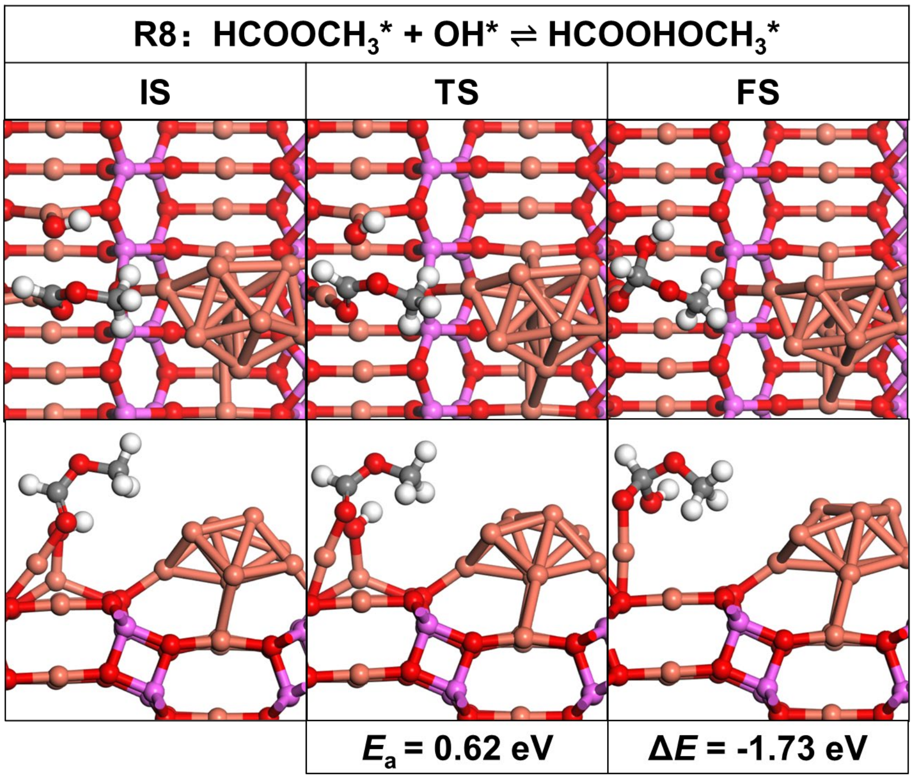

Supplementary Figure 69. DFT studies for $HCOOCH_3$ hydrolysis on Cu(111)/$CuAlO_2$(101). Calculated potential energy diagram and corresponding geometric structures for the hydrolysis of $HCOOCH_3$ on the surface of Cu(111)/$CuAlO_2$(101) (*, IS, TS and FS represent the adsorption state, initial state, transition state and final state, respectively, E_a, and ΔE is the energy barrier and thermodynamic energy).

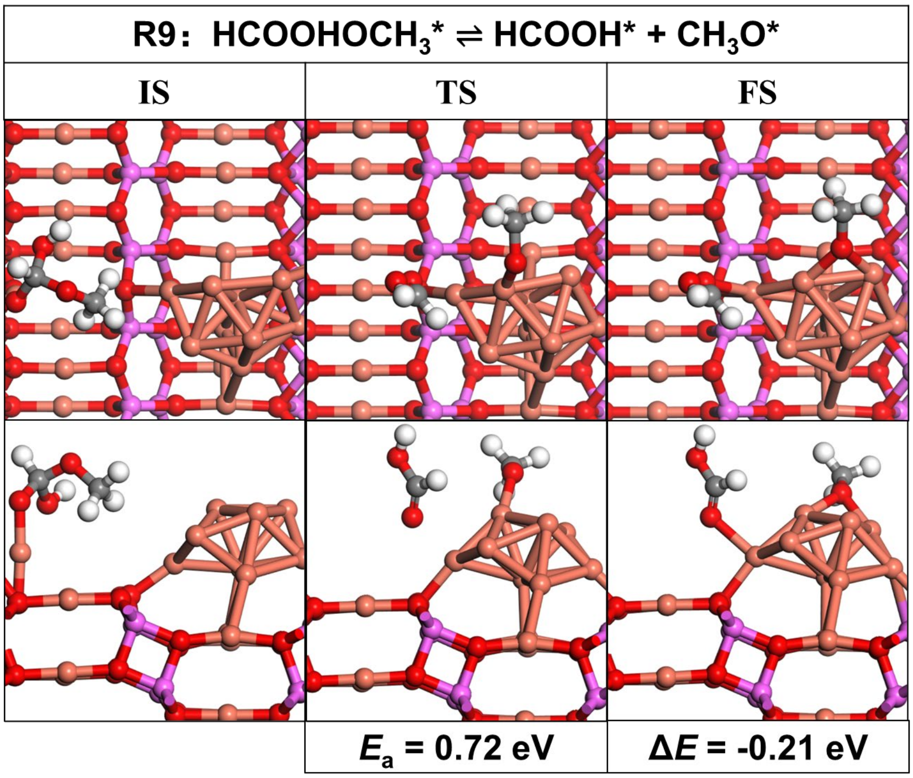

Supplementary Figure 70. DFT studies for $HCOOHOCH_3$ hydrolysis on Cu(111)/$CuAlO_2$(101). Calculated potential energy diagram and corresponding geometric structures for the dissociation of $HCOOHOCH_3$ to HCOOH and CH_3O* on the surface of Cu(111)/$CuAlO_2$(101) (*, IS, TS and FS represent the adsorption state, initial state, transition state and final state, respectively, E_a, and ΔE is the energy barrier and thermodynamic energy).

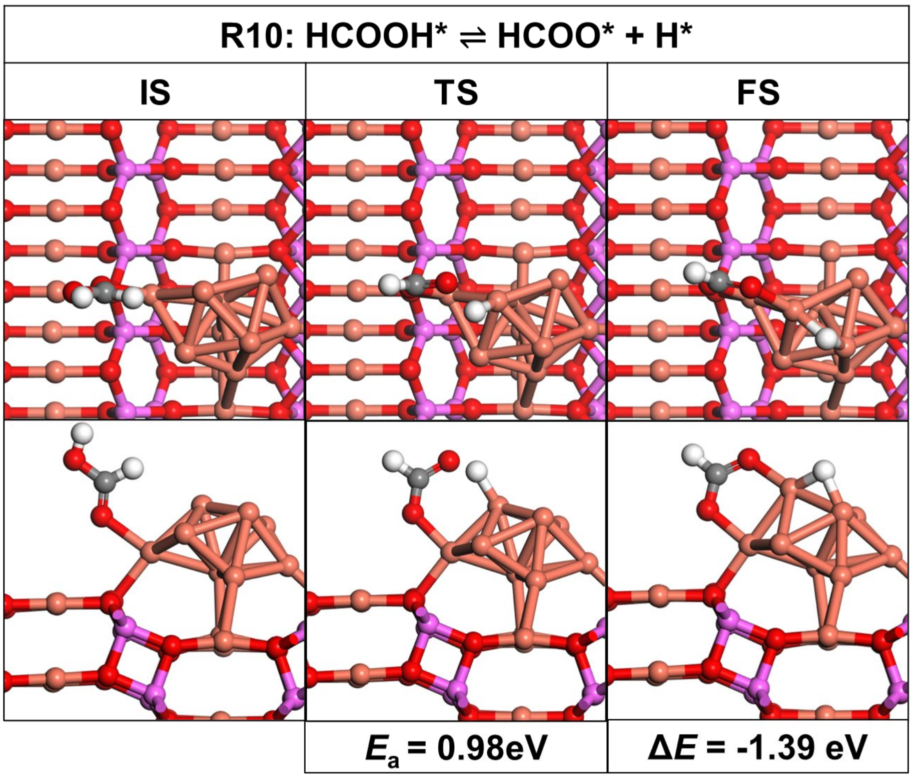

Supplementary Figure 71. DFT studies for HCOOH dehydrogenation on Cu(111)/$CuAlO_2$(101). Calculated potential energy diagram and corresponding geometric structures for the dehydrogenation of HCOOH on the surface of Cu(111)/$CuAlO_2$(101) (*, IS, TS and FS represent the adsorption state, initial state, transition state and final state, respectively, E_a, and ΔE is the energy barrier and thermodynamic energy).

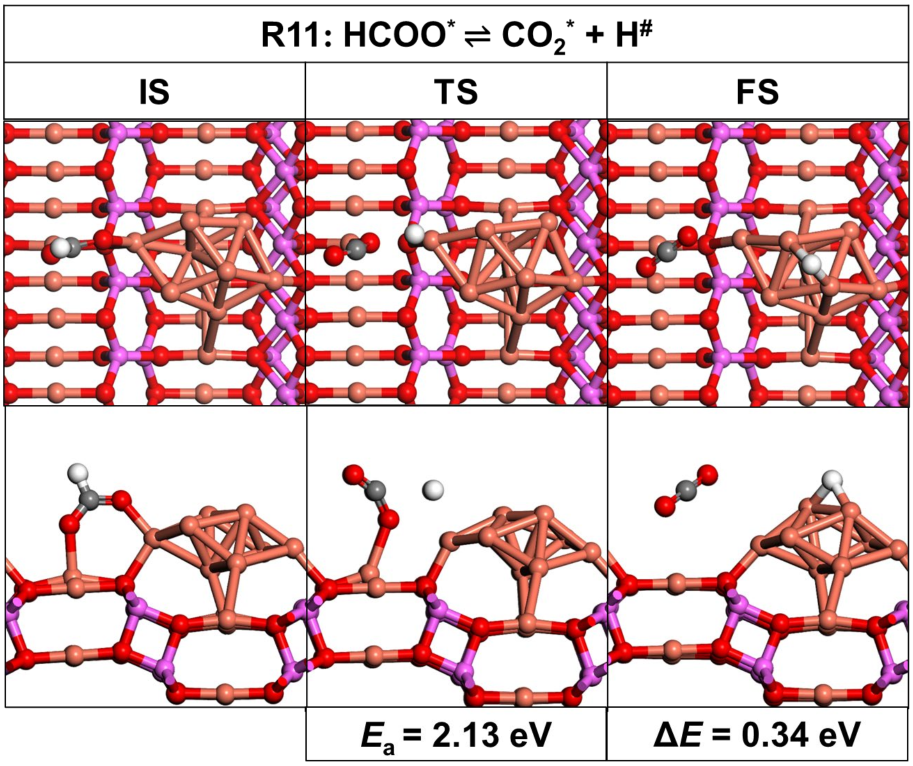

Supplementary Figure 72. DFT studies for HCOO dehydrogenation on Cu(111)/$CuAlO_2$(101). Calculated potential energy diagram and corresponding geometric structures for the dehydrogenation of HCOO* on the surface of Cu(111)/$CuAlO_2$(101) (*, IS, TS and FS represent the adsorption state, initial state, transition state and final state, respectively, E_a, and ΔE is the energy barrier and thermodynamic energy).

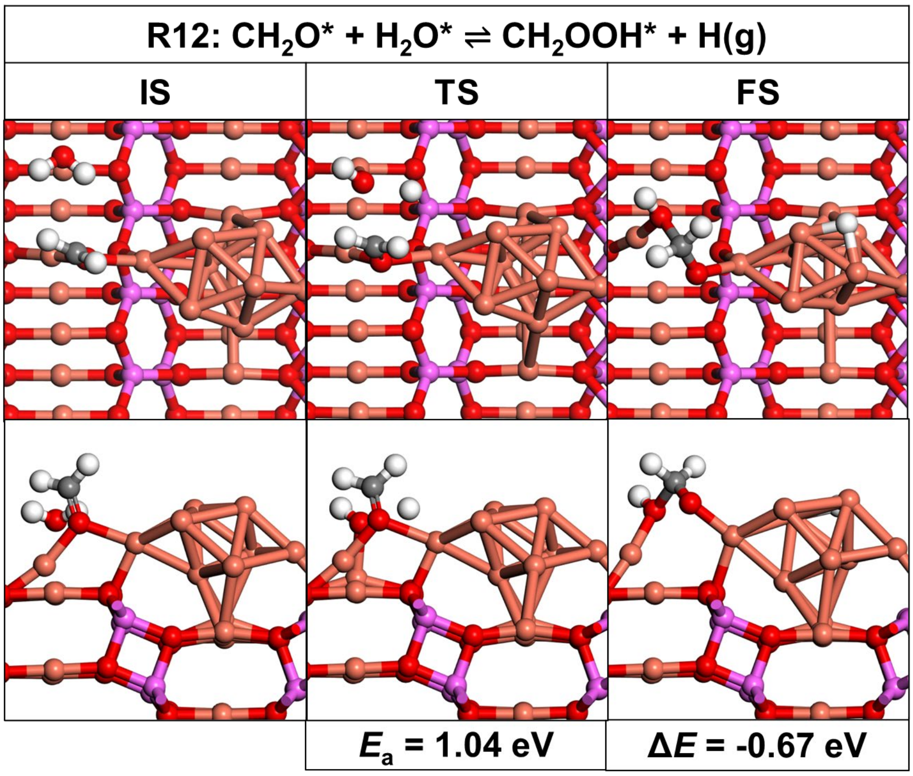

Supplementary Figure 73. DFT studies for CH_2O oxidation on Cu(111)/$CuAlO_2$(101). Calculated potential energy diagram and corresponding geometric configurations for the oxidation of CH_2O on the surface of Cu(111)/$CuAlO_2$(101) (*, IS, TS and FS represent the adsorption state, initial state, transition state and final state, respectively; E_a and ΔE is the energy barrier and thermodynamic energy).

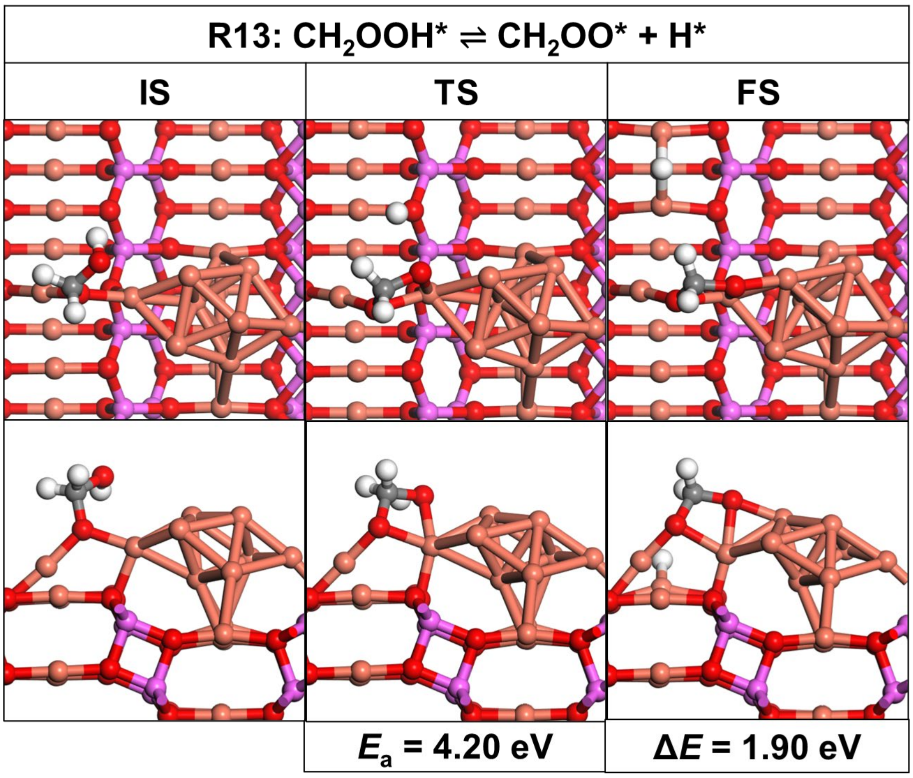

Supplementary Figure 74. DFT studies for CH_2OOH dehydrogenation on Cu(111)/CuAlO_2(101). Calculated potential energy diagram and corresponding geometric configurations for the dehydrogenation of CH_2OOH on the surface of Cu(111)/CuAlO_2(101) (*, IS, TS and FS represent the adsorption state, initial state, transition state and final state, respectively; E_a and ΔE is the energy barrier and thermodynamic energy).

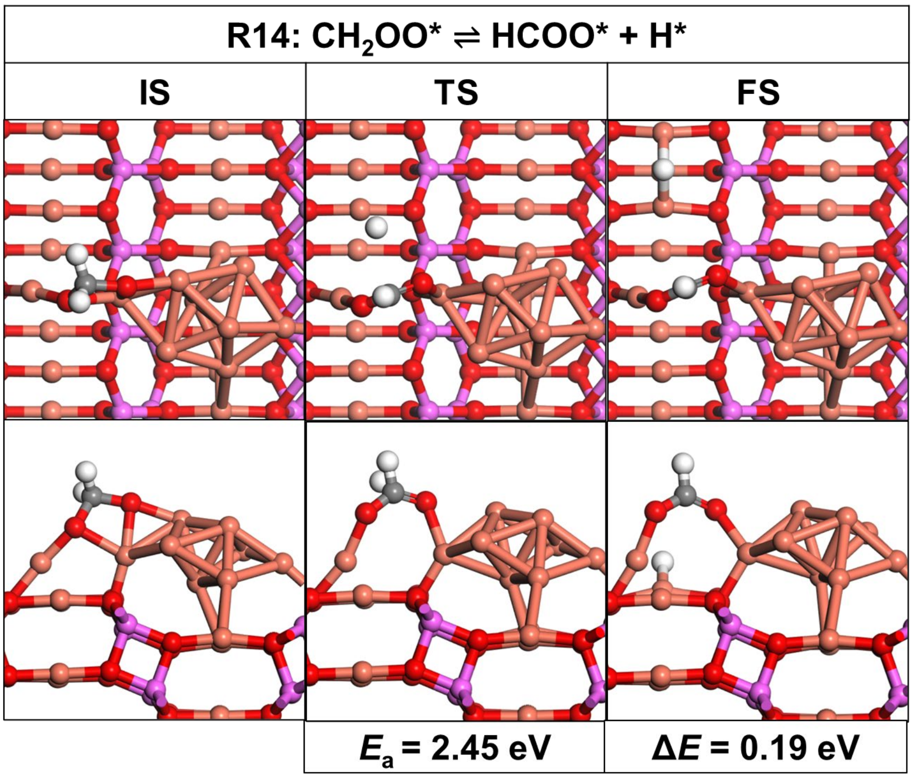

Supplementary Figure 75. DFT studies for CH_2OO dehydrogenation on Cu(111)/$CuAlO_2$(101). Calculated potential energy diagram and corresponding geometric configurations for the dehydrogenation of CH_2OO* on the surface of Cu(111)/$CuAlO_2$(101) (*, IS, TS and FS represent the adsorption state, initial state, transition state and final state, respectively; E_a and ΔE is the energy barrier and thermodynamic energy).

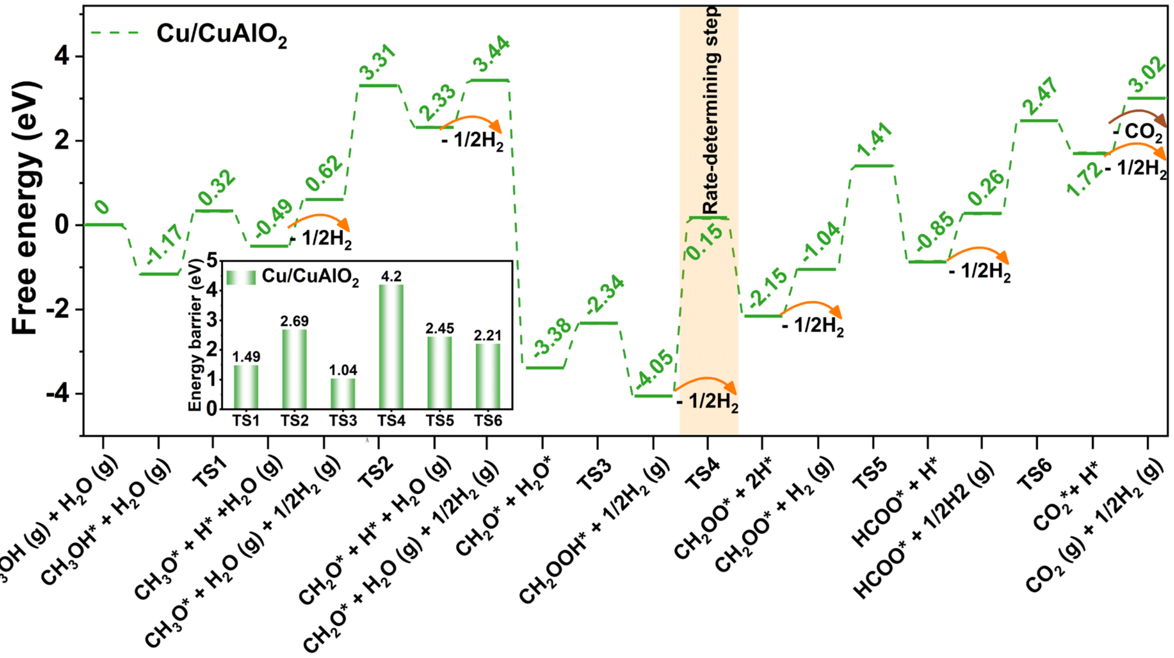

Supplementary Figure 76. Energy curve from DFT calculation on Cu(111)/$CuAlO_2$(101). Full-path analysis for MSR reaction following the formaldehyde oxidation mechanism over Cu(111)/$CuAlO_2$(101). 'TS' denotes the transition state. Numbers located at the horizontal line represent the free energy of corresponding intermediates (the inset gives the energy barrier of each transient state).

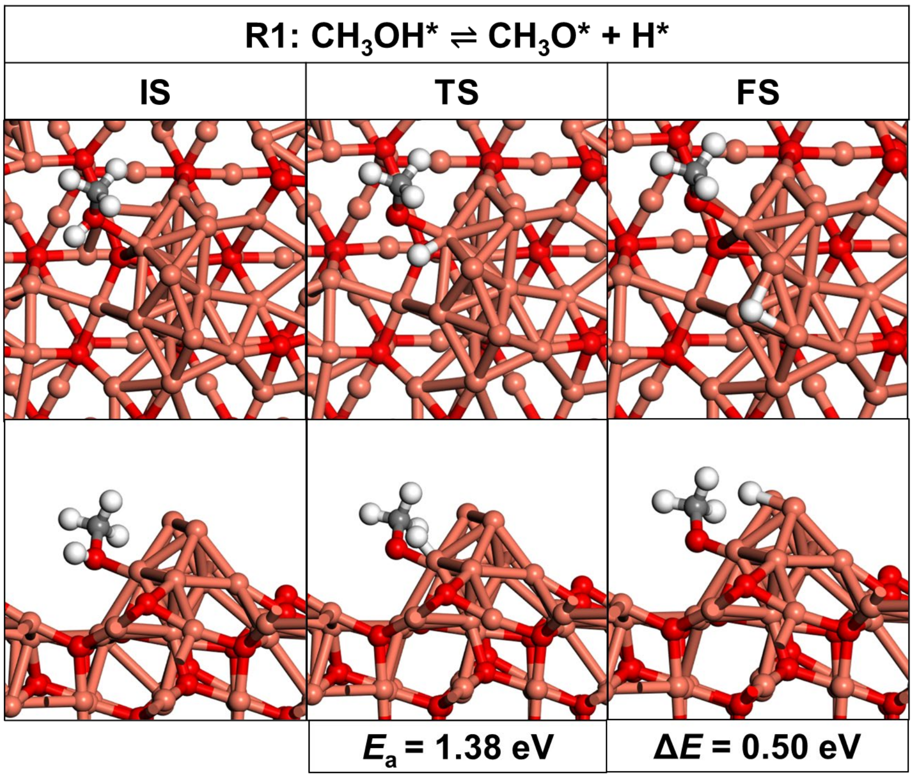

Supplementary Figure 77. DFT studies for CH_3OH dehydrogenation on Cu(111)/Cu_2O(111). Calculated potential energy diagram and corresponding geometric configurations for the dehydrogenation of CH_3OH on the surface of Cu(111)/Cu_2O(111) (*, IS, TS and FS represent the adsorption state, initial state, transition state and final state, respectively; E_a and ΔE is the energy barrier and thermodynamic energy).

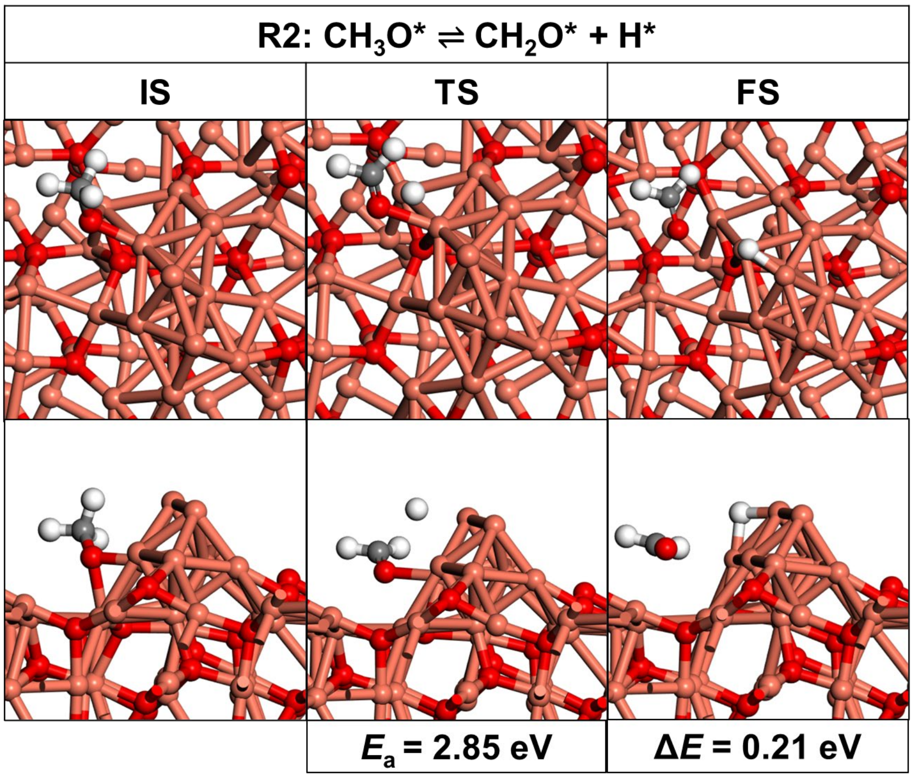

Supplementary Figure 78. DFT studies for CH_3O dehydrogenation on Cu(111)/Cu_2O(111). Calculated potential energy diagram and corresponding geometric configurations for the dehydrogenation of CH_3O* on the surface of Cu(111)/Cu_2O(111) (*, IS, TS and FS represent the adsorption state, initial state, transition state and final state, respectively; E_a and ΔE is the energy barrier and thermodynamic energy).

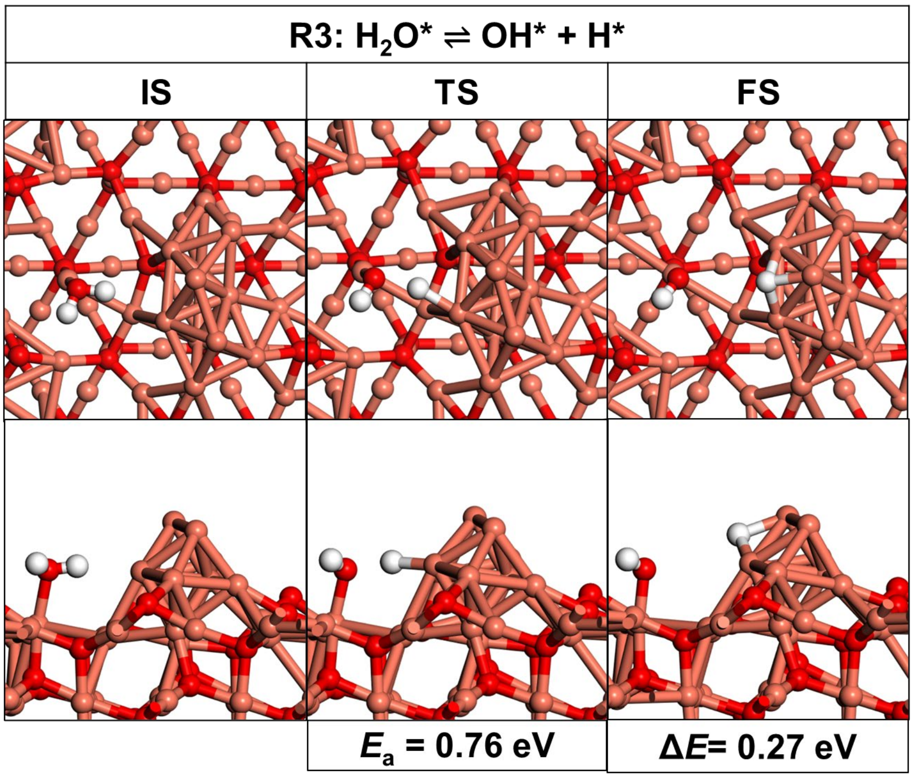

Supplementary Figure 79. DFT studies for H_2O dissociation on Cu(111)/Cu_2O(111). Calculated potential energy diagram and corresponding geometric configurations for the dissociation of H_2O on the surface of Cu(111)/Cu_2O(111) (*, IS, TS and FS represent the adsorption state, initial state, transition state and final state, respectively; E_a and ΔE is the energy barrier and thermodynamic energy).

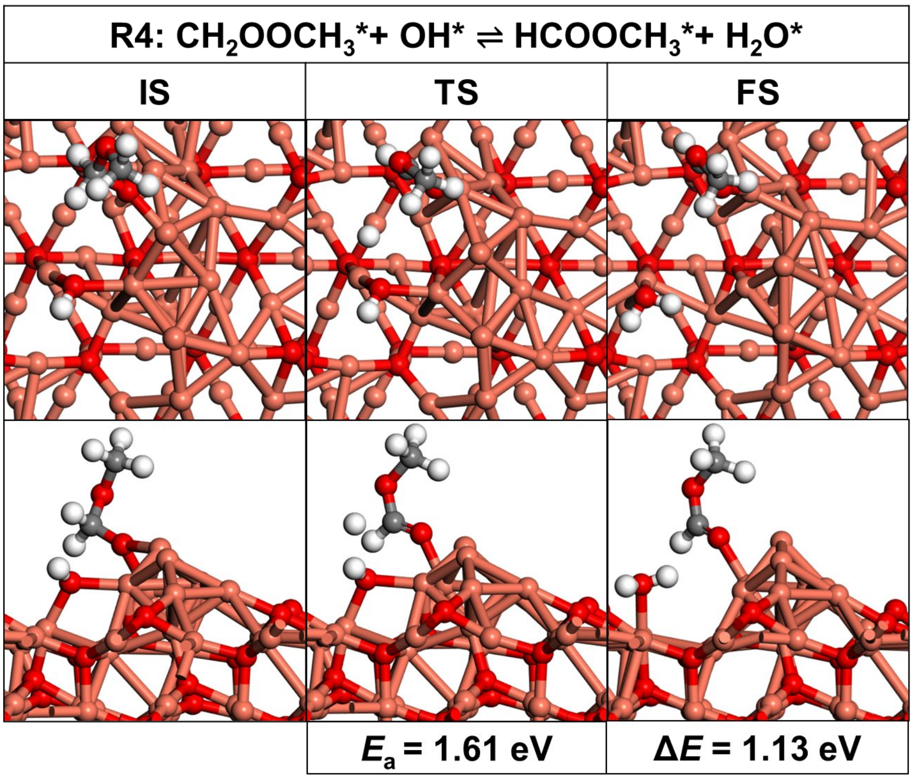

Supplementary Figure 80. DFT studies for CH_2OOCH_3 dehydrogenation on Cu(111)/Cu_2O(111). Calculated potential energy diagram and corresponding geometric configurations for the hydroxyl assisted dehydrogenation of CH_2OOCH_3 on the surface of Cu(111)/Cu_2O(111) (*, IS, TS and FS represent the adsorption state, initial state, transition state and final state, respectively; E_a and ΔE is the energy barrier and thermodynamic energy).

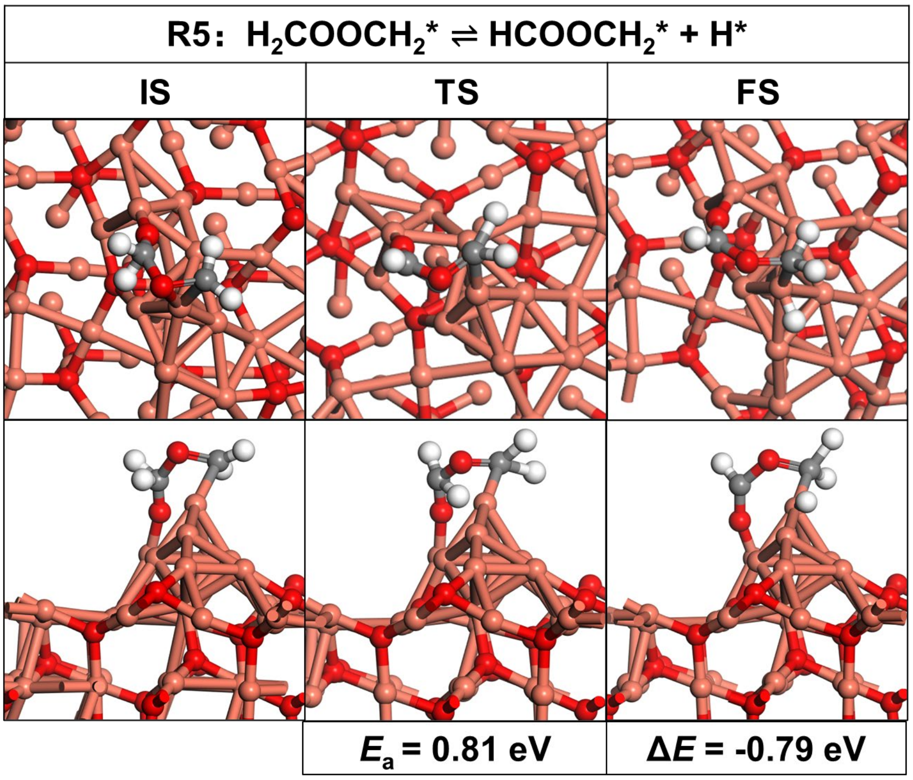

Supplementary Figure 81. DFT studies for CH_2OOCH_2 dehydrogenation on Cu(111)/Cu_2O(111). Calculated potential energy diagram and corresponding geometric structures for the dehydrogenation of H_2COOCH_2 to $HCOOCH_2$ on the surface of Cu(111)/Cu_2O(111) (*, IS, TS and FS represent the adsorption state, initial state, transition state and final state, respectively. E_a and ΔE are the energy barrier and thermodynamic energy).

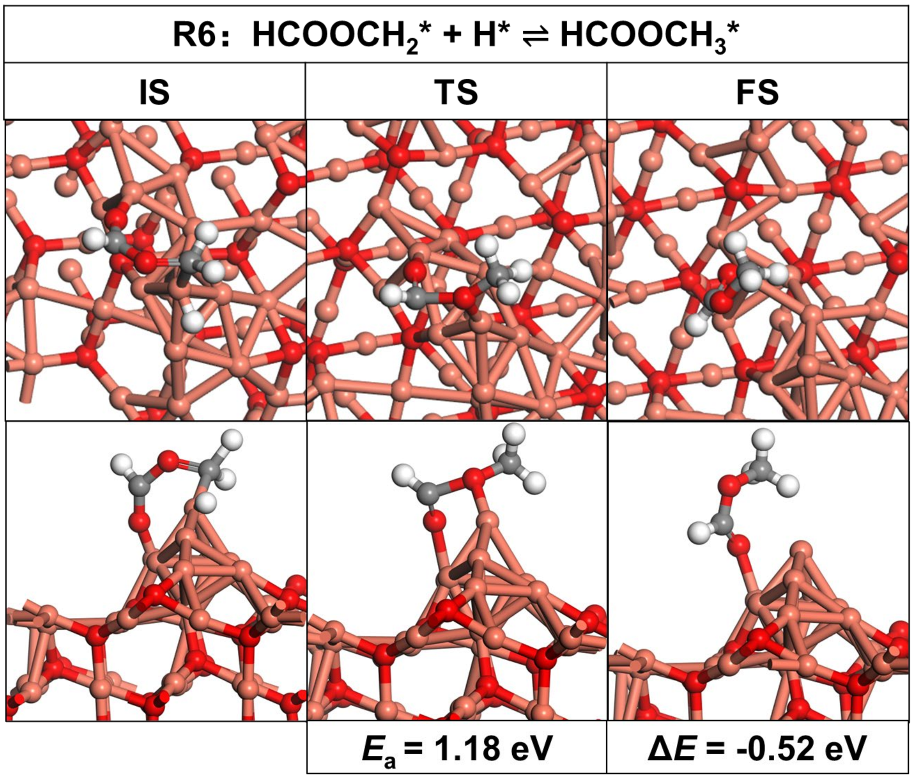

Supplementary Figure 82. DFT studies for CH_2OOCH_3 dehydrogenation on Cu(111)/Cu_2O(111). Calculated potential energy diagram and corresponding geometric structures for the hydrogenation of $HCOOCH_2$ to $HCOOCH_3$ on the surface of Cu(111)/Cu_2O(111) (*, IS, TS and FS represent the adsorption state, initial state, transition state and final state, respectively. E_a and ΔE are the energy barrier and thermodynamic energy).

Supplementary Note 6

According to the calculation result, the formaldehyde dimerization ($2CH_2O^* \rightarrow CH_2OOCH_2^*$) is a spontaneous barrier free process on both Cu/$CuAlO_2$ and Cu/Cu_2O models. For the formation of methyl formate intermediate, compared with the hydroxyl assisted dehydrogenation route (1.83 eV on Cu/$CuAlO_2$ and 1.61 eV on Cu/Cu_2O: $CH_2OOCH_3^* + OH^* \rightarrow HCOOCH_3^* + H_2O^*$) (Supplementary Figs. 65,80), the conversion of $CH_2OOCH_2^*$ to methyl formate through direct dehydrogenation process (1.06 eV on Cu/$CuAlO_2$ and 1.18 eV on Cu/Cu_2O: $CH_2OOCH_2^* \rightarrow HCOOCH_3^*$) (Supplementary Figs. 66,67,81,82) shows a lower reaction energy barrier, indicating

that the conversion of formaldehyde to methyl formate is the favorable path for methyl formate formation ($2CH_2O^* \rightarrow HCOOCH_3^*$). Subsequently, the introduction of water promotes the hydrolysis of methyl formate (0.72 eV on $Cu/CuAlO_2$ and 1.15 eV on Cu/Cu_2O: $HCOOCH_3^* + OH^* \rightarrow HCOOH^* + CH_3O^*$) (Supplementary Figs. 69,70,84,85). The calculation results are in good agreement with the experimental data in Fig. 5d.

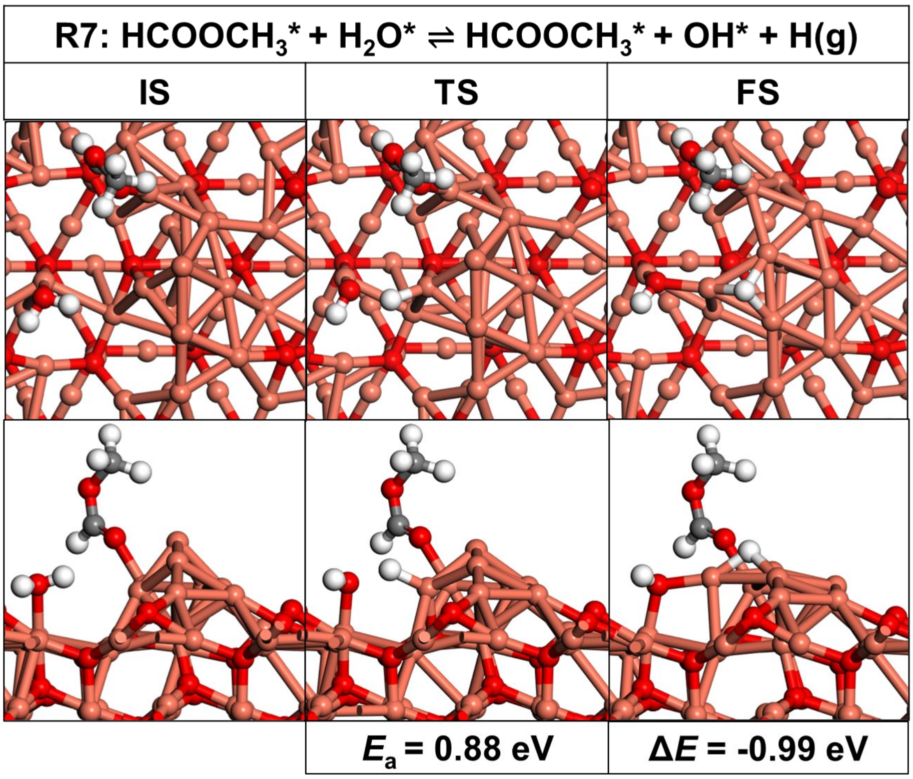

Supplementary Figure 83. DFT studies for H_2O dissociation on Cu(111)/Cu_2O(111). Calculated potential energy diagram and corresponding geometric configurations for the dissociation of H_2O on the surface of Cu(111)/Cu_2O(111) with the existence of $HCOOCH_3$ (*, IS, TS and FS represent the adsorption state, initial state, transition state and final state, respectively; E_a and ΔE is the energy barrier and thermodynamic energy).

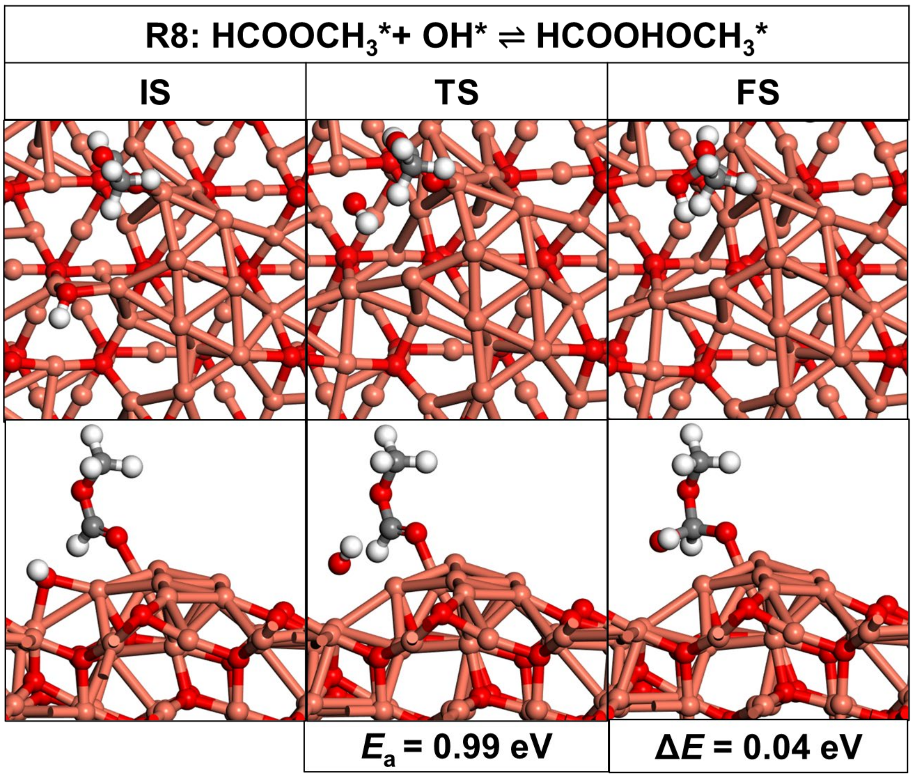

Supplementary Figure 84. DFT studies for $HCOOCH_3$ hydrolysis on Cu(111)/Cu_2O(111). Calculated potential energy diagram and corresponding geometric configurations for the hydrolysis of $HCOOCH_3$ on the surface of Cu(111)/Cu_2O(111) (*, IS, TS and FS represent the adsorption state, initial state, transition state and final state, respectively; E_a and ΔE is the energy barrier and thermodynamic energy).

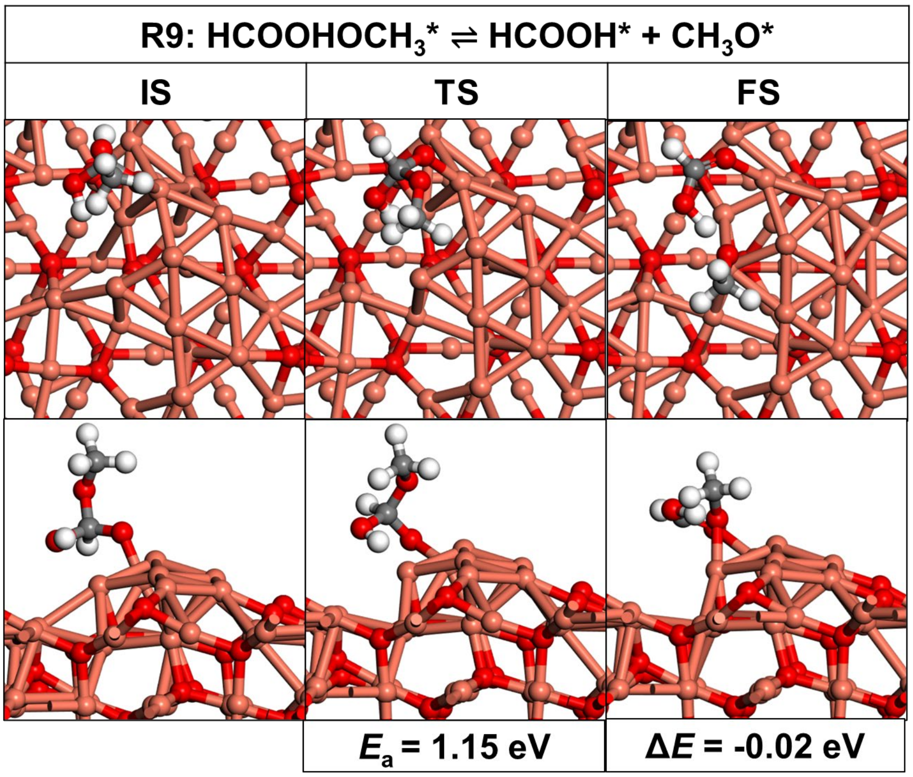

Supplementary Figure 85. DFT studies for $HCOOHOCH_3$ hydrolysis on Cu(111)/Cu_2O(111). Calculated potential energy diagram and corresponding geometric configurations for the dissociation of $HCOOHOCH_3$ to HCOOH and CH_3O* on the surface of Cu(111)/Cu_2O(111) (*, IS, TS and FS represent the adsorption state, initial state, transition state and final state, respectively; E_a and ΔE is the energy barrier and thermodynamic energy).

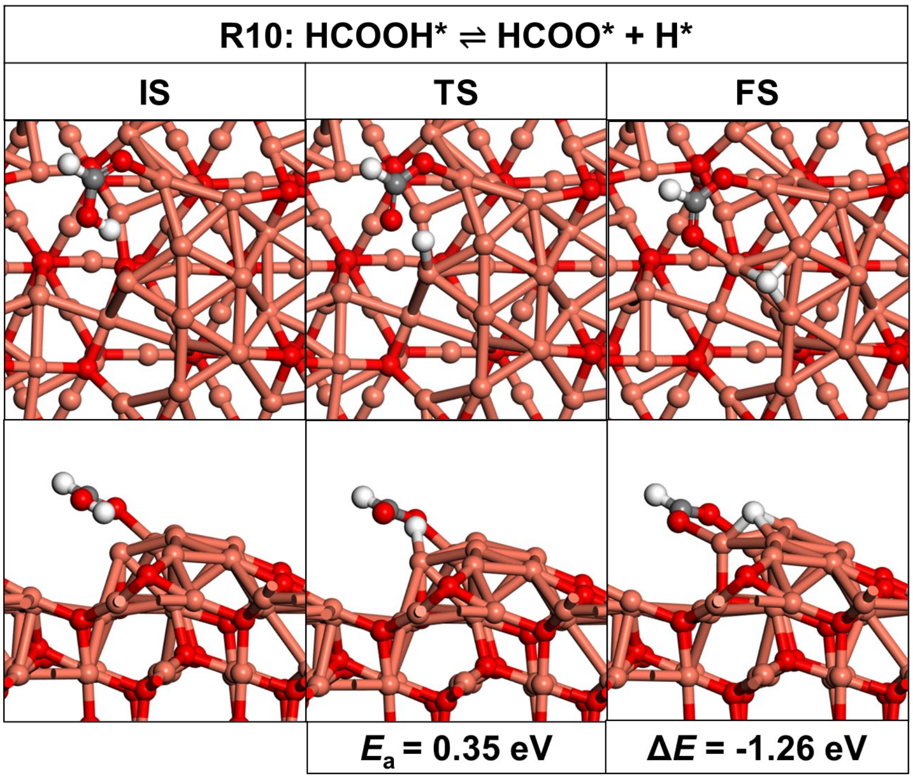

Supplementary Figure 86. DFT studies for HCOOH dehydrogenation on Cu(111)/Cu_2O(111). Calculated potential energy diagram and corresponding geometric configurations for the dehydrogenation of HCOOH on the surface of Cu(111)/Cu_2O(111) (*, IS, TS and FS represent the adsorption state, initial state, transition state and final state, respectively; E_a and ΔE is the energy barrier and thermodynamic energy).

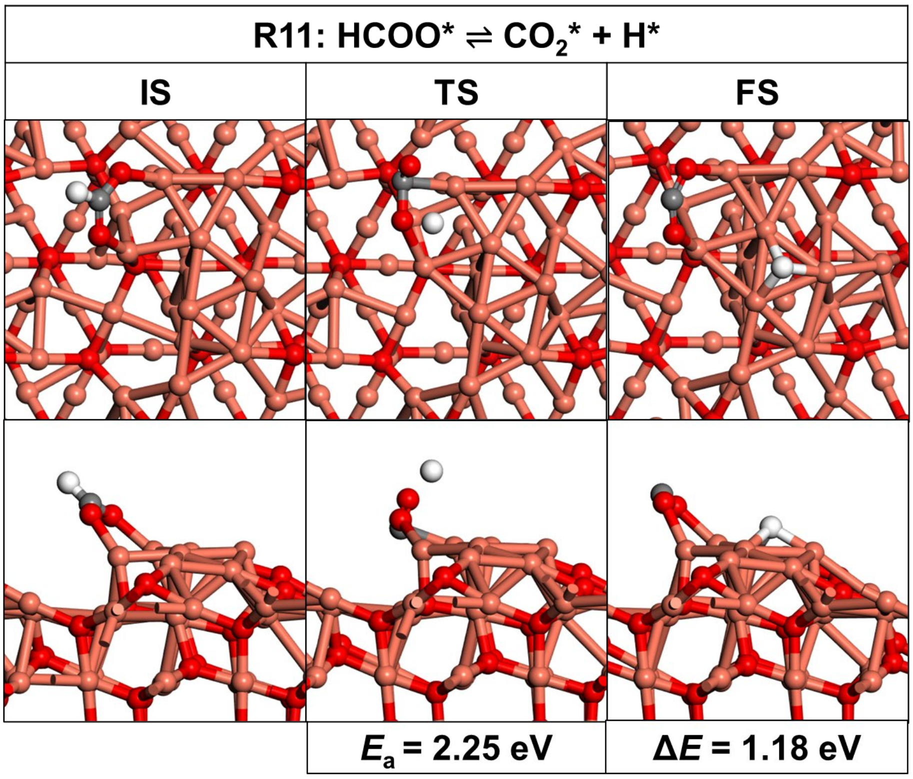

Supplementary Figure 87. DFT studies for HCOO dehydrogenation on Cu(111)/Cu_2O(111). Calculated potential energy diagram and corresponding geometric configurations for the dehydrogenation of HCOO* on the surface of Cu(111)/Cu_2O(111) (*, IS, TS and FS represent the adsorption state, initial state, transition state and final state, respectively; E_a and ΔE is the energy barrier and thermodynamic energy).

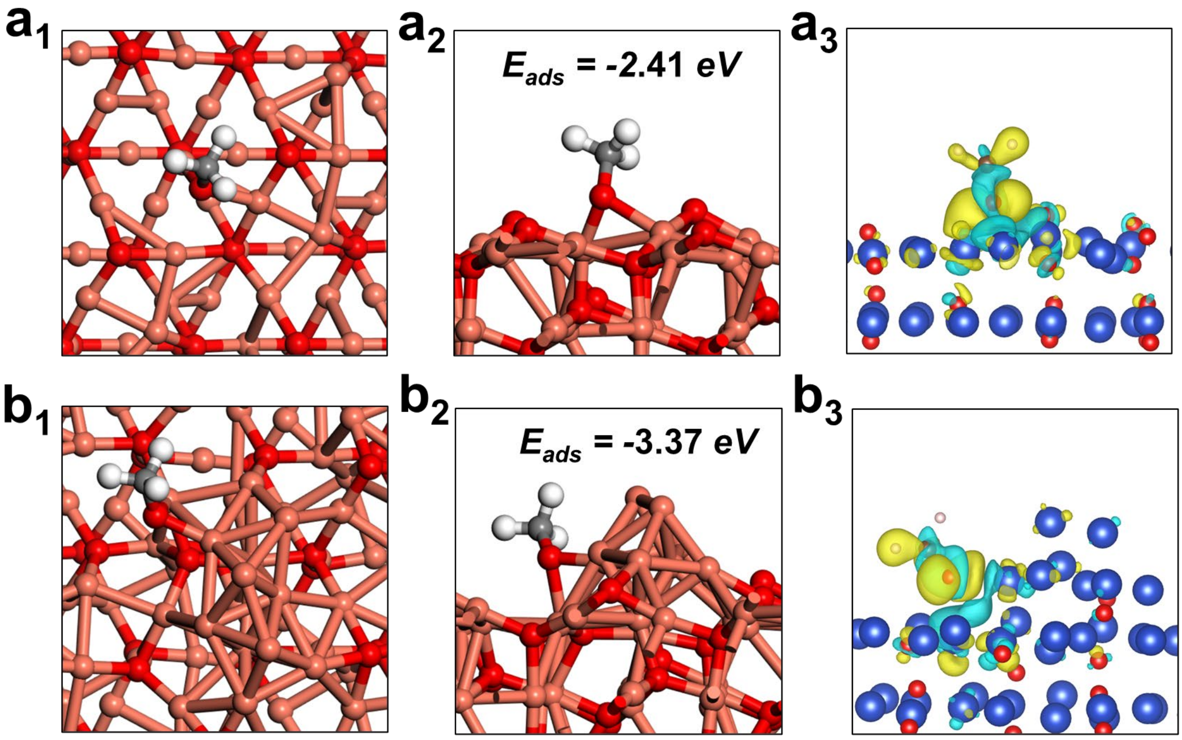

Supplementary Figure 88. DFT studies for CH_3O adsorption on $Cu_2O(111)$ and $Cu(111)/Cu_2O(111)$. Adsorption configuration of CH_3O^* species on the surface of **a_1,a_2** $Cu_2O(111)$ and **b_1,b_2** $Cu(111)/Cu_2O(111)$ (white, gray, red and orange balls represent H, C, O and Cu atoms, respectively). Corresponding charge density difference (CDD) of CH_3O^* adsorption on the surface of **a_3** $Cu_2O(111)$ and **b_3** $Cu(111)/Cu_2O(111)$.

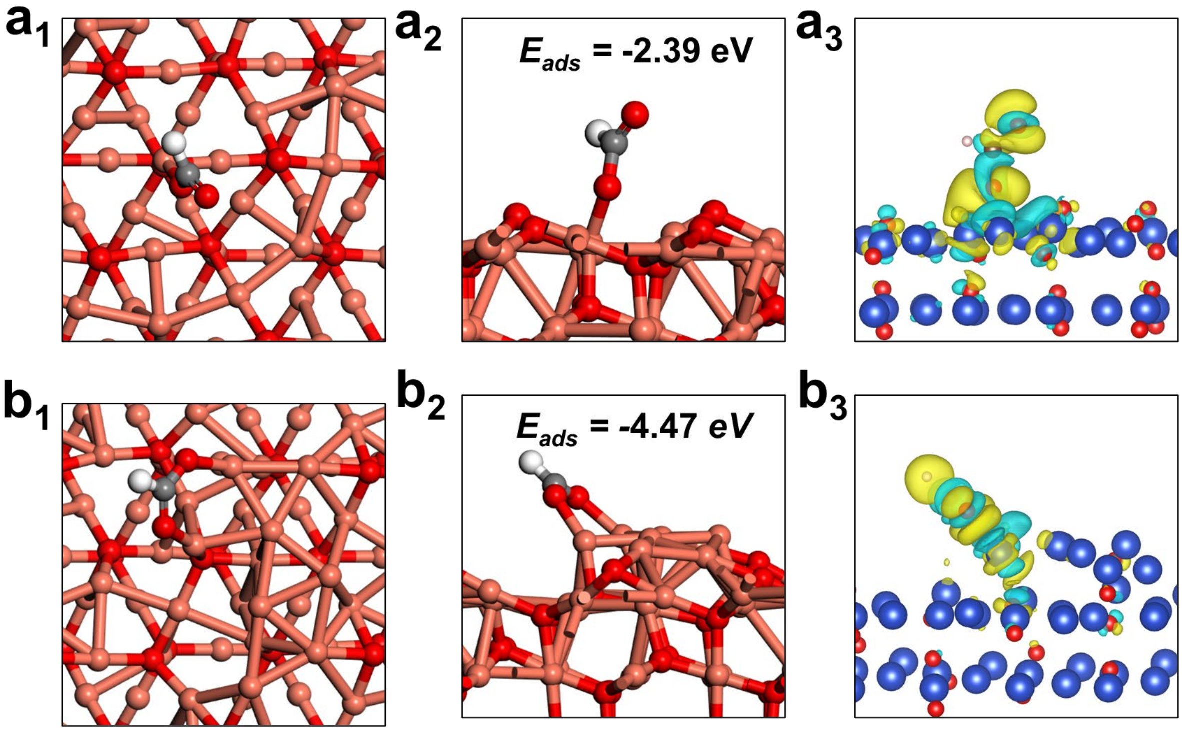

Supplementary Figure 89. DFT studies for HCOO adsorption on $Cu_2O(111)$ and $Cu(111)/Cu_2O(111)$. Adsorption configuration of HCOO* species on the surface of **a1,a2** $Cu_2O(111)$ and **b1,b2** $Cu(111)/Cu_2O(111)$ (white, gray, red and orange balls represent H, C, O and Cu atoms, respectively). Corresponding charge density difference (CDD) of HCOO* adsorption on the surface of **a3** $Cu_2O(111)$ and **b3** $Cu(111)/Cu_2O(111)$.

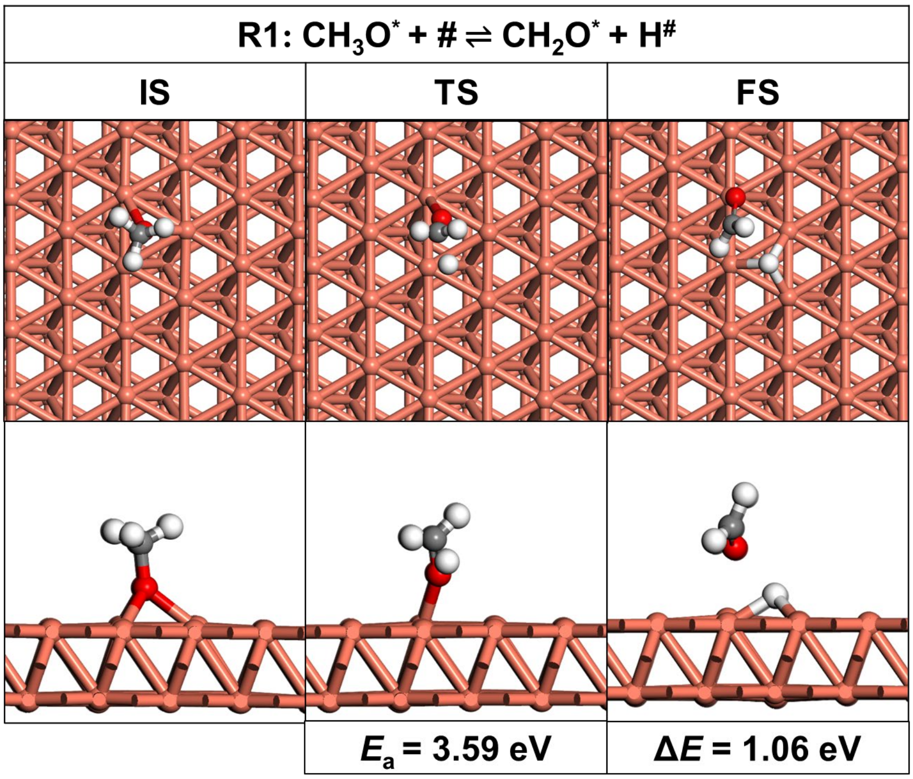

Supplementary Figure 90. DFT studies for CH_3O dehydrogenation on Cu(111). Calculated potential energy diagram and corresponding geometric configurations for the dehydrogenation of CH_3O^* on the surface of Cu(111) (*, IS, TS and FS represent the adsorption state, initial state, transition state and final state, respectively; E_a, and ΔE is the energy barrier and thermodynamic energy).

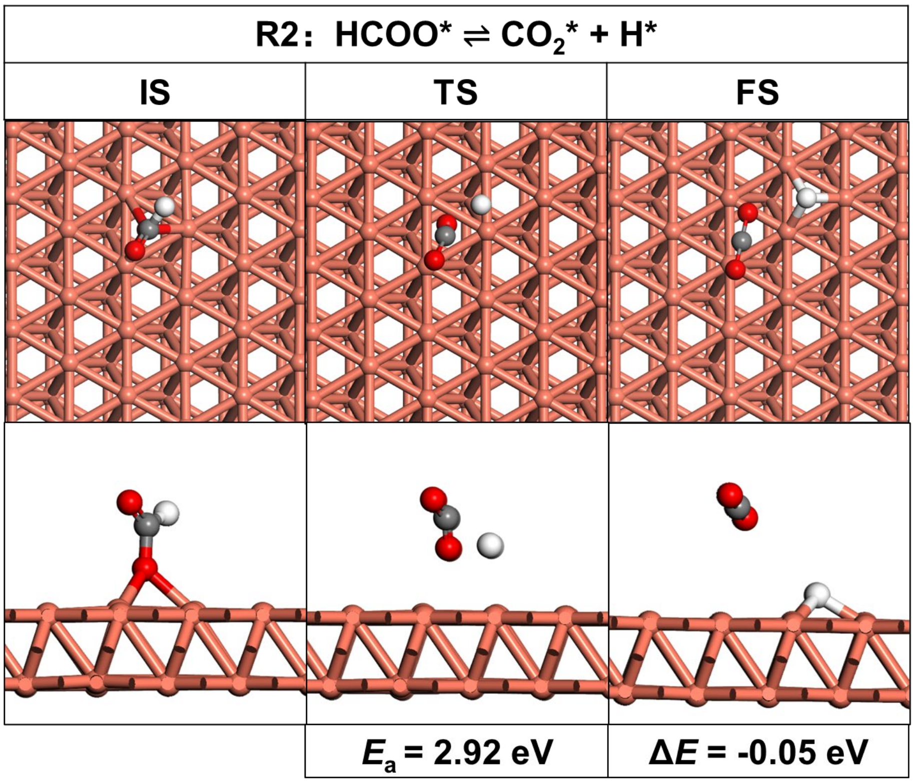

Supplementary Figure 91. DFT studies for HCOO dehydrogenation on Cu(111). Calculated potential energy diagram and corresponding geometric configurations for the dehydrogenation of HCOO* on the surface of Cu(111) (*, IS, TS and FS represent the adsorption state, initial state, transition state and final state, respectively; E_a, and ΔE is the energy barrier and thermodynamic energy).

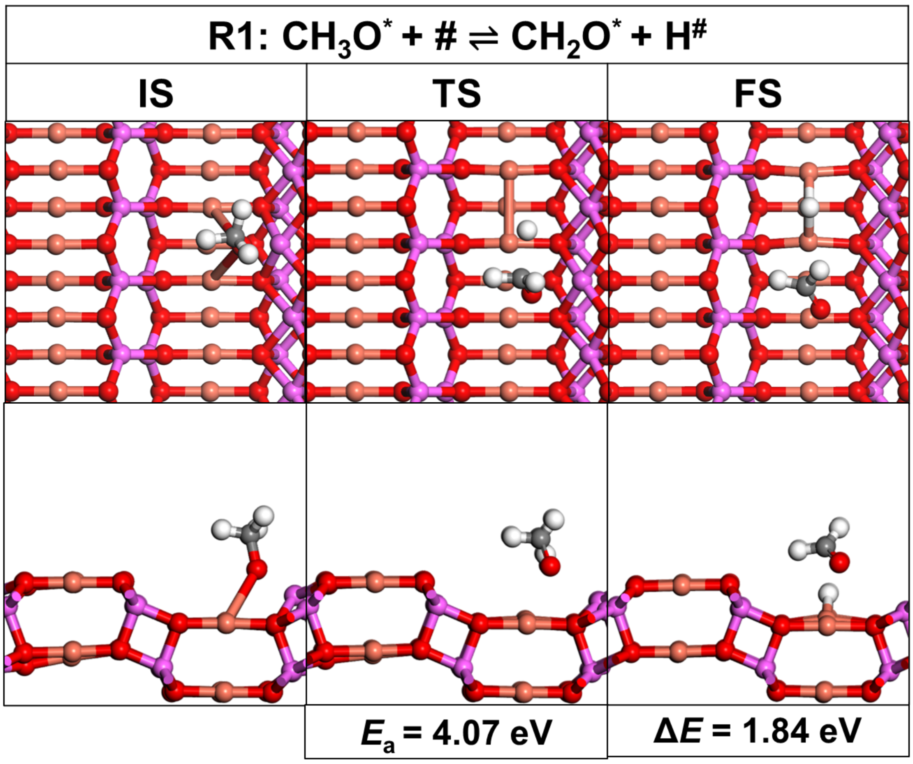

Supplementary Figure 92. DFT studies for CH_3O dehydrogenation on $CuAlO_2(101)$. Calculated potential energy diagram and corresponding geometric configurations for the dehydrogenation of CH_3O^* on the surface of $CuAlO_2(101)$ (*, IS, TS and FS represent the adsorption state, initial state, transition state and final state, respectively; E_a, and ΔE is the energy barrier and thermodynamic energy).

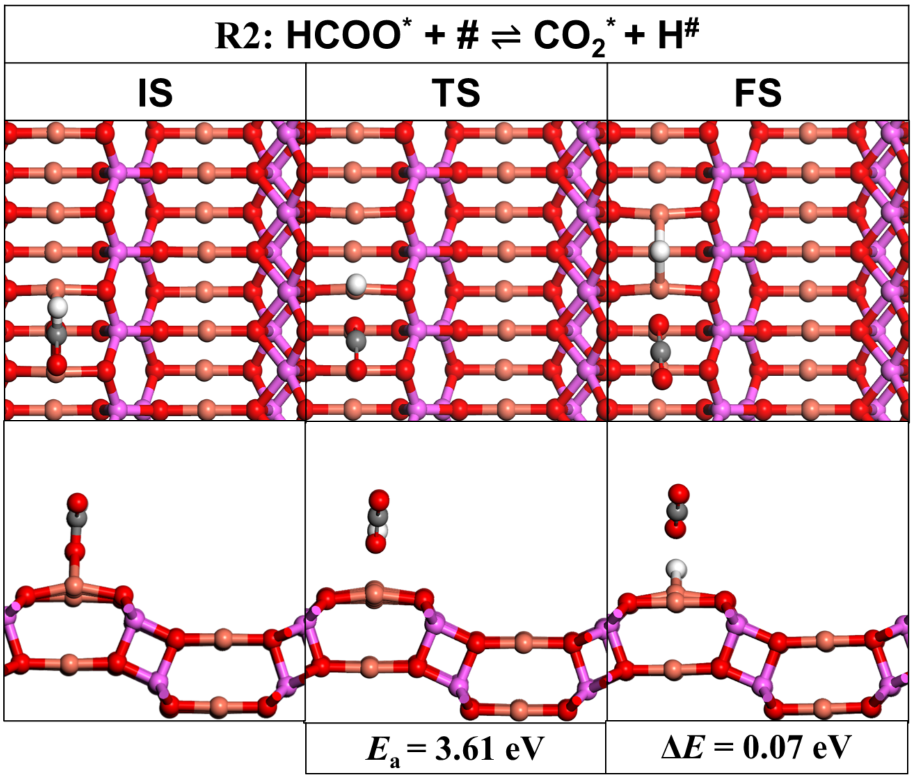

Supplementary Figure 93. DFT studies for HCOO dehydrogenation on $CuAlO_2$(101). Calculated potential energy diagram and corresponding geometric configurations for the dehydrogenation of HCOO* on the surface of $CuAlO_2$(101) (*, IS, TS and FS represent the adsorption state, initial state, transition state and final state, respectively; E_a, and ΔE is the energy barrier and thermodynamic energy).

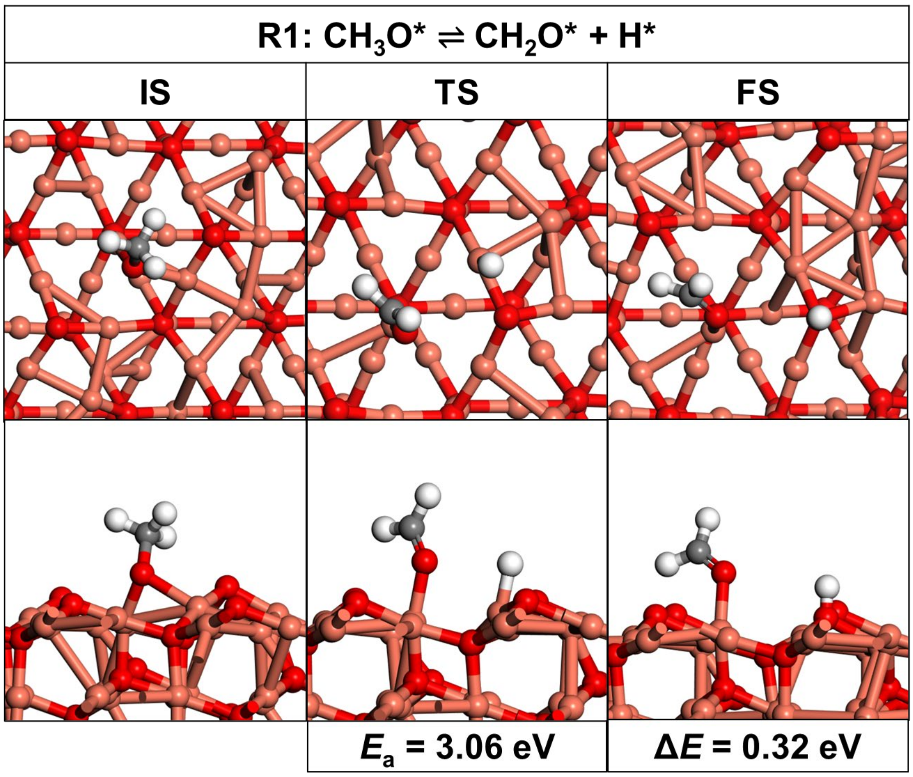

Supplementary Figure 94. DFT studies for CH_3O dehydrogenation on $Cu_2O(111)$. Calculated potential energy diagram and corresponding geometric configurations for the dehydrogenation of CH_3O^* on the surface of $Cu_2O(111)$ (*, IS, TS and FS represent the adsorption state, initial state, transition state and final state, respectively; E_a, and ΔE is the energy barrier and thermodynamic energy).

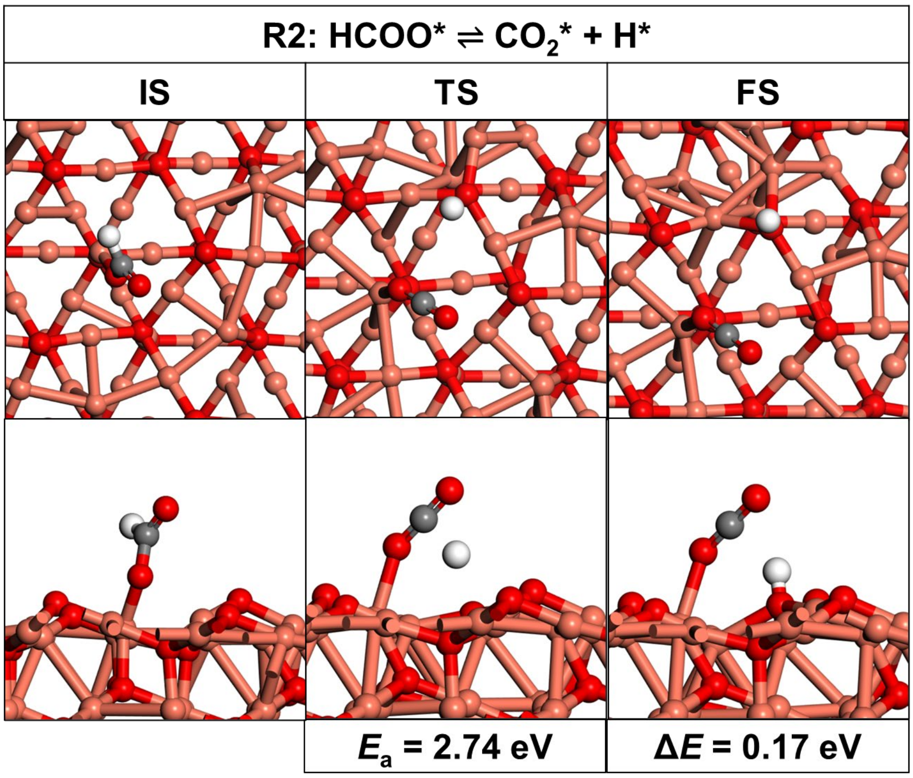

Supplementary Figure 95. DFT studies for HCOO dehydrogenation on $Cu_2O(111)$. Calculated potential energy diagram and corresponding geometric configurations for the dehydrogenation of HCOO* on the surface of $Cu_2O(111)$ (*, IS, TS and FS represent the adsorption state, initial state, transition state and final state, respectively; E_a, and ΔE is the energy barrier and thermodynamic energy).

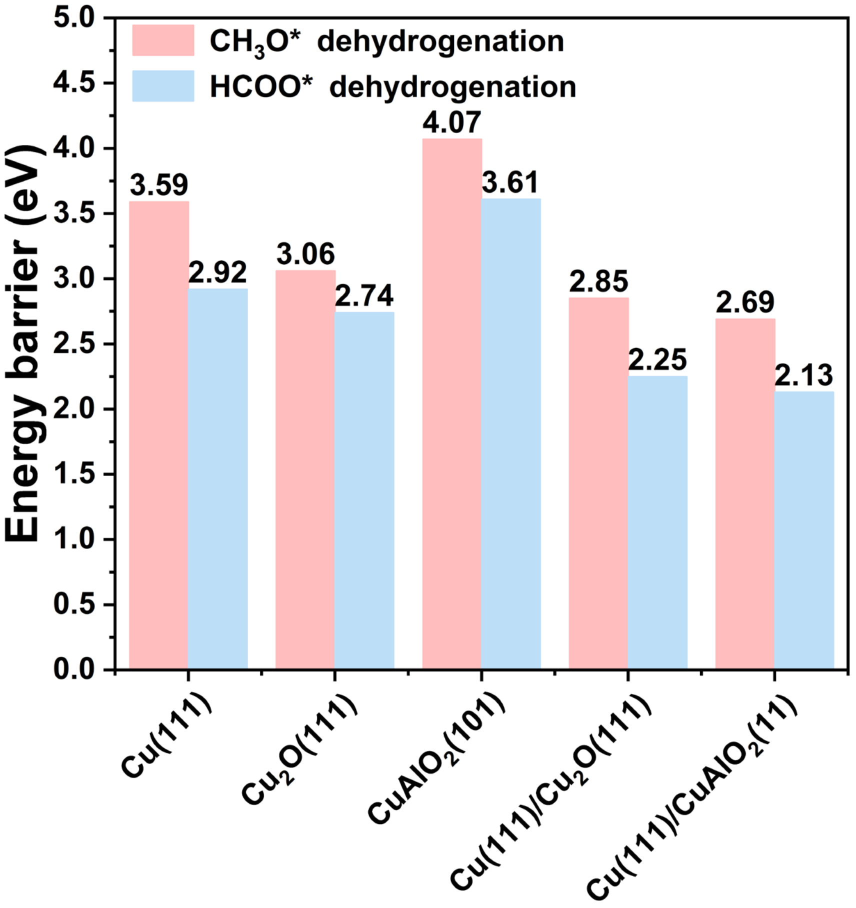

Supplementary Figure 96. Statistical result of DFT calculation. Reaction energy barrier of CH_3O* and HCOO* dehydrogenation over various models (the results from the Supplementary Figs. 63,72,78,87 and 90–95).

Supplementary Note 7

As shown in Fig. 6a, both CH_3O* and HCOO* display a tridentate adsorption configuration with relatively high adsorption energies (–5.23 and –5.12 eV) at the hollow sites of Cu(111) surface. For the $CuAlO_2$(101) system (Fig. 6b), CH_3O* and HCOO* experience bidentate adsorption at two adjacent Cu^+ sites, with relatively low adsorption energies (–2.40 and –1.54 eV). In the case of Cu(111)/$CuAlO_2$(101) system (Fig. 6c), both CH_3O* and HCOO* exhibit a unique bridge adsorption configuration at the Cu^0–Cu^+ interface sites, with adsorption energies of –2.84 and –3.09 eV, respectively, which are located between Cu(111) and $CuAlO_2$(101) ones. For the Cu(111)/Cu_2O(111),

the adsorption energy of CH_3O* and HCOO* is −2.39 and −4.47 eV, which is also located in between Cu(111) (−5.23 and −5.12 eV) and Cu_2O(111) (−2.41 and −3.37 eV). Remarkably, in comparison with Cu(111), $CuAlO_2$(101) and Cu_2O(111) systems, the energy barriers of C−H bonds cleavage for CH_3O* and HCOO* at the interface of Cu(111)/$CuAlO_2$(101) and Cu(111)/Cu_2O(111) decrease significantly (Supplementary Fig. 96), and the lowest activity energy was appeared on the Cu(111)/$CuAlO_2$(101), indicating that the Cu^0–Cu^+ bifunctional sites boost the rate-determining step of MSR reaction. The calculation results agree well with the experimental observations (kinetic studies, *in situ* FT-IR and MS analysis).

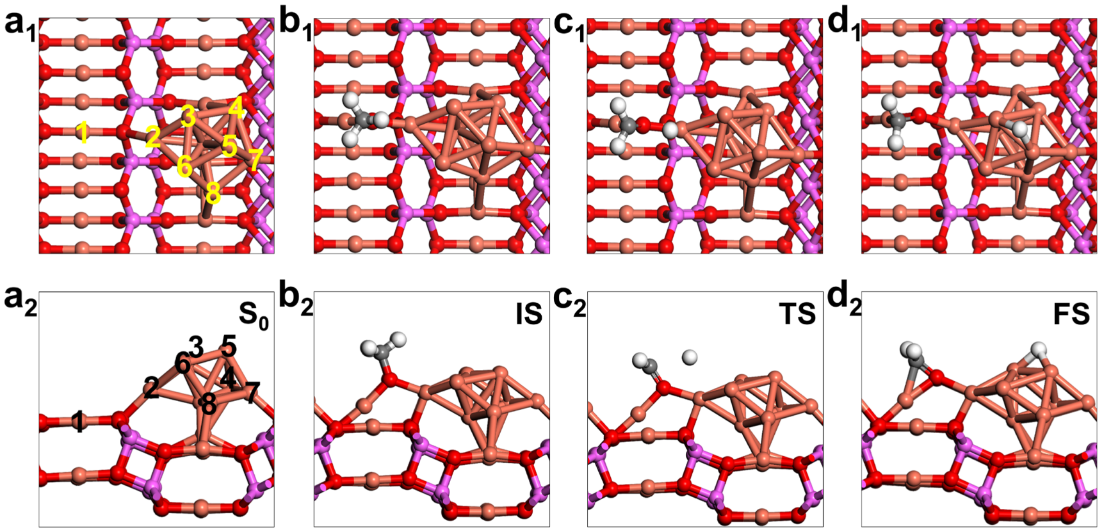

e

Atomic number	S_0	IS	TS	FS
1	+0.54	+0.71	+0.75	+0.72
2	+0.10	+0.33	+0.35	+0.36
3	+0.03	+0.02	+0.04	+0.16
4	-0.03	-0.03	-0.05	-0.04
5	-0.08	-0.03	-0.04	+0.10
6	+0.02	+0.03	+0.05	+0.06
7	+0.05	+0.06	+0.05	+0.09
8	-0.04	-0.03	-0.03	-0.02

Supplementary Figure 97. DFT calculation models and Bader charge analysis of Cu(111)/$CuAlO_2$(101) after adsorbing CH_3O. Bader charge analysis for the C–H bonds cleavage in CH_3O* at the Cu(111)/$CuAlO_2$(101) interface. **a_1–d_1** show the vertical view of initial catalyst structure (S_0), initial adsorption state (IS), transition state (TS) and finally state (FS), respectively. **a_2–d_2** display the front view of initial catalyst structure (S_0), initial adsorption state (IS), transition state (TS) and final state (FS), respectively. **e** Bader charge analysis of different Cu atoms.

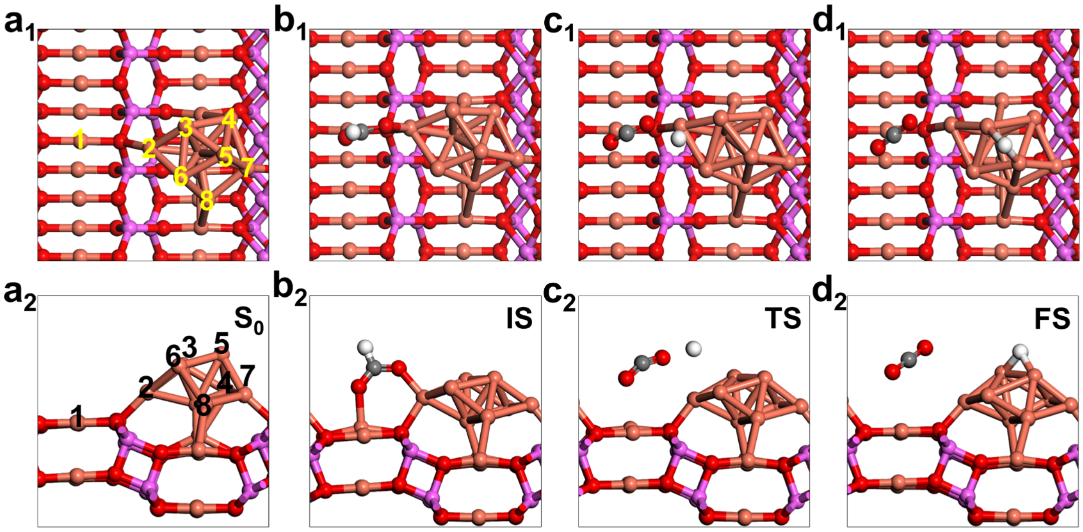

Atomic number	S_0	IS	TS	FS
1	+0.54	+0.72	+0.57	+0.54
2	+0.10	+0.31	+0.26	+0.10
3	+0.03	+0.05	+0.04	+0.14
4	-0.03	+0.02	-0.02	-0.04
5	-0.08	-0.02	-0.04	+0.11
6	+0.02	+0.04	+0.05	+0.05
7	+0.05	+0.09	+0.06	+0.07
8	-0.04	-0.02	-0.04	-0.06

Supplementary Figure 98. DFT calculation models and Bader charge analysis of Cu(111)/$CuAlO_2$(101) after adsorbing HCOO. Bader charge analysis for the C–H bonds cleavage in HCOO* at the Cu(111)/$CuAlO_2$(101) interface. **a_1–d_1** show the vertical view of initial catalyst structure (S_0), initial adsorption state (IS), transition state (TS) and finally state (FS), respectively. **a_2–d_2** displays the front view of initial catalyst structure (S_0), initial adsorption state (IS), transition state (TS) and final state (FS), respectively. **e** Bader charge analysis of different Cu atoms.

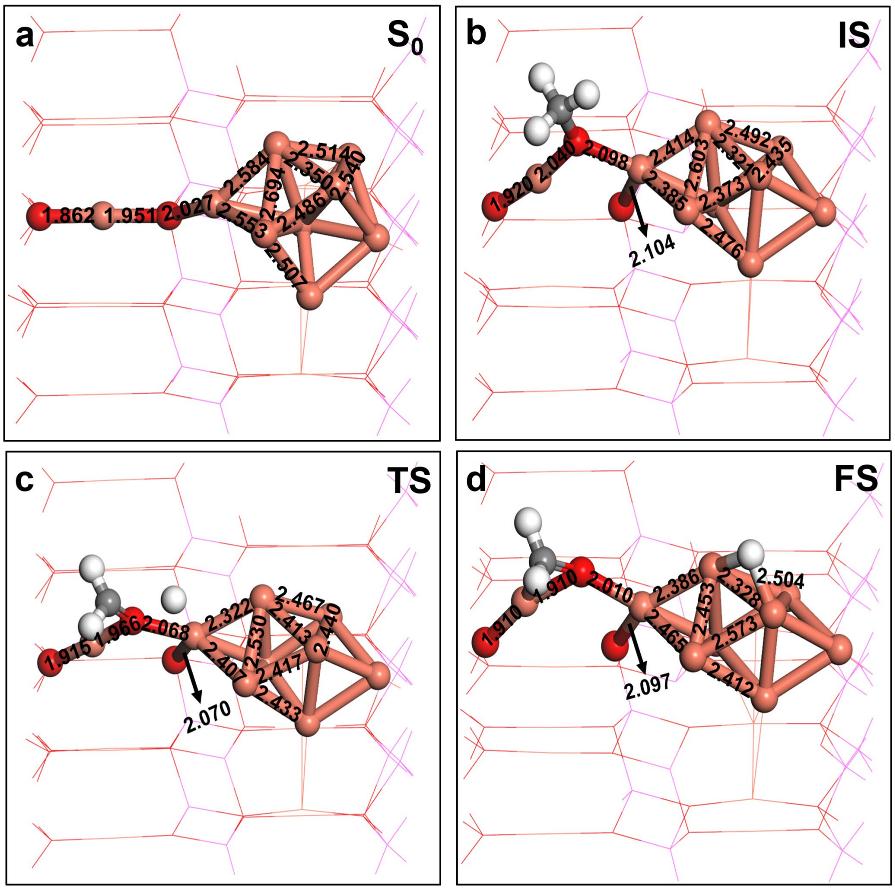

Supplementary Figure 99. Bond length variations from DFT calculation models of Cu(111)/$CuAlO_2$(101) after adsorbing CH_3O. Bond length variations at the Cu(111)/$CuAlO_2$(101) interface during the C–H bonds cleavage in CH_3O*. Compared with the initial catalyst structure, the activation adsorption of CH_3O* intermediate promotes the stretching and compression of the interfacial Cu–O and Cu–Cu bonds. **a–d** represent the initial catalyst structure (S_0), initial adsorption state (IS), transition state (TS) and final state (FS), respectively.

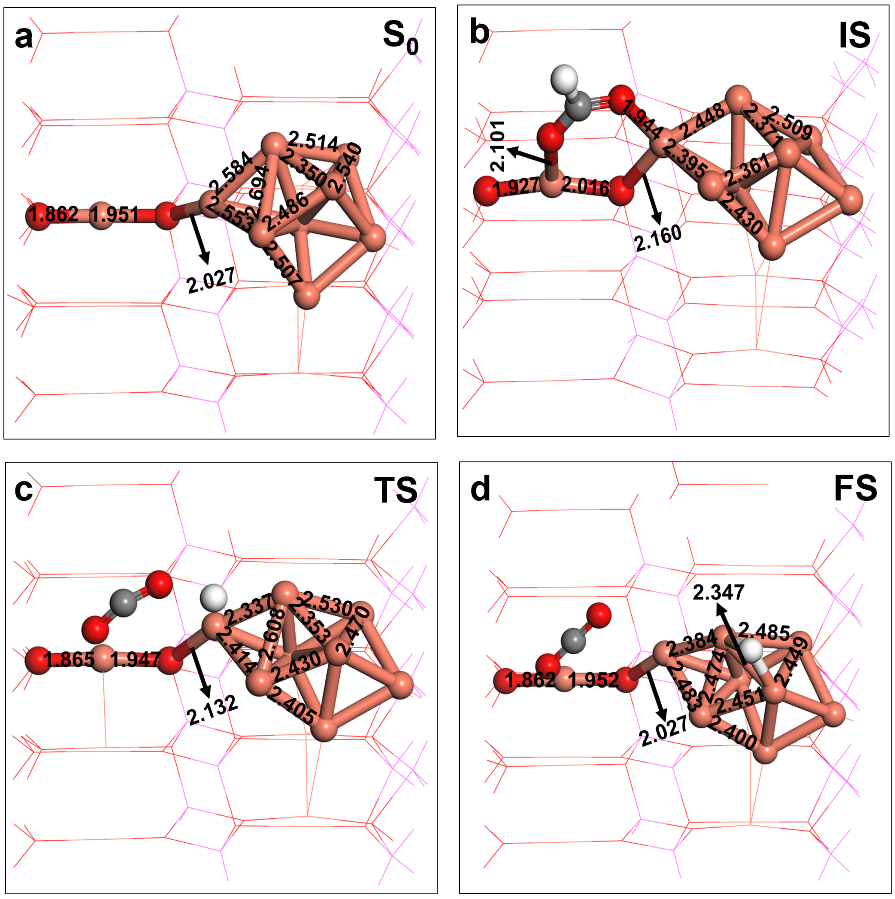

Supplementary Figure 100. Bond length variations from DFT calculation models of Cu(111)/$CuAlO_2$(101) after adsorbing HCOO. Bond length variations at the Cu(111)/$CuAlO_2$(101) interface during the C–H bonds cleavage in HCOO*. Compared with the initial catalyst structure, the activation adsorption of HCOO* intermediate promotes the stretching and compression of the interfacial Cu–O and Cu–Cu bonds. **a–d** represent the initial catalyst structure (S_0), initial adsorption state (IS), transition state (TS) and final state (FS), respectively.

Supplementary tables

Supplementary Table 1. Physicochemical properties of various samples

Sample	m_{Al} (wt%)[a]	m_{Cu} (wt%)[a]	Cu/Al[a]	S_{BET} (m^2/g)[b]	D_{pore} (nm)[b]	V_{pore} (cm^3/g)[b]	d_{Cu} (nm)[c]	C_{Cu^0} (mmol/g)[d]	C_{Cu^+} (mmol/g)[d]	$L_{Cu^0\text{-}Cu^+}$ (10^9 m g^{-1})	Cu^+/Cu^0 (%)[e]
**0.95Cu/Cu(Al)O*x***	16.3	15.5	0.95	104.8	19.0	0.95	7.0	0.247	0.238	3.43	105
**2.32Cu/Cu(Al)O*x***	16.6	38.5	2.32	92.8	5.6	0.22	7.4	0.904	0.430	7.52	77
**3.06Cu/Cu(Al)O*x***	17.8	54.5	3.06	81.2	9.1	0.22	8.0	1.464	0.464	9.12	70
**4.25Cu/Cu(Al)O*x***	12.7	54.0	4.25	39.4	18.5	0.15	8.9	1.707	0.412	10.39	59
**5.27Cu/Cu(Al)O*x***	10.3	54.3	5.27	35.2	29.0	0.25	9.1	1.863	0.279	8.64	49
**7.18Cu/Cu(Al)O*x***	8.2	58.9	7.18	47.4	12.0	0.16	9.3	1.905	0.204	6.39	36
**4.20Cu/Al_2O_3**	13.2	55.5	4.20	73.8	16.8	0.37	9.1	2.241	0.130	4.68	21

[a] Results obtained by ICP-AES analysis.
[b] Results determined by N_2 adsorption analysis.
[c] Results obtained from TEM analysis.
[d] Results determined by N_2O titration and CO-TPD analysis.
[e] Results obtained by *in situ* AES analysis.

Supplementary Table 2. EXAFS fitting parameters at the Cu K-edge for various samples

Sample	Shell	R (Å)[a]	CN[b]	σ^2 (10^{-3}Å^2)[c]	ΔE_0 (eV)[d]	R factor (%)[e]
**Cu-foil**	Cu–Cu	2.54(0.01)	12.0(0.2)	8.5	4.52	0.2
**Cu_2O**	Cu–O	1.81(0.01)	2.0(0.1)	3.7	8.17	2.0
	Cu–Cu	3.01(0.02)	12.0(0.1)	3.4		
**0.95Cu/Cu(Al)O_x**	Cu–O	1.85(0.01)	1.7(0.3)	8.2	5.26	1.6
	Cu–Cu	2.54(0.02)	5.1(0.2)	8.2		
**2.32Cu/Cu(Al)O_x**	Cu–O	1.86(0.02)	1.4(0.2)	9.3	−4.59	0.9
	Cu–Cu	2.54(0.01)	5.8(0.1)	9.3		
**3.06Cu/Cu(Al)O_x**	Cu–O	1.85(0.01)	1.2(0.3)	9.3	−5.84	0.9
	Cu–Cu	2.54(0.02)	6.6(0.1)	9.3		
**4.25Cu/Cu(Al)O_x**	Cu–O	1.84(0.01)	0.9(0.2)	9.1	−3.57	0.8
	Cu–Cu	2.54(0.01)	6.9(0.2)	9.1		
**5.27Cu/Cu(Al)O_x**	Cu–O	1.84(0.01)	0.7(0.2)	9.3	5.09	0.4
	Cu–Cu	2.53(0.02)	7.7(0.4)	9.3		
**7.18Cu/Cu(Al)O_x**	Cu–O	1.83(0.02)	0.5(0.2)	3.5	5.44	0.4
	Cu–Cu	2.53(0.01)	8.2(0.2)	0.5		
**4.20Cu/Al_2O_3**	Cu–O	1.81(0.02)	0.2(0.2)	0	4.62	0.2
	Cu–Cu	2.54(0.01)	10.1(0.1)	8.5		

[a] Bond length.
[b] Coordination number.
[c] Debye-Waller factor.
[d] Inner potential correction.
[e] Goodness of fit.

Supplementary Table 3. EXAFS fitting parameters at the Cu K-edge for various samples

Sample	Shell	*R* (Å)[a]	CN[b]	σ^2 (10^{-3}Å^2)[c]	ΔE_0 (eV)[d]	*R* factor (%)[e]
**Cu-foil**	Cu–Cu	2.54(0.01)	12.0(0.2)	8.5	4.52	0.2
**Cu_2O**	Cu–O	1.81(0.01)	2.0(0.1)	3.7	8.17	2.0
	Cu–Cu	3.01(0.02)	12.0(0.1)	3.4		
**4.25Cu/Cu(Al)O_x-500**	Cu–O	1.84(0.01)	0.9(0.2)	9.1	5.96	1.1
	Cu–Cu	2.54(0.01)	6.9(0.2)	9.1		
	Cu–Al	3.17(0.03)	1.7(0.1)	9.1		
**4.25Cu/Cu(Al)O_x-600**	Cu–O	1.85(0.01)	1.1(0.2)	9.0	3.99	1.5
	Cu–Cu	2.54(0.01)	6.3(0.1)	9.0		
	Cu–Al	3.18(0.02)	2.1(0.2)	9.0		
**4.25Cu/Cu(Al)O_x-700**	Cu–O	1.87(0.02)	1.4(0.2)	8.9	7.64	1.2
	Cu–Cu	2.54(0.01)	5.6(0.3)	8.9		
	Cu–Al	3.18(0.03)	2.5(0.1)	8.9		
**4.25Cu/Cu(Al)O_x-800**	Cu–O	1.89(0.02)	1.7(0.2)	9.6	4.27	1.5
	Cu–Cu	2.55(0.02)	4.9(0.3)	9.6		
	Cu–Al	3.18(0.04)	2.8(0.2)	9.6		

[a] Bond length.
[b] Coordination number (CN).
[c] Debye-Waller factor.
[d] Inner potential correction.
[e] Goodness of fit.

Supplementary Table 4. Comparison study on catalytic performance for MSR reaction over reported catalysts and this work

Catalyst	Temp. (°C)	X_{MeOH} (%)	R_{H_2} (μmol $s^{-1}\cdot g_{cat}^{-1}$)	S_{CO_2} (%)	Ref.
**4.25Cu/Cu(Al)O_x**	240	> 99	110.8	99	This work
**CuZnO/γ-Al_2O_3/Al**	300	95	--[b]	90	13
**$CuMgAl_2O_4$**	250	92	--[b]	96	14
**CuZrAl**	240	95	25.6	99	15
**Cu/SiO_2**	280	80	91.6	--[b]	16
**Cu/Sc_2O_3-ZnO**	240	42	53.0	100	17
**$Cu_{50}Zn_{30}Zr_{10}Al_{10}$**	270	90	--[b]	99	18
**5Cu10Al**	350	97	15.7	99	19
**CuO/ZnO/CeO_2/ZrO_2**	300	90	73.4	-	20
**Ce-Cu/Zn-Al**	280	100	24.3	99	21
**Cu/ZnO/ZrO_2**	250	88	52.7	100	22
**Cu/ZrO_2-SiO_2**	260	73	102.8	--[b]	23
**Cu/ZrO_2**	300	60	--[b]	70	24
**CuPd/ZrO_2**	260	88	24.0	95	25
**Cu-Fe/silicates**	200	100	1.6	--[b]	26
**CuO/ZnO/Ga_2O_3**	240	90	--[b]	--[b]	27
**Cu/ZnO/Al_2O_3**	225	67	--[b]	--[b]	28
**Ru_1/CeO_2**	350	26	38.8	98	29
**Au_1/ZnO**	350	28	--[b]	100	30
**2%Pt/α-MoC[a]**	190	--[b]	129.6	99	31
**2%Ni/α-MoC[a]**	240	--[b]	171.0	99	32
**In_xPd_y/In_2O_3**	300	--[b]	62.6	93	33
**InPd/In_2O_3**	300	90	--[b]	95	34
**Pd/ZnO**	300	97	--[b]	86	35
**Pt/-K@S-1**	250	< 20	12.0	-	36

[a] Aqueous methanol reforming.
[b] Lack of relevant data.

Supplementary Table 5. Fitting results of *in situ* EXAFS spectra of 4.25Cu/Cu(Al)O_x at the Cu K-edge after various atmosphere treatments

Sample	Shell	R (Å)[a]	CN[b]	σ^2 (10^{-3}Å^2)[c]	ΔE_0 (eV)[d]	R factor (%)[e]
**Cu-foil**	Cu–Cu	2.54(0.01)	12.0(0.2)	8.5	4.52	0.2
**Cu_2O**	Cu–O	1.81(0.01)	2.0(0.1)	3.7	8.17	2.0
	Cu–Cu	3.01(0.02)	12.0(0.1)	3.4		
**4.25Cu/Cu(Al)O_x-H_2**	Cu–O	1.84(0.01)	1.2(0.2)	5.4	4.41	2.6
	Cu–Cu	2.54(0.01)	6.2(0.2)	11.7		
**4.25Cu/Cu(Al)O_x-CH_3OH**	Cu–O	1.86(0.01)	1.5(0.1)	4.6	5.46	1.6
	Cu–Cu	2.49(0.02)	5.5(0.2)	12.3		
**4.25Cu/Cu(Al)O_x-H_2O**	Cu–O	1.89(0.01)	1.8(0.2)	5.7	8.15	1.5
	Cu–Cu	2.46(0.02)	5.1(0.1)	11.7		
**4.25Cu/Cu(Al)O_x-He**	Cu–O	1.84(0.01)	1.1(0.2)	5.2	4.68	3.1
	Cu–Cu	2.54(0.02)	6.0(0.2)	11.7		

[a] Bond length.
[b] Coordination number.
[c] Debye-Waller factor.
[d] Inner potential correction.
[e] Goodness of fit.

Supplementary Table 6. Fitting results of *in situ* EXAFS spectra of 7.18Cu/Cu(Al)O_x at the Cu K-edge after various atmosphere treatments

Sample	Shell	R (Å)[a]	CN[b]	σ^2 (10^{-3}Å^2)[c]	ΔE_0 (eV)[d]	R factor (%)[e]
**Cu-foil**	Cu–Cu	2.54(0.01)	12.0(0.2)	8.5	4.52	0.2
**Cu_2O**	Cu–O	1.81(0.01)	2.0(0.1)	3.7	8.17	2.0
	Cu–Cu	3.01(0.02)	12.0(0.1)	3.4		
**7.18Cu/Cu(Al)O_x-H_2**	Cu–O	1.84(0.01)	0.8(0.1)	7.0	4.06	1.1
	Cu–Cu	2.54(0.02)	6.6(0.2)	12.4		
**7.18Cu/Cu(Al)O_x-CH_3OH**	Cu–O	1.85(0.02)	0.8(0.2)	7.0	2.85	1.1
	Cu–Cu	2.55(0.02)	6.5(0.2)	12.4		
**7.18Cu/Cu(Al)O_x-H_2O**	Cu–O	1.84(0.02)	1.0(0.3)	7.1	3.76	1.2
	Cu–Cu	2.54(0.02)	6.4(0.1)	11.7		
**7.18Cu/Cu(Al)O_x-He**	Cu–O	1.85(0.02)	0.9(0.2)	6.1	4.79	1.8
	Cu–Cu	2.53(0.01)	6.5(0.2)	8.4		

[a] Bond length.
[b] Coordination number.
[c] Debye-Waller factor.
[d] Inner potential correction.
[e] Goodness of fit.

Supplementary References

1. Li A, et al. Active Cu^0–$Cu^{\sigma+}$ sites for the hydrogenation of carbon-oxygen bonds over Cu/CeO_2 catalysts. *ACS Catal.* ***12***, 1315–1325 (2022).

2. Góra-Marek K, Palomares AE, Glanowska A, Sadowska K, Datka J. Copper sites in zeolites-quantitative IR studies. *Microporous Mesoporous Mater.* ***162***, 175–180 (2012).

3. Ravel, B. & Newville, M. ATHENA, ARTEMIS, HEPHAESTUS: data analysis for X-ray absorption spectroscopy using IFEFFIT. *J. Synchrotron Radiat.* **12**, 537–541 (2005).

4. Wang, Y., Zhang, H., An, P., Wu, H. & Jia, J. Effect of potassium on methanol steam reforming on the Cu(111) and Cu(110) surfaces: a DFT study. *J. Phys. Chem. C* **125**, 20905–20918 (2021).

5. Gu, X. & Li, W. First-principles study on the origin of the different selectivities for methanol steam reforming on Cu(111) and Pd(111). *J. Phys. Chem. C* **114**, 21539–21547 (2010).

6. Chaves, A.S., Rondina, G.G., Piotrowski, M.J., Tereshchuk, P. & Da Silva, J.L.F. The role of charge states in the atomic structure of Cu_n and Pt_n (n = 2–14 atoms) clusters: a DFT investigation. *J. Phys. Chem. A* **118**, 10813–10821 (2014).

7. Christensen, O.B., Jacobsen, K.W., Nørskov, J.K. & Manninen, M. Cu cluster shell structure at elevated temperatures. *Phys. Rev. Lett.* **66**, 2219–2222 (1991).

8. Long, O.Y., Gautam, G. & Carter, E.A. Evaluating optimal *U* for *3d* transition-metal oxides within the SCAN+*U* framework. *Phys. Rev. Mater.* **4**, 045401 (2020).

9. Maeda, N., Meemken, F., Hungerbühler, K. & Baiker A. Selectivity-controlling factors in catalytic methanol amination studied by isotopically modulated excitation IR spectroscopy. *ACS Catal.* **3**, 219–223 (2013).

10. Larsen, R.W., Zielke, P. & Suhm, M.A. Hydrogen-bonded OH stretching modes of methanol clusters: a combined IR and Raman isotopomer study. *J. Chem. Phys.* **126**, 194307 (2007).

11. Barakoti, K.K. et al. Formaldehyde analysis in non-aqueous methanol solutions by infrared spectroscopy and electrospray ionization. *Front. Chem.* **9**, 678112 (2021).

12. Collins, S.E., Briand, L.E., Gambaro, L., A, Baltanás. M.A. & Bonivardi, A.L. Adsorption and decomposition of methanol on gallium oxide polymorphs. *J. Phys. Chem. C* **112**, 14988–15000 (2008).

13. Zhang, G., Zhao, J., Yang, T., Zhang, Q. & Zhang, L. In-situ self-assembled Cu_2O/ZnO core-shell catalysts synergistically enhance the durability of methanol steam reforming. *Appl. Catal. A* **616**, 118072 (2021).

14. Kamyar, N., Khani, Y., Amini, M., Bahadoran, F. & Safari, N. Copper-based catalysts over A520-MOF derived aluminum spinels for hydrogen production by methanol steam reforming: The role of spinal support on the performance. *Int. J. Hydrogen. Energ.* **45**, 21341–21353 (2020).
15. Mateos-Pedrero, C., Azenha, C., Pacheco Tanaka, D.A., Sousa, J.M. & Mendes, A. The influence of the support composition on the physicochemical and catalytic properties of Cu catalysts supported on Zirconia-Alumina for methanol steam reforming. *Appl. Catal. B* **277**, 119243 (2020).
16. Díaz-Pérez, M.A., Moya, J., Serrano-Ruiz, J.C. & Faria, J. Interplay of support chemistry and reaction conditions on copper catalyzed methanol steam reforming. *Ind. Eng. Chem. Res.* **57**, 15268–15279 (2018).
17. Pu, Y. et al. An improved Cu/ZnO catalyst promoted by Sc_2O_3 for hydrogen production from methanol reforming. *Fuel* 241, 607–615 (2019).
18. Wan, Y. et al. Hydrogen production from steam reforming of methanol over $CuO/ZnO/Al_2O_3$ catalysts: catalytic performance and kinetic modeling. *Chinese. J. Chem. Eng* **24**, 1186–1194 (2016).
19. Mrad, M., Gennequin, C., Aboukaïs, A., Abi-Aad, E. Cu/Zn-based catalysts for H_2 production via steam reforming of methanol. *Catal. Today.* **176**, 88–92 (2011).
20. Liao, M. et al. One-step growth of $CuO/ZnO/CeO_2/ZrO_2$ nanoflowers catalyst by hydrothermal method on Al_2O_3 support for methanol steam reforming in a microreactor. *Int. J. Hydrogen. Energ.* **46**, 9280–9291 (2021).
21. Qiao, W. et al. Performance of Cu-Ce/M-Al (M = Mg, Ni, Co, Zn) hydrotalcite derived catalysts for hydrogen production from methanol steam reforming. *Int. J. Hydrogen. Energ.* **45**, 12773–12783 (2021).
22. Sanches, S.G., Flores, J.H. & Da, Silva, M.I.P. Cu/ZnO and $Cu/ZnO/ZrO_2$ catalysts used for methanol steam reforming. *Mol. Catal.* **454**, 55–62 (2018).
23. Bossola, F. et al. Electron-poor copper nanoparticles over amorphous zirconia-silica as all-in-one catalytic sites for the methanol steam reforming. *Appl. Catal. B* **258**, 118016 (2019).
24. Ploner, K. et al. Mechanistic insights into the catalytic methanol steam reforming performance of Cu/ZrO_2 catalysts by in situ and operando studies. *J. Catal.* **391**, 497–512 (2020).
25. Azenha, C., Lagarteira, T., Mateos-Pedrero, C. & Mendes, A. Production of hydrogen from methanol steam reforming using $CuPd/ZrO_2$ catalysts-influence of the catalytic surface on methanol conversion and CO selectivity. *Int. J. Hydrogen. Energ.* **46**, 17490–17499 (2021).
26. Kuo, M.T et al. Synthesis of mesoporous CuFe/silicates catalyst for methanol steam reforming. *Int. J. Hydrogen. Energ.* **44**, 14416–14423 (2019).
27. Ribeirinha, P., Mateos-Pedrero, C., Boaventura, M., Sousa, J. & Mendes, A. $CuO/ZnO/Ga_2O_3$ catalyst for low temperature MSR reaction: Synthesis, characterization and kinetic model. *Appl.*

Catal. B. **221**, 371–379 (2018).
28. Li, D. et al. Induced activation of the commercial $Cu/ZnO/Al_2O_3$ catalyst for the steam reforming of methanol. *Nat. Catal.* **5**, 99–108 (2022).
29. Chen, L. et al. Insights into the mechanism of methanol steam reforming tandem reaction over CeO_2 supported single-site catalysts. *J. Am. Chem. Soc.* **143**, 12074–12081 (2021).
30. Gu, X. et al. Supported single Pt_1/Au_1 atoms for methanol steam reforming. *ACS Catal.* **4**, 3886–3890 (2014).
31. Lin, L. et al. Low-temperature hydrogen production from water and methanol using Pt/α-MoC catalysts. *Nature* **544**, 80–83 (2017).
32. Lin, L. Atomically dispersed Ni/α-MoC catalyst for hydrogen production from methanol/water. *J. Am. Chem. Soc.* **143**, 309–317 (2021).
33. Köwitsch, N. et al. Proving a paradigm in methanol steam reforming: catalytically highly selective In_xPd_y/In_2O_3 interfaces. *ACS Catal.* **11**, 304–312 (2021).
34. Rameshan, C. et al. Impregnated and Co-precipitated $Pd\text{-}Ga_2O_3$, $Pd\text{-}In_2O_3$ and $Pd\text{-}Ga_2O_3\text{-}In_2O_3$ catalysts: influence of the microstructure on the CO_2 selectivity in methanol steam reforming. *Catal. Lett.* **148**, 3062–3071 (2018).
35. Zeng, Z. et al. A high-performance PdZn alloy catalyst obtained from metal-organic framework for methanol steam reforming hydrogen production. *Int. J. Hydrogen. Energ.* **44**, 24387–24397 (2019).
36. Shao, Z. et al. Maximizing the synergistic effect between Pt^0 and $Pt^{\delta+}$ in a confined Pt-based catalyst for durable hydrogen production. *Appl. Catal. B*. **316**, 121669 (2022).
